# Synthesis and Biological Evaluation of Piperazine Hybridized Coumarin Indolylcyanoenones with Antibacterial Potential

**DOI:** 10.3390/molecules28062511

**Published:** 2023-03-09

**Authors:** Chunmei Zeng, Srinivasa Rao Avula, Jiangping Meng, Chenghe Zhou

**Affiliations:** 1 Institute of Bioorganic & Medicinal Chemistry, Key Laboratory of Applied Chemistry of Chongqing Municipality, School of Chemistry and Chemical Engineering, Southwest University, Chongqing 400715, China; 2 National & Local Joint Engineering Research Center of Targeted and Innovative Therapeutics, IATTI, College of Pharmacy, Chongqing University of Arts and Sciences, Chongqing 402160, China

**Keywords:** coumarin, piperazine, antibacterial, resistance, membrane

## Abstract

A class of piperazine hybridized coumarin indolylcyanoenones was exploited as new structural antibacterial frameworks to combat intractable bacterial resistance. Bioactive assessment discovered that 4-chlorobenzyl derivative **11f** showed a prominent inhibition on *Pseudomonas aeruginosa* ATCC 27853 with a low MIC of 1 μg/mL, which was four-fold more effective than norfloxacin. Importantly, the highly active **11f** with inconspicuous hemolysis towards human red blood cells displayed quite low proneness to trigger bacterial resistance. Preliminary explorations on its antibacterial behavior disclosed that **11f** possessed the ability to destroy bacterial cell membrane, leading to increased permeability of inner and outer membranes, the depolarization and fracture of membrane, and the effusion of intracellular components. Furthermore, bacterial oxidative stress and metabolic turbulence aroused by **11f** also accelerated bacterial apoptosis. In particular, **11f** could not only effectively inset into DNA, but also bind with DNA gyrase through forming supramolecular complex, thereby affecting the biological function of DNA. The above findings of new piperazine hybridized coumarin indolylcyanoenones provided an inspired possibility for the treatment of resistant bacterial infections.

## 1. Introduction

Antibiotics, as powerful weapons, have been playing quite important roles in the fight against bacteria [1,2]. However, the long-term abuse and misuse of antibiotics has gradually made them accomplices in the evolution of drug-resistant bacteria, posing a great threat to human health [3,4]. Currently, almost all types of antibiotics have developed drug resistance, and few new classes of antibiotics have been approved for clinical use in the past three decades, giving rise to an alarming increase in mortality from bacterial infections and a serious burden on the global healthcare system [5,6,7]. Therefore, it is extremely urgent to develop a new class of antibacterial molecular entities with a unique chemical scaffold and new mode of action to cope with this severe resistance crisis [8,9,10].

Coumarins with a benzopyrone structure are able to interact with many active sites in organisms through noncovalent interactions, displaying a wide range of bioactivities, which have attracted great attention in the field of medicinal chemistry [11,12,13]. Coumarins are capable of inhibiting the ATPase activity of bacterial DNA gyrase by competing with ATP to bind to the B subunit of the enzyme, affecting the replication of bacterial DNA [14,15,16]. Moreover, many coumarin derivatives with remarkable antibacterial potentials have been found in recent years through extensive structural modification of the coumarin skeleton [17,18,19]. The above findings indicated that the coumarin core is a promising chemical scaffold for the discovery of new antibacterial leads.

**Figure 1:**
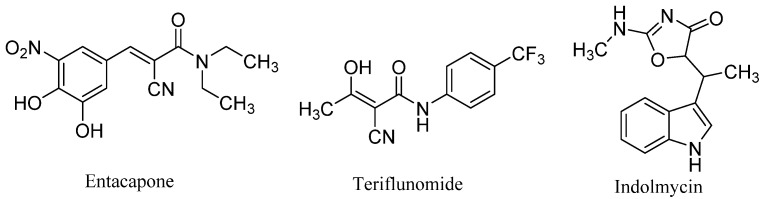
Structures of some cyanoenone and indole derivatives.

The incorporation of different pharmacophores in a molecular framework is beneficial to increase the action sites with enzymes and receptors, which is expected to reduce drug resistance and enhance the antibacterial potential through multitargeting actions. α, β-unsaturated enone as an electrophilic segment is commonly utilized to construct diversified functional scaffolds, such as chalcones [20,21,22]. Cyanoenone is an extended fragment of enone, in which cyano and carbonyl groups have the potential to interact with DNA or enzymes through hydrogen bonds, exists in various drugs, such as entacapone, teriflunomide (Figure 1), and bardoxolone, exhibiting a broad range of pharmacological activities. Indole is a fused nitrogen-containing heterocycle, which can be used as a signal molecule to modulate multiple biological processes, including bacterial pathogenesis [23,24,25], and has been presented in many important pharmaceuticals, such as indolmycin, daptomycin, and indomethacin, displaying good pharmacokinetic properties [26,27,28]. Hence, introducing indolylcyanoenone composed of indole and cyanoenone into the coumarin scaffold might be an effective strategy to construct a new antibacterial framework against drug-resistant bacteria. Piperazine is a classical pharmaceutical linker, which was applied to connect coumarin and indolylcyanoenone, that could regulate the molecular conformations and affect the spatial binding sites with targets [29,30,31]. Besides, the incorporation of various electron-withdrawing and electron-donating substituents on the benzene ring of indole could change the electron cloud density distribution of the indolylcyanoenone conjugated system, which might affect the antibacterial performances of target molecules. The introduction of hydrophobic substituents on the *N*-1 position of indole could improve molecular liposolubility in favor of insertion into the phospholipid bilayer of the bacterial membrane [32,33,34], while the functional hydrophilic groups could easily form hydrogen bonds, and the introduction was beneficial to ameliorate molecular solubility.

**Figure 2:**
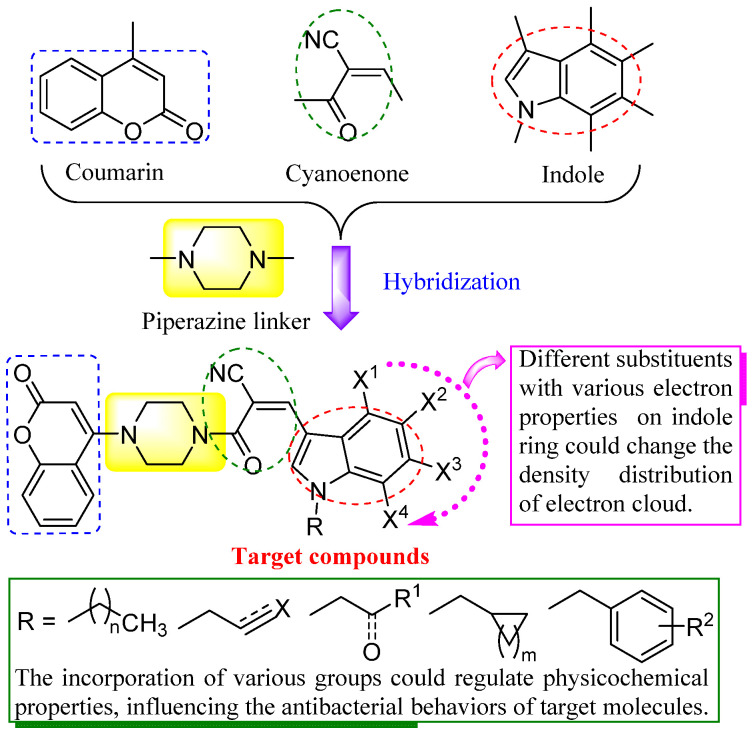
Design of piperazine hybridized coumarin indolylcyanoenones as new structural antibacterial agents.

Considering the above-mentioned factors, a series of new structural piperazine hybridized coumarin indolylcyanoenones (PHCIs) were designed and synthesized (Figure 2), which were expected to act as multitargeting antibacterial agents. The antibacterial performances of all the newly prepared PHCIs were assessed. The resistance tendency and hemolytic activity of highly active PHCI were tested. Moreover, the possible action mechanism of the active molecule was preliminarily investigated, including bacterial cell membrane damage, leakage of cellular contents, oxidative stress, metabolic activity, DNA binding study, and interaction with DNA gyrase.

## 2. Results and Discussion

### 2.1. Chemistry

**Scheme 1:**
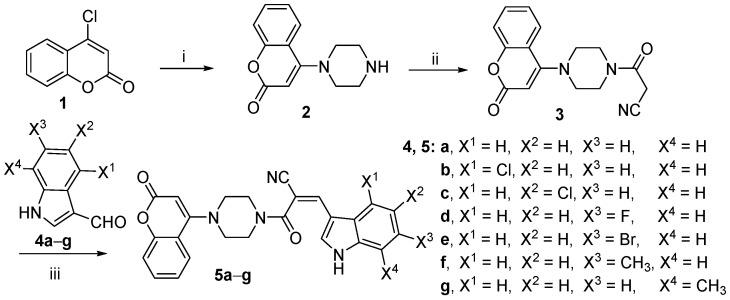
Synthesis of *N*-unsubstituted piperazine hybridized coumarin indolylcyanoenones **5a–g**. Reagents and conditions: (i) EtOH, piperazine, rt; (ii) DMF, cyanoacetic acid, HBTU, DIPEA, rt; (iii) EtOH, piperidine, **4a–g**, 80 °C.

**Scheme 2:**
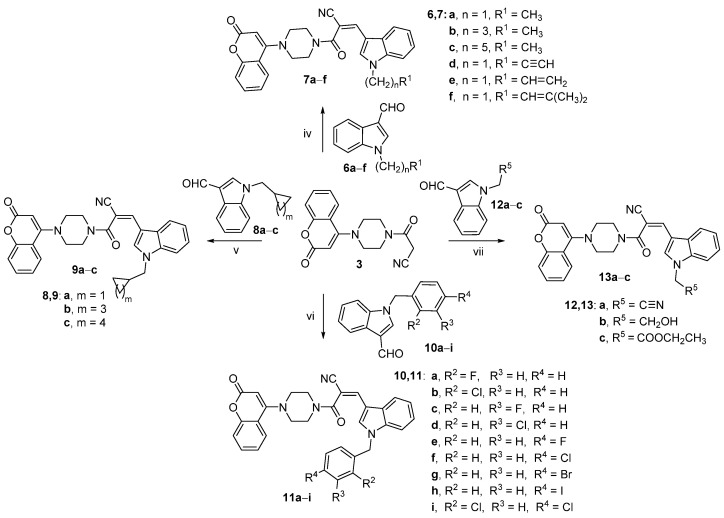
Synthesis of alkyl and unsaturated hydrocarbyl piperazine hybridized coumarin indolylcyanoenones **7a–f**, cycloalkyl piperazine hybridized coumarin indolylcyanoenones **9a–c**, benzyl piperazine hybridized coumarin indolylcyanoenones **11a–i,** and hydrophilic potential piperazine hybridized coumarin indolylcyanoenones **13a–c**. Reagents and conditions: (iv) EtOH, piperidine, **6a–f**, 80 °C; (v) EtOH, piperidine, **8a–c**, 80 °C; (vi) EtOH, piperidine, **10a–i**, 80 °C; (vii) EtOH, piperidine, **12a–c**, 80 °C.

The synthetic paths of new piperazine hybridized coumarin indolylcyanoenones were described in Scheme 1 and Scheme 2. The 4-chloro coumarin **1** was reacted with piperazine to produce piperazine coumarin **2**, which was condensed with cyanoacetic acid to afford crucial amide intermediate **3**. The desired *N*-unsubstituted PHCIs **5a**–**g** were prepared with 63.6–78.8% yields by refluxing intermediate **3** and various *N*-unsubstituted indole aldehydes **4a**–**g** under the catalysis of piperidine in ethanol (Scheme 1). With the aim to explore the effect of different *N*-1 substituents of indole on antibacterial activity, amide coumarin **3** was subsequently condensed with alkyl indole aldehydes **6a**–**c**, unsaturated hydrocarbyl indole aldehydes **6d**–**f**, cycloalkyl indole aldehydes **8a**–**c**, benzyl indole aldehydes **10a**–**I**, and hydrophilic potential indole aldehydes **12a**–**c,** respectively, to acquire corresponding alkyl PHCIs **7a**–**c**, unsaturated hydrocarbyl PHCIs **7d**–**f**, cycloalkyl PHCIs **9a**–**c**, benzyl PHCIs **11a**–**i,** and hydrophilic potential PHCIs **13a**–**c** with yields ranging from 61.5% to 85.7% through the similar preparation processes to *N*-unsubstituted products **5a**–**g** (Scheme 2).

All the structures of target compounds were confirmed by NMR and HRMS analyses. The corresponding spectra were available in Supplementary Materials. In the ^1^H-^1^H COSY spectrum, the highly active molecule PHCI **11f** gave the coupled signal (7.81,7.37) of coumarin-5-*H* (δ, 7.81) and coumarin-6-*H* (δ, 7.37) as well as the coupled signal (7.41,7.61) of coumarin-7-*H* (δ, 7.61) and coumarin-8-*H* (δ, 7.41). Meanwhile, the peaks of (7.26,7.99) and (7.61,7.29) can be attributed to the coupling of indole-4-*H* (δ, 7.99) and indole-5-*H* (δ, 7.26) and the coupling of indole-6-*H* (δ, 7.29) and indole-7-*H* (δ, 7.61), respectively. Moreover, the coupling of benzene-3,5-2*H* (δ, 7.43) and benzene-2,6-2*H* (δ, 7.31) can also be seen from the ^1^H-^1^H COSY spectrum. In addition, carbon and hydrogen atoms all show corresponding ^13^C-^1^H coupled signals in ^1^H-^13^C HMQC and ^1^H-^13^C HMBC spectra, which further confirmed the analysis of NMR spectra.

### 2.2. Antibacterial Activity

All the newly synthesized PHCIs were evaluated for antibacterial activities using a two-fold serial dilution technique with norfloxacin as the positive control; the antibacterial data were shown in Table 1.

The incorporation of different groups on indole core could change the electron cloud distribution of indolylcyanoenone conjugated system, which might affect the noncovalent interactions between whole molecules and biological targets, so the preliminary structural modifications were focused on the benzene ring of indole. Among *N*-unsubstituted PHCIs **5a**–**g**, 6-bromo PHCI **5e** exhibited favorable anti-*S. aureus* 26,003 activity (MIC = 4 μg/mL) and anti-*S. aureus* 25,923 activity (MIC = 2 μg/mL), and 6-methyl derivative **5f** exerted excellent inhibitory activity against *P. aeruginosa* (MIC = 2 μg/mL), which was two-fold more active than norfloxacin. To a certain extent, the incorporation of substituents on indole core could enhance the antibacterial efficacy, especially when introducing bromine atom or methyl group into the *C*-6 position of indole.

The further structural modifications were turned into the easily modified *N*-site of indole in an attempt to exploit more promising antibacterial molecules. When alkyl chains were linked to the *N*-site, it resulted in a near loss of activities towards the tested Gram-positive bacteria but retained considerable anti-*P. aeruginosa* and anti-*P. aeruginosa* 27853 activities. With regard to the unsaturated hydrocarbyl modifications, the most outstanding PHCI was the isopentenyl one **7f**, which showed significant activities against Gram-positive bacteria, being capable of effectively restraining the growth of *S. aureus* 26003 and *S. aureus* 25923 at low concentrations.

Replacing unsaturated hydrocarbyl chains with cycloalkyl segments, a certain improvement of anti-*S. aureus* 6538 potencies was observed, particularly the anti-*S. aureus* 6538 activity of cyclohexyl PHCI **9c** decreased the MIC value to 4 μg/mL. It is worth noting that no matter how the substituents varied, the abilities of PHCIs in inhibiting the growth of *P. aeruginosa* and *P. aeruginosa* 27,853 were always maintained in a moderate to good level, similar to those of norfloxacin.

As for the series of benzyl PHCIs **11a**–**i**, the position of substituents on benzene ring played an important role in exerting antibacterial activity, for example, fluorine atom in the *meta*-position of the benzene ring (**11c**) was favored for combating *S. aureus* and *S. aureus* 6538 with the same MIC of 4 μg/mL, while transferring the fluorine atom to the *para*-position (**11e**) promoted the anti-*P. aeruginosa* 27853 capacity (MIC = 2 μg/mL), which was superior to norfloxacin. When other halogen atoms (Cl, Br, or I) were introduced into the *para*-position of benzene ring, there was slight effect on the ability to inhibit the growth of *P. aeruginosa* series. The lowest MIC value (1 μg/mL) in repressing *P. aeruginosa* 27853 derived from PHCI **11f**, being four-fold more effective than norfloxacin. The 2,4-dichlorobenzyl **11i** suppressed the growth of *P. aeruginosa* and *P. aeruginosa* 27,853 with the same low MIC (2 μg/mL), which were both lower than those of the reference drug. By comparing 4-chlorobenzyl **11f** and 2,4-dichlorobenzyl **11i**, it was found that the augment of chlorine atom on the benzene ring could indeed enhance the ability to inhibit *P. aeruginosa* but would degrade the efficacy to restrain *P. aeruginosa* 27853.

For the purpose of increasing the structural diversity of side chain, functional hydrophilic fragments containing cyanomethyl, hydroxyethyl, as well as ethoxycarbonyl groups were also introduced into *N*-position of indole. Noticeably, PHCI **13a** bearing cyanomethyl segment presented favorable inhibition potency towards *S. aureus* with a low MIC of 4 μg/mL, and hydroxyethyl derivative **13b** and ethoxycarbonyl derivative **13c** both showed appreciable anti-*S. aureus* 25923 potentials (MIC = 8 μg/mL), which might be ascribed to good affinities for biological targets. In general, the bioactivities of PHCIs against Gram-negative bacteria were better than those against Gram-positive bacteria, especially for the *P. aeruginosa* series. Considering the above analyses, the 4-chlorobenzyl derivative **11f** exhibited the best anti-*P. aeruginosa* efficiency, so it was selected as a representative compound for further druggability evaluation and antibacterial mechanism exploration.

### 2.3. Tendency of Coumarin **11f** to Induce Bacterial Resistance

**Figure 3:**
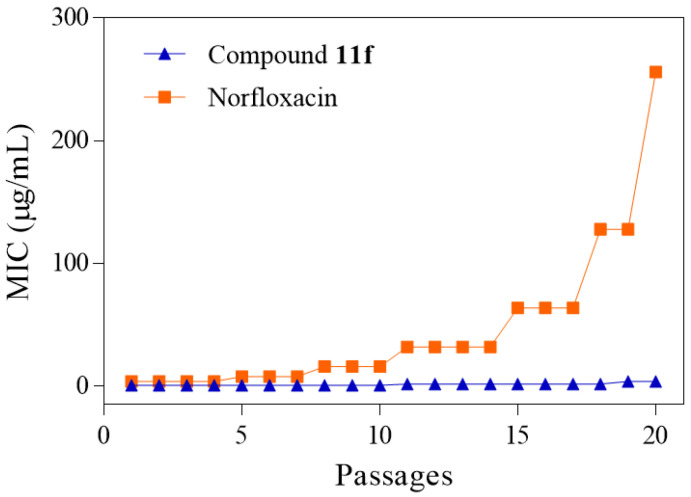
Resistance development of *P. aeruginosa* 27,853 towards molecule **11f** and norfloxacin in 20 days.

The prevalence of bacterial resistance is the primary cause for the decline of available antibiotics and the initial impetus for the development of new antibacterial chemical entities [35,36,37,38]. Therefore, it is necessary to investigate whether the most promising PHCI **11f** could spark rapid bacterial resistance. A serial passaging resistance study of derivative **11f** against *P. aeruginosa* 27,853 strain was conducted and norfloxacin was applied as a positive control. Over the course of 20 days, the resistance level of *P. aeruginosa* 27,853 (Figure 3) for coumarin-derived **11f** almost remained constant, while the resistance for norfloxacin gradually increased after the fifth passage, indicating that the strain was difficult to develop resistance to **11f**.

### 2.4. Hemolytic Activity

**Figure 4:**
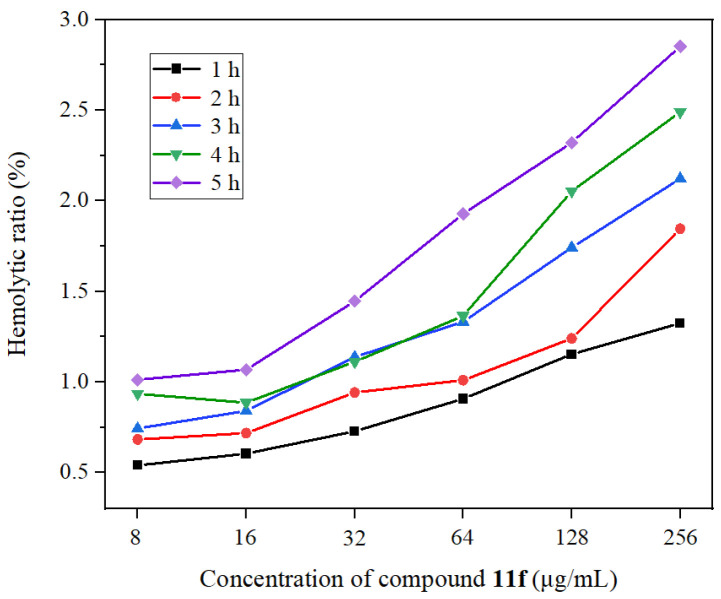
Hemolytic ratio of human red blood cells caused by indolylcyanoenone **11f**.

The safety of drug candidates on mammalian cells is an important indicator to evaluate their clinical application potential [39,40,41]. Herein, a hemolytic assay of 4-chlorobenzyl PHCI **11f** towards human red blood cells (RBCs) was performed with physiological saline as a negative control and 1% Triton as a positive control [42,43]. As displayed in Figure 4, after incubating with **11f** at increasing concentrations (8, 16, 32, 64, 128, and 256 μg/mL) for different times, the hemolytic rates were all lower than the highest international standard of 5%, revealing the relative biosafety of PHCI **11f** to RBCs, even at high antibacterial concentrations. This manifested that **11f** could selectively act on bacteria whilst having little damage to RBCs, which might be assigned to the difference in the content and electric properties of membrane phospholipids between bacteria and mammalian cells.

### 2.5. Membrane Disruption

The cytoplasmic membrane is a vital shield for bacteria to prevent the entrance of extracellular substances and maintain the homeostasis of intracellular environment. Destroying the integrity of cell membrane can seriously interfere the physiological activities of bacteria, leading to the bacterial death [44,45,46]. Therefore, it was necessary to evaluate the effect of coumarin indolylcyanoenone **11f** on the bacterial cell membrane, including membrane permeability, membrane polarization, as well as the membrane morphology.

#### 2.5.1. Outer Membrane Permeability

**Figure 5:**
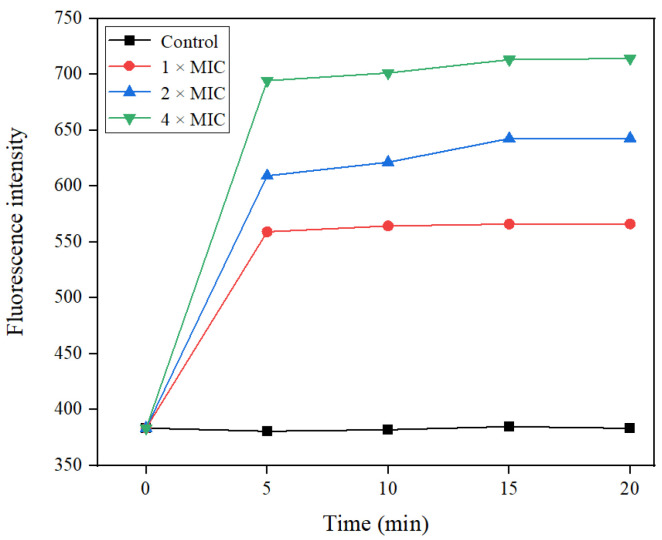
Outer membrane permeability of *P. aeruginosa* 27,853 treated by coumarin **11f**.

The outer membrane is the first barrier of bacteria to resist external attacks. The breakage of the bacterial outer membrane can result in the increment of membrane permeability, facilitating the penetration of antibiotics [47,48]. The hydrophobic dye *N*-phenyl naphthylamine (NPN) was adopted to research the outer membrane permeabilization of *P. aeruginosa* 27,853 after the treatment of PHCI **11f [49,50]**. As shown in Figure 5, a significant increase in fluorescence emitted by the NPN dye were observed in concentration- and time-dependent modes. This indication suggested that 4-chlorobenzyl derivative **11f** was able to compromise the *P. aeruginosa* 27,853 outer membrane immediately, which might be attributed to its good lipophilicity making it easy to penetrate the lipid bilayer of the bacterial membrane.

#### 2.5.2. Inner Membrane Permeability

**Figure 6:**
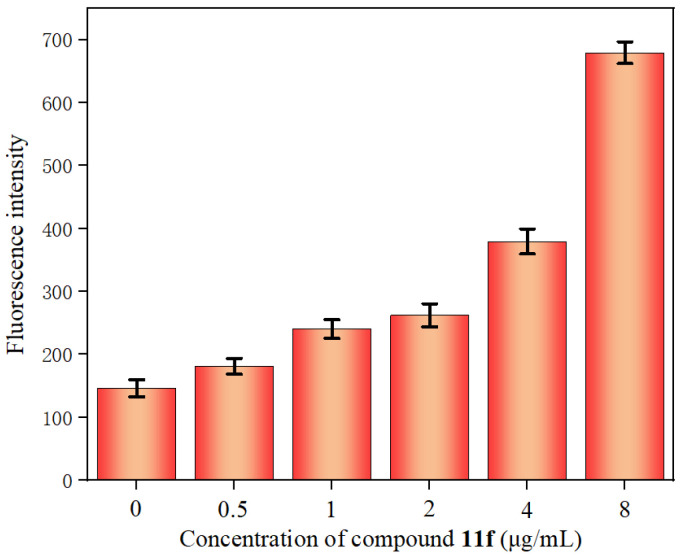
Inner membrane permeability of *P. aeruginosa* 27,853 caused by compound **11f**.

The inner membrane is also an effective permeability barrier for Gram-negative bacteria. To explore the effect of derivative **11f** on the *P. aeruginosa* 27,853 inner membrane, propidium iodide (PI), a common penetrable dye that could penetrate the compromised membrane and combine with intracellular nucleic acid to emit intense red fluorescence, was applied to detect the inner membrane permeability [51,52,53]. As depicted in Figure 6, the fluorescence intensity gradually raised upon the treatment of increasing amounts of **11f**, especially at 8 μg/mL, where the fluorescence change was the most remarkable. This performance was consistent with that of the outer membrane, implicating that PHCI **11f** could effectively enhance *P. aeruginosa* 27,853 membrane permeability from outer membrane to the inner membrane.

#### 2.5.3. Membrane Depolarization

**Figure 7:**
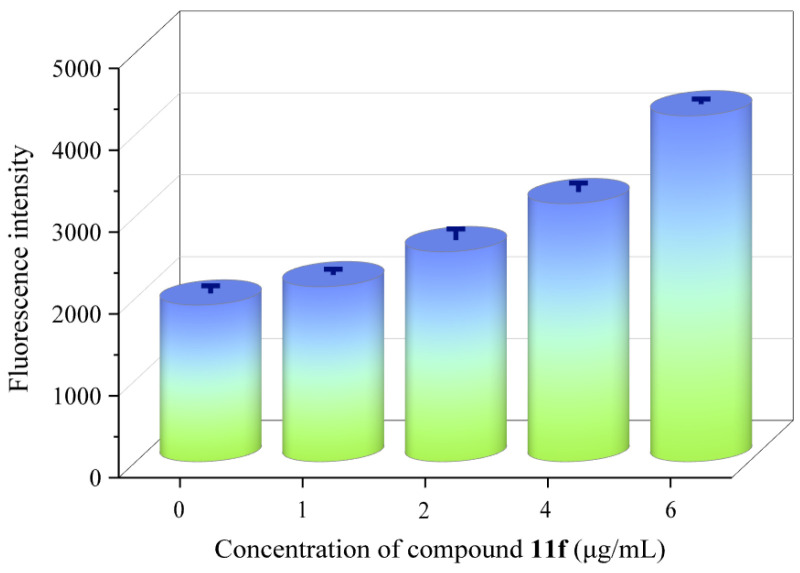
The depolarization assay of *P. aeruginosa* 27,853 in the presence of varying concentrations of derivative **11f**.

The stability of the bacterial transmembrane potential is closely connected to normal life activities. DiSC3(5) fluorescent dye can be absorbed by polarized cells and gathered in the phospholipid bilayer accompanied by fluorescence self-quenching. Once the membrane potential is disturbed, the dyes are released from cells and give off strong fluorescence, providing a manner to measure bacterial membrane potential [54,55,56]. As exhibited in Figure 7, after incubation with different concentrations of coumarin **11f**, the fluorescence intensity presented a dose-dependent increase, indicating that PHCI **11f** could obviously dissipate the membrane potential and lead to membrane depolarization.

#### 2.5.4. Cell Morphology Observation

**Figure 8:**
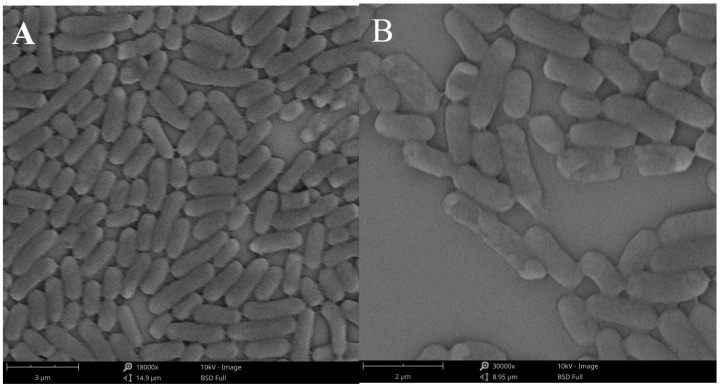

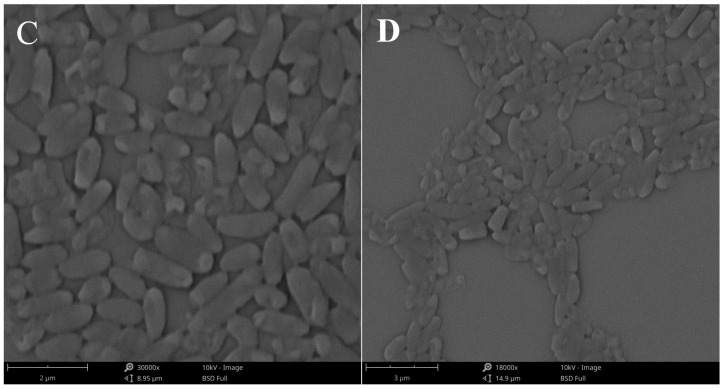
The SEM images of *P. aeruginosa* 27,853 without treatment (**A**) and treated with 4-chlorobenzyl **11f** at 2 μg/mL for 3 h (**B**), 6 h (**C**), and 12 h (**D**).

To further search for the proof of physical disruption of *P. aeruginosa* 27,853 cell membrane, scanning electron microscope (SEM) was employed to visually observe the change in cell morphology and integrity with or without the treatment of **11f**. In Figure 8A, the vast majority of *P. aeruginosa* 27,853 cells without any treatment were intact and plump, reflecting the normal physiological status of the bacteria. Whereas after treatment with PHCI **11f**, the cells showed significant deformation or even rupture, and the proportion of deformed and ruptured cells gradually increased (Figure 8B,C) with the extension of the **11f** treatment time (3, 6 and 12 h). The above results confirmed that PHCI **11f** could indeed destroy the cell membrane, which might be an important reason for its powerful germicidal effect.

### 2.6. Leakage of Intracellular Components

**Figure 9:**
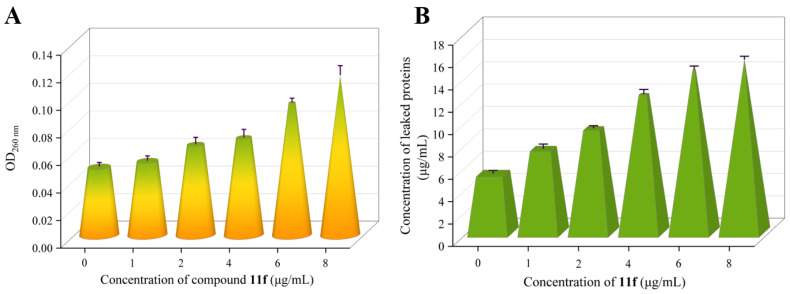
Intracellular nucleic acids (**A**) and proteins (**B**) leakage following treatment with the 4-chlorobenzyl derivative **11f**.

The damage of cell membrane integrity is predicted to result in the effusion of intracellular crucial biological macromolecules, such as nucleic acids and proteins [57,58,59]. Nucleic acids have a characteristic ultraviolet absorption at 260 nm, so the outflow of nucleic acids can be determined by measuring the optical density value of 260 nm [60,61]. As expected, the OD_260_ values of leaked nucleic acids increased significantly from 0.049 to 0.115 in response to treatment of PHCI **11f** at 8 μg/mL (Figure 9A). Meanwhile, a similar phenomenon was also observed in the amount of leaked proteins. The leakage of intracellular proteins from *P. aeruginosa* 27,853 after being treated with **11f** at 8 μg/mL was enhanced by three-fold compared to the untreated group (Figure 9B). These results further indicated that PHCI **11f** destroyed bacterial membrane, inducing the leakage of cellular components.

### 2.7. Oxidative Stress Assay

**Figure 10:**
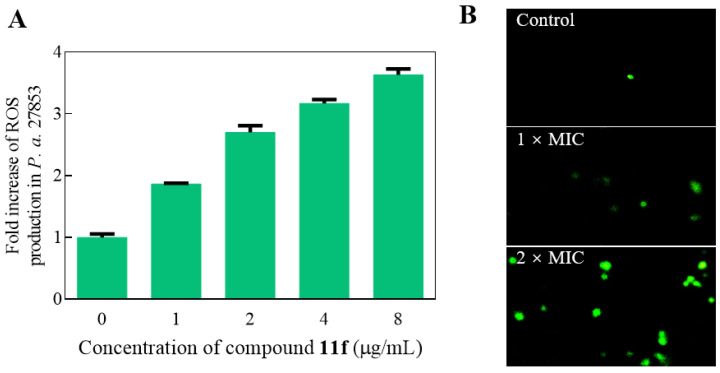
The cellular ROS production (**A**) and fluorescent images (**B**) of *P. aeruginosa* 27,853 exposed to different concentrations of compound **11f**. Scale bar for all fluorescent images was 100 μm.

Endogenously generated reactive oxygen species (ROS) have important signaling roles in bacteria. When excessive ROS levels exceed the cellular scavenging ability, they are particularly active to attack biological macromolecules such as DNA, protein, and lipids [62,63]. To examine the ROS levels of *P. aeruginosa* 27,853 cells treated by **11f**, dichlorofluorescein diacetate (DCFH-DA) was employed as a fluorometric dye. The DCFH-DA probe itself has no fluorescence, but it can be oxidized by ROS into green fluorescent dichlorofluorescein (DCF), and the fluorescence intensity was proportional to ROS levels [64,65]. As seen in Figure 10, after incubating with increasing concentrations of the molecule **11f**, the fluorescence intensity in *P. aeruginosa* 27,853 escalated and the cells stained with green fluorescence became gradually dense, which validated that coumarin **11f** could trigger an endocellular ROS burst. The above findings suggested that damage of the bacterial membrane was not the only mode of action for bacterial death; PHCI **11f** could also promote cell apoptosis by arousing bacterial oxidative damage.

### 2.8. Metabolic Activity

**Figure 11:**
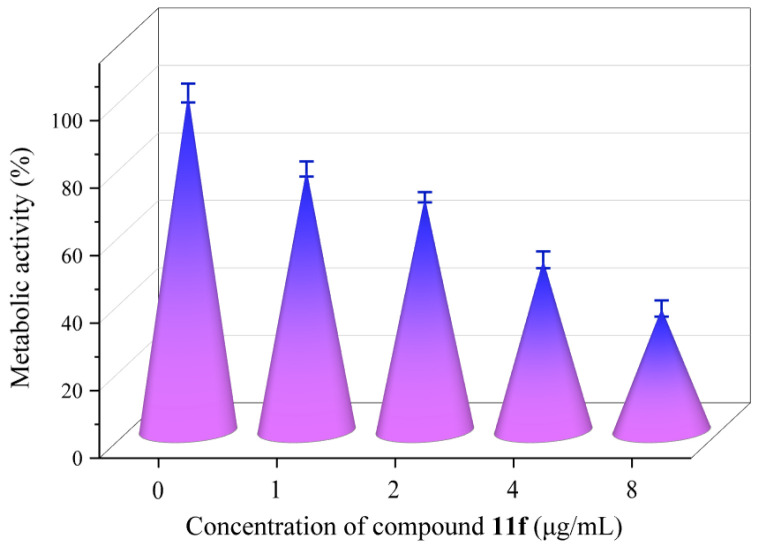
The metabolic activity of *P. aeruginosa* 27,853 influenced by molecule **11f**.

Given that membrane destruction and oxidative damage might affect the bacterial vital biological functions, such as metabolism, alamar blue (resazurin) as an indicator was utilized to detect the metabolic activities of bacterial cells. The detection is based on the capacity of metabolically active cells to transform nonfluorescent resazurin into pink fluorescent resofurin [66,67]. In Figure 11, the metabolic vitality of *P. aeruginosa* 27,853 cells was evidently inhibited upon PHCI **11f** exposure, especially at 8 μg/mL, where the percentage declined to 36%. This result demonstrated that PHCI **11f** could render bacteria functionally abnormal, leading to metabolic disorder and loss of cell viability.

### 2.9. Interactions of **11f** with DNA

#### 2.9.1. DNA Binding Study

**Figure 12:**
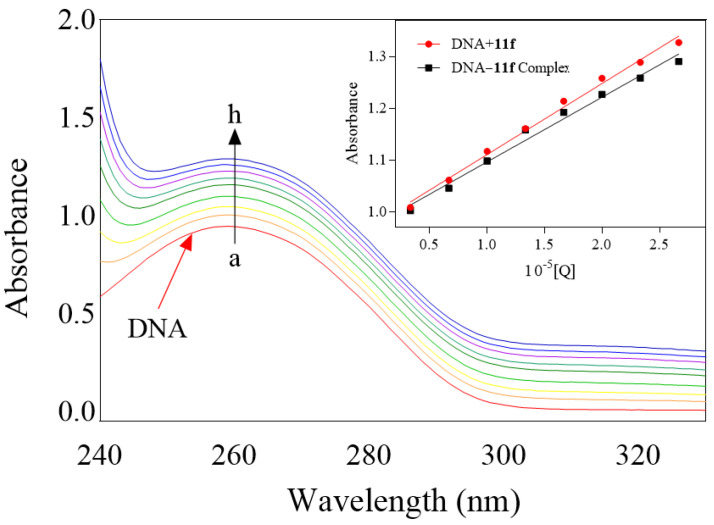
The absorption spectra of DNA in the presence of coumarin indolylcyanoenone **11f**. c(DNA) = 7.1 × 10^−5^ mol·L^−1^, c(**11f**)/(10^−5^ mol·L^−1^), a–h: 0–1.333 (increment: 0.167). Inset: Absorption comparison at 260 nm between the DNA–**11f** complex and the simple addition of DNA alone alone **11f** alone.

DNA is a crucial functional macromolecule and has been broadly investigated as an antibacterial target. The preliminary interaction between 4-chlorobenzyl PHCI **11f** and DNA could be observed through ultraviolet absorption spectrum. When the concentration of DNA was fixed, the characteristic absorption peak of DNA at 260 nm presented a gradient augment with the increasing amounts of **11f** (Figure 12). Additionally, the simple additions of the absorption values of alone DNA and alone **11f** were greater than those of DNA–**11f** complex, manifesting the presence of the hypochromism effect when the active molecule **11f** binds to DNA.

#### 2.9.2. Binding Model between **11f** and DNA

**Figure 13:**
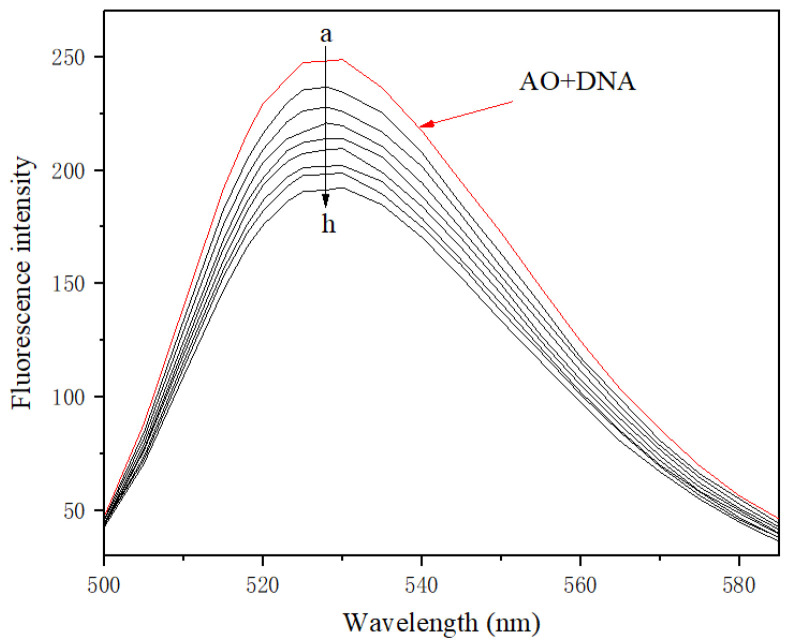
Fluorescence variation of DNA–AO system with increased coumarin **11f**. c(DNA) = 7.1 × 10^−5^ mol·L^−1^, c(**11f**)/(10^−5^ mol·L^−1^), a–h: 0–1.333 (increment: 0.167).

To further study the possible bonding pattern between molecule **11f** and DNA, acridine orange (AO) was selected as a spectral probe, which was capable of intercalating into DNA [68,69,70]. It could be observed, from Figure 13, that the fluorescence intensity of the DNA–AO system at 537 nm decreased clearly with the increasing concentrations of PHCI **11f**, implying that **11f** could effectively inset into DNA by competing with AO for the binding sites on DNA. The aforementioned indication meant that compound **11f** might affect DNA replication through forming a stable DNA–**11f** complex, thus exerting a preeminent bactericidal efficacy.

### 2.10. Molecular Docking

**Figure 14:**
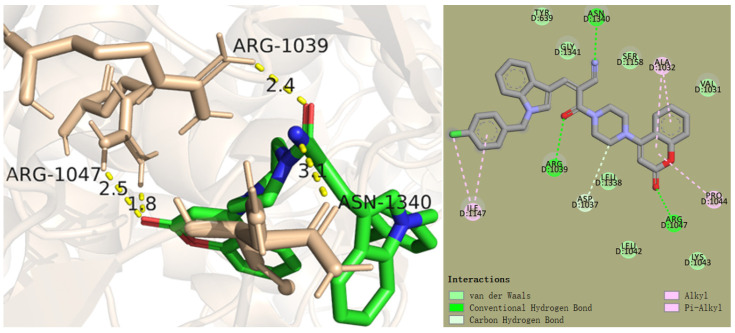
3D (**left**) and 2D (**right**) conformational images of DNA gyrase (PDB: 2XCS) docked with derivative **11f**.

Bacterial DNA gyrase is a well-known target for coumarin-based antibiotics. Molecular docking simulation was performed to clearly clarify the binding behavior between coumarin-derived **11f** and DNA gyrase. As seen from Figure 14, the oxygen atom of the carbonyl group on the coumarin core could interact with residue ARG-1047 by hydrogen bonding. Moreover, alkyl or π-alkyl interactions were also found between the coumarin ring and residues ALA-1032 and PRO-1044. The oxygen atom of enone and the nitrogen atom of the cyano group could form hydrogen bonds with ARG-1039 and ASN-1340, respectively. The substituted 4-chlorobenzyl group also generated alkyl or π-alkyl effects with ILE-1147. The above supramolecular action modes might be helpful to stabilize the **11f**–enzyme complex, affecting the normal function of DNA gyrase.

To compare whether the change of substituents would affect the binding behavior with gyrase, docking simulations of PHCIs **5a**, **5b**, **7a**, **9a,** and **13a** with the enzyme were further performed. As shown in Figures S1–S5, diverse substituents could alter the spatial binding positions of PHCIs and the enzyme, resulting in different binding sites and modes. In general, there were still hydrogen bonds between the enzyme and PHCIs, which further confirmed the effectiveness of piperazine hybridized coumarin indolylcyanoenone as an antibacterial framework.

## 3. Materials and Methods

### 3.1. Instruments and Chemicals

All commercial chemicals and solvents were used without further purification. TLC analysis was done using pre-coated silica gel plates (Spectrum Separation Materials Co. LTD, Qingdao, China). The melting points of target compounds were determined with WRX-4 melting point instrument. ^1^H NMR and ^13^C NMR spectra were recorded on a 600 MHz Bruker AVANCE III 5 spectrometer (Bruker Instruments LTD, Fällanden, Switzerland) with tetramethylsilane (TMS) as an internal standard. The chemical shifts (δ) were reported in parts per million (ppm), the coupling constants (*J*) were expressed in hertz (Hz) and signals were described as singlet (s), doublet (d), triplet (t) as well as multiplet (m). The high resolution mass spectra (HRMS) were determined by FTICR mass spectrometer (Bruker Instruments LTD, Berlin, Germany) with ESI resource.

### 3.2. Synthesis of Intermediate and Piperazine Hybridized Coumarin Indolylcyanoenones

#### 3.2.1. Synthesis of Intermediate **2**

Intermediate **2** was prepared according to the previously reported method [71].

#### 3.2.2. Synthesis of 3-Oxo-3-(4-(2-oxo-2*H*-chromen-4-yl)piperazin-1-yl)propanenitrile (**3**)

A mixture of cyanoacetic acid (443 mg, 5.21 mmol), *O*-(benzotriazol-1-yl)-*N*,*N*,*N*′,*N*′-tetramethyluronium hexafluorophosphate (HBTU) (2.96 g, 7.82 mmol), and ethyldiisopropylamine (DIPEA) (1.01 g, 7.82 mmol) in *N*,*N*-dimethylformamide (15 mL) was stirred at 25 °C for 30 min. The piperazine coumarin **2** (1.20 g, 5.21 mmol) was added to reaction system, which was further stirred at 40 °C for another 1 h. After the reaction was completed, the reaction solution was poured into cold water to precipitate a solid, which was filtered, and the crude product was purified by silica gel column chromatography (eluent: methanol/dichloromethane = 1/15–20, V/V) to afford white solid compound **3** (856 mg), yield: 55.2%; M.p.: 207.7–208.5 °C; ^1^H NMR (600 MHz, DMSO-*d*_6_) δ 7.76 (d, *J* = 7.9 Hz, 1H, coumarin-5-*H*), 7.61 (t, *J* = 7.6 Hz, 1H, coumarin-7-*H*), 7.39 (d, *J* = 8.2 Hz, 1H, coumarin-8-*H*), 7.35 (t, *J* = 7.5 Hz, 1H, coumarin-6-*H*), 5.73 (s, 1H, coumarin-3-*H*), 4.12 (s, 2H, C*H*_2_), 3.71 (t, *J* = 4.8 Hz, 2H, piperazine-3,3′-2*H*), 3.61 (t, *J* = 4.8 Hz, 2H, piperazine-3,3′-2*H*), 3.30 (t, *J* = 5.0 Hz, 2H, piperazine-2,2′-2*H*), 3.27 (t, *J* = 5.0 Hz, 2H, piperazine-2,2′-2*H*) ppm; HRMS (ESI) calculated for C_16_H_15_N_3_O_3_ [M + H]^+^, 298.1186; found, 298.1184.

#### 3.2.3. Synthesis of (*Z*)-3-(1*H*-indol-3-yl)-2-(4-(2-oxo-2*H*-chromen-4-yl)piperazine-1-carbonyl)acrylonitrile (**5a**)

A mixture of intermediate **3** (50 mg, 0.17 mmol), indole aldehyde **4a** (29 mg, 0.20 mmol), and piperidine (14 mg, 16 mmol) in ethanol (7 mL) was refluxed at 80 °C until completed consumption of intermediate **3,** as monitored by TLC. Afterward, the solvent was evaporated under reduced pressure and the crude product was purified by silica gel column chromatography (eluent: methanol/dichloromethane = 1/10–15, V/V) to generate yellow solid **5a** (55 mg), yield: 77.5%; M.p.: 252.6–253.1 °C; ^1^H NMR (600 MHz, DMSO-*d*_6_) δ 12.25 (s, 1H, N*H*), 8.45 (s, 1H, indole-2-*H*), 8.19 (s, 1H, C=C*H*), 7.95 (d, *J* = 7.8 Hz, 1H, indole-4-*H*), 7.79 (d, *J* = 8.1 Hz, 1H, coumarin-5-*H*), 7.61 (t, *J* = 7.7 Hz, 1H, coumarin-7-*H*), 7.56 (d, *J* = 8.0 Hz, 1H, indole-7-*H*), 7.39 (d, *J* = 8.2 Hz, 1H, coumarin-8-*H*), 7.36 (t, *J* = 7.4 Hz, 1H, coumarin-6-*H*), 7.28 (t, *J* = 7.5 Hz, 1H, indole-6-*H*), 7.23 (t, *J* = 7.4 Hz, 1H, indole-5-*H*), 5.76 (s, 1H, coumarin-3-*H*), 3.88 (t, *J* = 4.8 Hz, 4H, piperazine-3,3′-(C*H*_2_)_2_), 3.38 (t, *J* = 5.1 Hz, 4H, piperazine-2,2′-(C*H*_2_)_2_) ppm; ^13^C NMR (151 MHz, DMSO-*d*_6_) δ 164.30, 161.43, 160.64, 154.11, 144.46, 136.53, 132.31, 130.43, 127.39, 125.85, 124.25, 123.68, 121.93, 119.12, 119.02, 117.69, 116.17, 113.13, 110.37, 97.45, 96.53, 50.75, 44.98 ppm; HRMS (ESI) calculated for C_25_H_20_N_4_O_3_ [M + Na]^+^, 447.1428; found, 447.1426.

#### 3.2.4. Synthesis of (*Z*)-3-(4-chloro-1*H*-indol-3-yl)-2-(4-(2-oxo-2*H*-chromen-4-yl)piperazine-1-carbonyl)acrylonitrile (**5b**)

Compound **5b** was prepared according to the procedure described for compound **5a**, starting from intermediate **3** (40 mg, 0.13 mmol) and 4-chloroindole-3-carboxaldehyde **4b** (24 mg, 0.13 mmol). The pure product **5b** was obtained as a yellow solid (45 mg), yield: 72.6%; M.p.: >300 °C; ^1^H NMR (600 MHz, DMSO-*d*_6_) δ 12.57 (s, 1H, N*H*), 8.79 (s, 1H, indole-2-*H*), 8.62 (s, 1H, C=C*H*), 7.80 (d, *J* = 7.8 Hz, 1H, coumarin-5-*H*), 7.61 (t, *J* = 7.5 Hz, 1H, coumarin-7-*H*), 7.58 (d, *J* = 7.2 Hz, 1H, indole-7-*H*), 7.39 (d, *J* = 8.2 Hz, 1H, coumarin-8-*H*), 7.36 (t, *J* = 7.3 Hz, 1H, coumarin-6-*H*), 7.28–7.24 (m, 2H, indole-5,6-2*H*), 5.75 (s, 1H, coumarin-3-*H*), 3.89 (t, *J* = 5.0 Hz, 4H, piperazine-3,3′-(C*H*_2_)_2_), 3.38 (t, *J* = 5.0 Hz, 4H, piperazine-2,2′-(C*H*_2_)_2_) ppm; ^13^C NMR (151 MHz, DMSO-*d*_6_) δ 164.18, 161.43, 160.63, 154.12, 145.17, 138.23, 132.33, 131.54, 125.87, 125.10, 124.37, 124.26, 123.37, 123.05, 118.82, 117.69, 116.18, 112.83, 110.04, 99.99, 97.48, 50.75, 45.09 ppm; HRMS (ESI) calculated for C_25_H_19_ClN_4_O_3_ [M + H]^+^, 459.1218; found, 459.1218.

#### 3.2.5. Synthesis of (*Z*)-3-(5-chloro-1*H*-indol-3-yl)-2-(4-(2-oxo-2*H*-chromen-4-yl)piperazine-1-carbonyl)acrylonitrile (**5c**)

Compound **5c** was prepared according to the procedure described for compound **5a**, starting from intermediate **3** (45 mg, 0.15 mmol) and 5-chloroindole-3-carboxaldehyde **4c** (27 mg, 0.15 mmol). The pure product **5c** was obtained as a yellow solid (52 mg), yield: 75.4%; M.p.: >300 °C; ^1^H NMR (600 MHz, DMSO-*d*_6_) δ 12.32 (s, 1H, N*H*), 8.48 (s, 1H, indole-2-*H*), 8.16 (s, 1H, C=C*H*), 8.10 (s, 1H, indole-4-*H*), 7.80 (d, *J* = 7.9 Hz, 1H, coumarin-5-*H*), 7.62 (t, *J* = 7.6 Hz, 1H, coumarin-7-*H*), 7.57 (d, *J* = 8.7 Hz, 1H, indole-7-*H*), 7.39 (d, *J* = 8.2 Hz, 1H, coumarin-8-*H*), 7.36 (t, *J* = 7.4 Hz, 1H, coumarin-6-*H*), 7.27 (d, *J* = 8.5 Hz, 1H, indole-6-*H*), 5.76 (s, 1H, coumarin-3-*H*), 3.88 (t, *J* = 4.9 Hz, 4H, piperazine-3,3′-(C*H*_2_)_2_), 3.38 (t, *J* = 4.9 Hz, 4H, piperazine-2,2′-(C*H*_2_)_2_) ppm; ^13^C NMR (151 MHz, DMSO-*d*_6_) δ 164.06, 161.46, 160.65, 154.10, 143.62, 135.00, 132.33, 131.45, 128.60, 126.74, 125.84, 124.27, 123.68, 118.89, 118.78, 117.69, 116.15, 114.69, 110.05, 97.52, 97.39, 50.72, 45.01 ppm; HRMS (ESI) calculated for C_25_H_19_ClN_4_O_3_ [M + H]^+^, 459.1218; found, 459.1221.

#### 3.2.6. Synthesis of (*Z*)-3-(6-fluoro-1*H*-indol-3-yl)-2-(4-(2-oxo-2*H*-chromen-4-yl)piperazine-1-carbonyl)acrylonitrile (**5d**)

Compound **5d** was prepared according to the procedure described for compound **5a**, starting from intermediate **3** (50 mg, 0.17 mmol) and 6-fluoroindole-3-carboxaldehyde **4d** (27 mg, 0.17 mmol). The pure product **5d** was obtained as a yellow solid (48 mg), yield: 64.9%; M.p.: 273.5–274.0 °C; ^1^H NMR (600 MHz, DMSO-*d*_6_) δ 12.27 (s, 1H, N*H*), 8.44 (s, 1H, indole-2-*H*), 8.17 (s, 1H, C=C*H*), 7.98 (dd, *J* = 8.4, 5.3 Hz, 1H, indole-4-*H*), 7.79 (d, *J* = 7.9 Hz, 1H, coumarin-5-*H*), 7.61 (t, *J* = 7.7 Hz, 1H, coumarin-7-*H*), 7.39 (d, *J* = 8.2 Hz, 1H, coumarin-8-*H*), 7.37–7.35 (m, 2H, indole-7-*H*, coumarin-6-*H*), 7.09 (t, *J* = 9.1 Hz, 1H, indole-5-*H*), 5.76 (s, 1H, coumarin-3-*H*), 3.88 (t, *J* = 5.0 Hz, 4H, piperazine-3,3′-(C*H*_2_)_2_), 3.38 (t, *J* = 5.1 Hz, 4H, piperazine-2,2′-(C*H*_2_)_2_) ppm; ^13^C NMR (151 MHz, DMSO-*d*_6_) δ 164.08, 161.43, 160.64, 154.11, 144.09, 136.54, 136.46, 132.31, 130.90, 125.84, 124.25, 124.02, 120.50, 120.44, 118.85, 117.69, 116.16, 110.39, 110.25, 99.36, 99.18, 97.47, 50.74, 44.98 ppm; HRMS (ESI) calculated for C_25_H_19_FN_4_O_3_ [M + Na]^+^, 465.1333; found, 465.1334.

#### 3.2.7. Synthesis of (*Z*)-3-(6-bromo-1*H*-indol-3-yl)-2-(4-(2-oxo-2*H*-chromen-4-yl)piperazine-1-carbonyl)acrylonitrile (**5e**)

Compound **5e** was prepared according to the procedure described for compound **5a**, starting from intermediate **3** (50 mg, 0.17 mmol) and 6-bromoindole-3-carboxaldehyde **4e** (38 mg, 0.17 mmol). The pure product **5e** was obtained as a yellow solid (60 mg), yield: 70.9%; M.p.: 282.4–282.9 °C; ^1^H NMR (600 MHz, DMSO-*d*_6_) δ 12.30 (s, 1H, N*H*), 8.45 (s, 1H, indole-2-*H*), 8.16 (s, 1H, C=C*H*), 7.93 (d, *J* = 8.5 Hz, 1H, indole-4-*H*), 7.79 (d, *J* = 8.0 Hz, 1H, coumarin-5-*H*), 7.75 (s, 1H, indole-7-*H*), 7.61 (t, *J* = 7.7 Hz, 1H, coumarin-7-*H*), 7.39 (d, *J* = 8.3 Hz, 1H, coumarin-8-*H*), 7.37–7.35 (m, 2H, coumarin-6-*H*, indole-5-*H*), 5.76 (s, 1H, coumarin-3-*H*), 3.88 (t, *J* = 5.0 Hz, 4H, piperazine-3,3′-(C*H*_2_)_2_), 3.38 (t, *J* = 5.1 Hz, 4H, piperazine-2,2′-(C*H*_2_)_2_) ppm; ^13^C NMR (151 MHz, DMSO-*d*_6_) δ 163.99, 161.43, 160.64, 154.11, 143.82, 137.34, 132.32, 131.03, 126.41, 125.83, 124.69, 124.25, 120.99, 118.78, 117.69, 116.24, 116.16, 115.75, 110.39, 97.79, 97.47, 50.73, 44.98 ppm; HRMS (ESI) calculated for C_25_H_19_BrN_4_O_3_ [M + H]^+^, 503.0713; found, 503.0713.

#### 3.2.8. Synthesis of (*Z*)-3-(6-methyl-1*H*-indol-3-yl)-2-(4-(2-oxo-2*H*-chromen-4-yl)piperazine-1-carbonyl)acrylonitrile (**5f**)

Compound **5f** was prepared according to the procedure described for compound **5a**, starting from intermediate **3** (45 mg, 0.15 mmol) and 6-methyl-1*H*-indole-3-carbaldehyde **4f** (24 mg, 0.15 mmol). The pure product **5f** was obtained as a yellow solid (52 mg), yield: 78.8%; M.p.: 242.6–243.1 °C; ^1^H NMR (600 MHz, DMSO-*d*_6_) δ 12.17 (s, 1H, N*H*), 8.38 (s, 1H, indole-2-*H*), 8.15 (s, 1H, C=C*H*), 7.83 (d, *J* = 8.1 Hz, 1H, indole-4-*H*), 7.79 (d, *J* = 8.0 Hz, 1H, coumarin-5-*H*), 7.62 (t, *J* = 7.7 Hz, 1H, coumarin-7-*H*), 7.40 (d, *J* = 8.2 Hz, 1H, coumarin-8-*H*), 7.37 (t, *J* = 7.7 Hz, 1H, coumarin-6-*H*), 7.34 (s, 1H, indole-7-*H*), 7.06 (d, *J* = 8.1 Hz, 1H, indole-5-*H*), 5.76 (s, 1H, coumarin-3-*H*), 3.88 (t, *J* = 4.8 Hz, 4H, piperazine-3,3′-(C*H*_2_)_2_), 3.37 (t, *J* = 5.1 Hz, 4H, piperazine-2,2′-(C*H*_2_)_2_), 2.43 (s, 3H, indole-6-C*H*_3_) ppm; ^13^C NMR (151 MHz, DMSO-*d*_6_) δ 164.37, 161.43, 160.64, 154.11, 144.69, 136.93, 133.09, 132.31, 130.07, 125.85, 125.24, 124.25, 123.67, 119.18, 118.79, 117.69, 116.16, 112.81, 110.36, 97.44, 96.17, 50.75, 44.97, 21.70 ppm; HRMS (ESI) calculated for C_26_H_22_N_4_O_3_ [M + H]^+^, 439.1765; found, 439.1765.

#### 3.2.9. Synthesis of (*Z*)-3-(7-methyl-1*H*-indol-3-yl)-2-(4-(2-oxo-2*H*-chromen-4-yl)piperazine-1-carbonyl)acrylonitrile (**5g**)

Compound **5g** was prepared according to the procedure described for compound **5a**, starting from intermediate **3** (45 mg, 0.15 mmol) and 7-methyl-1*H*-indole-3-carbaldehyde **4g** (24 mg, 0.15 mmol). The pure product **5g** was obtained as a yellow solid (42 mg), yield: 63.6%; M.p.: 291.1–291.6 °C; ^1^H NMR (600 MHz, DMSO-*d*_6_) δ 12.33 (s, 1H, N*H*), 8.40 (s, 1H, indole-2-*H*), 8.17 (s, 1H, C=C*H*), 7.80 (d, *J* = 7.9 Hz, 1H, indole-4-*H*), 7.77 (d, *J* = 7.9 Hz, 1H, coumarin-5-*H*), 7.62 (t, *J* = 7.7 Hz, 1H, coumarin-7-*H*), 7.39 (d, *J* = 8.2 Hz, 1H, coumarin-8-*H*), 7.36 (t, *J* = 7.6 Hz, 1H, coumarin-6-*H*), 7.14 (t, *J* = 7.5 Hz, 1H, indole-5-*H*), 7.08 (d, *J* = 7.0 Hz, 1H, indole-6-*H*), 5.76 (s, 1H, coumarin-3-*H*), 3.88 (t, *J* = 4.9 Hz, 4H, piperazine-3,3′-(C*H*_2_)_2_), 3.38 (t, *J* = 5.0 Hz, 4H, piperazine-2,2′-(C*H*_2_)_2_), 2.52 (s, 3H, CH_3_) ppm; ^13^C NMR (151 MHz, DMSO-*d*_6_) δ 164.27, 161.43, 160.65, 154.11, 144.58, 135.97, 132.32, 129.84, 127.24, 125.85, 124.28, 124.25, 122.40, 122.15, 119.15, 117.69, 116.53, 116.17, 110.78, 97.44, 96.62, 50.74, 44.98, 17.01 ppm; HRMS (ESI) calculated for C_26_H_22_N_4_O_3_ [M + H]^+^, 439.1765; found, 439.1766.

#### 3.2.10. Synthesis of (*Z*)-3-(1-ethyl-1*H*-indol-3-yl)-2-(4-(2-oxo-2*H*-chromen-4-yl)piperazine-1-carbonyl)acrylonitrile (**7a**)

Compound **7a** was prepared according to the procedure described for compound **5a**, starting from intermediate **3** (50 mg, 0.17 mmol) and **6a** (29 mg, 0.17 mmol). The pure product **7a** was obtained as a yellow solid (48 mg), yield: 63.2%; M.p.: 179.1–179.9 °C; ^1^H NMR (600 MHz, DMSO-*d*_6_) δ 8.48 (s, 1H, indole-2-*H*), 8.16 (s, 1H, C=C*H*), 7.98 (d, *J* = 7.6 Hz, 1H, indole-4-*H*), 7.80 (d, *J* = 8.0 Hz, 1H, coumarin-5-*H*), 7.66 (d, *J* = 8.2 Hz, 1H, indole-7-*H*), 7.62 (t, *J* = 7.7 Hz, 1H, coumarin-7-*H*), 7.40 (d, *J* = 8.3 Hz, 1H, coumarin-8-*H*), 7.36 (t, *J* = 7.6 Hz, 1H, coumarin-6-*H*), 7.33 (t, *J* = 7.6 Hz, 1H, indole-6-*H*), 7.27 (t, *J* = 7.5 Hz, 1H, indole-5-*H*), 5.77 (s, 1H, coumarin-3-*H*), 4.40 (q, *J* = 7.2 Hz, 2H, C*H*_2_CH_3_), 3.88 (t, *J* = 4.9 Hz, 4H, piperazine-3,3′-(C*H*_2_)_2_), 3.38 (t, *J* = 5.0 Hz, 4H, piperazine-2,2′-(C*H*_2_)_2_), 1.43 (t, *J* = 7.2 Hz, 3H, CH_2_C*H*_3_) ppm; ^13^C NMR (151 MHz, DMSO-*d*_6_) δ 164.29, 161.43, 160.65, 154.11, 143.81, 136.16, 132.32, 132.20, 128.05, 125.85, 124.26, 123.78, 122.26, 119.35, 119.02, 117.70, 116.16, 111.56, 109.62, 97.45, 96.45, 50.74, 44.97, 41.99, 15.56 ppm; HRMS (ESI) calculated for C_27_H_24_N_4_O_3_ [M + H]^+^, 453.1921; found, 453.1920.

#### 3.2.11. Synthesis of (*Z*)-3-(1-butyl-1*H*-indol-3-yl)-2-(4-(2-oxo-2*H*-chromen-4-yl)piperazine-1-carbonyl)acrylonitrile (**7b**)

Compound **7b** was prepared according to the procedure described for compound **5a**, starting from intermediate **3** (50 mg, 0.17 mmol) and **6b** (34 mg, 0.17 mmol). The pure product **7b** was obtained as a yellow solid (50 mg), yield: 61.7%; M.p.: 185.4–186.0 °C; ^1^H NMR (600 MHz, DMSO-*d*_6_) δ 8.45 (s, 1H, indole-2-*H*), 8.16 (s, 1H, C=C*H*), 7.97 (d, *J* = 7.9 Hz, 1H, indole-4-*H*), 7.80 (d, *J* = 7.9 Hz, 1H, coumarin-5-*H*), 7.66 (d, *J* = 8.2 Hz, 1H, indole-7-*H*), 7.62 (t, *J* = 7.7 Hz, 1H, coumarin-7-*H*), 7.40 (d, *J* = 8.2 Hz, 1H, coumarin-8-*H*), 7.36 (t, *J* = 7.6 Hz, 1H, coumarin-6-*H*), 7.33 (t, *J* = 7.6 Hz, 1H, indole-6-*H*), 7.27 (t, *J* = 7.5 Hz, 1H, indole-5-*H*), 5.76 (s, 1H, coumarin-3-*H*), 4.36 (t, *J* = 7.0 Hz, 2H, C*H*_2_(CH_2_)_2_CH_3_), 3.88 (t, *J* = 4.9 Hz, 4H, piperazine-3,3′-(C*H*_2_)_2_), 3.38 (t, *J* = 5.0 Hz, 4H, piperazine-2,2′-(C*H*_2_)_2_), 1.79 (p, *J* = 7.2 Hz, 2H, C*H*_2_CH_2_CH_3_), 1.29 (dt, *J* = 14.7, 7.4 Hz, 2H, C*H*_2_CH_3_), 0.91 (t, *J* = 7.4 Hz, 3H CH_2_C*H*_3_) ppm; ^13^C NMR (151 MHz, DMSO-*d*_6_) δ 164.27, 161.43, 160.65, 154.11, 143.77, 136.45, 132.74, 132.33, 127.96, 125.85, 124.26, 123.78, 122.22, 119.33, 119.00, 117.70, 116.16, 111.64, 109.54, 97.45, 96.55, 50.74, 46.73, 44.97, 32.05, 19.85, 13.90 ppm; HRMS (ESI) calculated for C_29_H_28_N_4_O_3_ [M + H]^+^, 481.2234; found, 481.2232.

#### 3.2.12. Synthesis of (*Z*)-3-(1-hexyl-1*H*-indol-3-yl)-2-(4-(2-oxo-2*H*-chromen-4-yl)piperazine-1-carbonyl)acrylonitrile (**7c**)

Compound **7c** was prepared according to the procedure described for compound **5a**, starting from intermediate **3** (40 mg, 0.13 mmol) and **6c** (31 mg, 0.13 mmol). The pure product **7c** was obtained as a yellow solid (45 mg), yield: 66.2%; M.p.: 152.7–153.0 °C; ^1^H NMR (600 MHz, DMSO-*d*_6_) δ 8.45 (s, 1H, indole-2-*H*), 8.16 (s, 1H, C=C*H*), 7.97 (d, *J* = 7.9 Hz, 1H, indole-4-*H*), 7.80 (d, *J* = 7.9 Hz, 1H, coumarin-5-*H*), 7.66 (d, *J* = 8.2 Hz, 1H, indole-7-*H*), 7.62 (t, *J* = 7.9 Hz, 1H, coumarin-7-*H*), 7.40 (d, *J* = 8.2 Hz, 1H, coumarin-8-*H*), 7.36 (t, *J* = 7.6 Hz, 1H, coumarin-6-*H*), 7.33 (t, *J* = 7.6 Hz, 1H, indole-6-*H*), 7.27 (t, *J* = 7.5 Hz, 1H, indole-5-*H*), 5.77 (s, 1H, coumarin-3-*H*), 4.36 (t, *J* = 7.0 Hz, 2H, C*H*_2_(CH_2_)_4_CH_3_), 3.88 (t, *J* = 5.0 Hz, 4H, piperazine-3,3′-(C*H*_2_)_2_), 3.38 (t, *J* = 5.0 Hz, 4H, piperazine-2,2′-(C*H*_2_)_2_), 1.80 (p, *J* = 7.0 Hz, 2H, C*H*_2_(CH_2_)_3_CH_3_), 1.27 (dq, *J* = 13.4, 6.8, 5.5 Hz, 6H, (C*H*_2_)_3_CH_3_), 0.84 (t, *J* = 6.7 Hz, 3H, CH_2_C*H*_3_) ppm; ^13^C NMR (151 MHz, DMSO-*d*_6_) δ 164.27, 161.43, 160.64, 154.11, 143.79, 136.44, 132.75, 132.32, 127.96, 125.85, 124.25, 123.78, 122.21, 119.33, 119.00, 117.69, 116.16, 111.63, 109.53, 97.45, 96.55, 50.74, 46.96, 44.97, 31.15, 29.89, 26.19, 22.42, 14.25 ppm; HRMS (ESI) calculated for C_31_H_32_N_4_O_3_ [M + H]^+^, 509.2547; found, 509.2547.

#### 3.2.13. Synthesis of (*Z*)-3-(1-(prop-2-yn-1-yl)-1*H*-indol-3-yl)-2-(4-(2-oxo-2*H*-chromen-4-yl)piperazine-1-carbonyl)acrylonitrile (**7d**)

Compound **7d** was prepared according to the procedure described for compound **5a**, starting from intermediate **3** (50 mg, 0.17 mmol) and **6d** (31 mg, 0.17 mmol). The pure product **7d** was obtained as a yellow solid (65 mg), yield: 83.6%; M.p.: 210.6–211.0 °C; ^1^H NMR (600 MHz, DMSO-*d*_6_) δ 8.56 (s, 1H, indole-2-*H*), 8.17 (s, 1H, C=C*H*), 8.00 (d, *J* = 7.7 Hz, 1H, indole-4-*H*), 7.80 (d, *J* = 7.6 Hz, 1H, coumarin-5-*H*), 7.68 (d, *J* = 8.0 Hz, 1H, indole-7-*H*), 7.62 (t, *J* = 7.3 Hz, 1H, coumarin-7-*H*), 7.40 (d, *J* = 8.9 Hz, 1H, coumarin-8-*H*), 7.39–7.36 (m, 2H, coumarin-6-*H*, indole-6-*H*), 7.30 (t, *J* = 7.1 Hz, 1H, indole-5-*H*), 5.77 (s, 1H, coumarin-3-*H*), 5.35 (s, 2H, C*H*_2_C≡CH), 3.89 (t, *J* = 5.0 Hz, 4H, piperazine-3,3′-(C*H*_2_)_2_), 3.58 (s, 1H, C≡C*H*), 3.38 (t, *J* = 5.0 Hz, 4H, piperazine-2,2′-(C*H*_2_)_2_) ppm; ^13^C NMR (151 MHz, DMSO-*d*_6_) δ 164.02, 161.43, 160.64, 154.11, 143.40, 136.07, 132.32, 131.91, 128.05, 125.84, 124.25, 124.00, 122.52, 119.40, 118.71, 117.70, 116.16, 111.72, 110.09, 97.66, 97.46, 78.43, 77.32, 50.73, 44.95, 36.64 ppm; HRMS (ESI) calculated for C_28_H_22_N_4_O_3_ [M + H]^+^, 463.1765; found, 463.1764.

#### 3.2.14. Synthesis of (*Z*)-3-(1-allyl-1*H*-indol-3-yl)-2-(4-(2-oxo-2*H*-chromen-4-yl)piperazine-1-carbonyl)acrylonitrile (**7e**)

Compound **7e** was prepared according to the procedure described for compound **5a**, starting from intermediate **3** (50 mg, 0.17 mmol) and **6e** (31 mg, 0.17 mmol). The pure product **7e** was obtained as a yellow solid (55 mg), yield: 70.5%; M.p.: 178.2–178.6 °C; ^1^H NMR (600 MHz, DMSO-*d*_6_) δ 8.45 (s, 1H, indole-2-*H*), 8.17 (s, 1H, C=C*H*), 7.99 (d, *J* = 7.9 Hz, 1H, indole-4-*H*), 7.80 (d, *J* = 7.9 Hz, 1H, coumarin-5-*H*), 7.63–7.60 (m, 2H, coumarin-7-*H*, indole-7-*H*), 7.40 (d, *J* = 8.3 Hz, 1H, coumarin-8-*H*), 7.36 (t, *J* = 7.6 Hz, 1H, coumarin-6-*H*), 7.33 (t, *J* = 7.5 Hz, 1H, indole-6-*H*), 7.27 (t, *J* = 7.4 Hz, 1H, indole-5-*H*), 6.06 (ddt, *J* = 16.1, 10.6, 5.5 Hz, 1H, CH_2_C*H*=CH_2_), 5.77 (s, 1H, coumarin-3-*H*), 5.25 (d, *J* = 10.2 Hz, 1H, CH_2_CH=C*H*), 5.15 (d, *J* = 17.1 Hz, 1H, CH_2_CH=C*H*), 5.04 (d, *J* = 5.0 Hz, 2H, C*H*_2_CH=CH_2_), 3.88 (t, *J* = 5.0 Hz, 4H, piperazine-3,3′-(C*H*_2_)_2_), 3.38 (t, *J* = 5.0 Hz, 4H, piperazine-2,2′-(C*H*_2_)_2_) ppm; ^13^C NMR (151 MHz, DMSO-*d*_6_) δ 164.18, 161.43, 160.64, 154.11, 143.64, 136.46, 133.76, 132.62, 132.33, 128.01, 125.85, 124.26, 123.84, 122.31, 119.31, 118.89, 118.41, 117.70, 116.16, 111.85, 109.81, 97.45, 96.93, 50.74, 49.37, 44.95 ppm; HRMS (ESI) calculated for C_28_H_24_N_4_O_3_ [M + H]^+^, 465.1921; found, 465.1923.

#### 3.2.15. Synthesis of (*Z*)-3-(1-(3-methylbut-2-en-1-yl)-1*H*-indol-3-yl)-2-(4-(2-oxo-2*H*-chromen-4-yl)piperazine-1-carbonyl)acrylonitrile (**7f**)

Compound **7f** was prepared according to the procedure described for compound **5a**, starting from intermediate **3** (75 mg, 0.25 mmol) and **6f** (54 mg, 0.25 mmol). The pure product **7f** was obtained as a yellow solid (105 mg), yield: 84.7%; M.p.: 187.2–187.8 °C; ^1^H NMR (600 MHz, DMSO-*d*_6_) δ 8.45 (s, 1H, indole-2-*H*), 8.16 (s, 1H, C=C*H*), 7.97 (d, *J* = 7.9 Hz, 1H, indole-4-*H*), 7.79 (d, *J* = 8.0 Hz, 1H, coumarin-5-*H*), 7.63–7.58 (m, 2H, coumarin-7-*H*, indole-7-*H*), 7.40 (d, *J* = 8.3 Hz, 1H, coumarin-8-*H*), 7.36 (t, *J* = 7.7 Hz, 1H, coumarin-6-*H*), 7.33 (t, *J* = 7.7 Hz, 1H, indole-6-*H*), 7.27 (t, *J* = 7.5 Hz, 1H, indole-5-*H*), 5.76 (s, 1H, coumarin-3-*H*), 5.41 (t, *J* = 6.4 Hz, 1H, (CH_3_)_2_C=C*H*), 4.95 (d, *J* = 6.9 Hz, 2H, C*H*_2_CH=C(CH_3_)_2_), 3.88 (t, *J* = 5.0 Hz, 4H, piperazine-3,3′-(C*H*_2_)_2_), 3.38 (t, *J* = 5.0 Hz, 4H, piperazine-2,2′-(C*H*_2_)_2_), 1.85 (s, 3H, CH=CC*H*_3_), 1.75 (s, 3H, CH=CC*H*_3_) ppm; ^13^C NMR (151 MHz, DMSO-*d*_6_) δ 164.25, 161.43, 160.64, 154.11, 143.76, 138.22, 136.37, 132.32, 132.25, 128.13, 125.84, 124.25, 123.77, 122.29, 119.32, 119.27, 119.05, 117.70, 116.16, 111.71, 109.62, 97.45, 96.46, 50.74, 44.95, 44.88, 25.76, 18.41 ppm; HRMS (ESI) calculated for C_30_H_28_N_4_O_3_ [M + Na]^+^, 515.2054; found, 515.2055.

#### 3.2.16. Synthesis of (*Z*)-3-(1-(cyclopropylmethyl)-1*H*-indol-3-yl)-2-(4-(2-oxo-2*H*-chromen-4-yl)piperazine-1-carbonyl)acrylonitrile (**9a**)

Compound **9a** was prepared according to the procedure described for compound **5a**, starting from intermediate **3** (50 mg, 0.17 mmol) and **8a** (34 mg, 0.17 mmol). The pure product **9a** was obtained as a yellow solid (61 mg), yield: 76.3%; M.p.: 206.9–207.5 °C; ^1^H NMR (600 MHz, DMSO-*d*_6_) δ 8.58 (s, 1H, indole-2-*H*), 8.17 (s, 1H, C=C*H*), 7.98 (d, *J* = 7.9 Hz, 1H, indole-4-*H*), 7.80 (d, *J* = 8.0 Hz, 1H, coumarin-5-*H*), 7.70 (d, *J* = 8.2 Hz, 1H, indole-7-*H*), 7.62 (t, *J* = 7.8 Hz, 1H, coumarin-7-*H*), 7.40 (d, *J* = 8.2 Hz, 1H, coumarin-8-*H*), 7.36 (t, *J* = 7.6 Hz, 1H, coumarin-6-*H*), 7.33 (t, *J* = 7.7 Hz, 1H, indole-6-*H*), 7.27 (t, *J* = 7.4 Hz, 1H, indole-5-*H*), 5.76 (s, 1H, coumarin-3-*H*), 4.24 (d, *J* = 7.1 Hz, 2H, C*H*_2_CH), 3.88 (t, *J* = 5.1 Hz, 4H, piperazine-3,3′-(C*H*_2_)_2_), 3.38 (t, *J* = 5.1 Hz, 4H, piperazine-2,2′-(C*H*_2_)_2_), 1.36–1.30 (m, 1H, CH_2_C*H*), 0.61–0.56 (m, 2H, cyclopropyl-2,2′-2*H*), 0.47–0.44 (m, 2H, cyclopropyl-2,2′-2*H*) ppm; ^13^C NMR (151 MHz, DMSO-*d*_6_) δ 164.29, 161.44, 160.65, 154.11, 143.82, 136.60, 132.45, 132.33, 127.95, 125.85, 124.26, 123.76, 122.25, 119.25, 119.04, 117.70, 116.16, 111.73, 109.59, 97.44, 96.50, 50.95, 50.74, 44.95, 11.56, 4.28 ppm; HRMS (ESI) calculated for C_29_H_26_N_4_O_3_ [M + H]^+^, 479.2078; found, 479.2077.

#### 3.2.17. Synthesis of (*Z*)-3-(1-(cyclopentylmethyl)-1*H*-indol-3-yl)-2-(4-(2-oxo-2*H*-chromen-4-yl)piperazine-1-carbonyl)acrylonitrile (**9b**)

Compound **9b** was prepared according to the procedure described for compound **5a**, starting from intermediate **3** (45 mg, 0.15 mmol) and **8b** (34 mg, 0.15 mmol). The pure product **9b** was obtained as a yellow solid (52 mg), yield: 67.5%; M.p.: 206.1–206.5 °C; ^1^H NMR (600 MHz, DMSO-*d*_6_) δ 8.47 (s, 1H, indole-2-*H*), 8.17 (s, 1H, C=C*H*), 7.97 (d, *J* = 7.4 Hz, 1H, indole-4-*H*), 7.80 (d, *J* = 7.4 Hz, 1H, coumarin-5-*H*), 7.69 (d, *J* = 7.7 Hz, 1H, indole-7-*H*), 7.62 (t, *J* = 7.0 Hz, 1H, coumarin-7-*H*), 7.40 (d, *J* = 8.0 Hz, 1H, coumarin-8-*H*), 7.36 (t, *J* = 7.3 Hz, 1H, coumarin-6-*H*), 7.33 (t, *J* = 7.7 Hz, 1H, indole-6-*H*), 7.26 (t, *J* = 6.7 Hz, 1H, indole-5-*H*), 5.76 (s, 1H, coumarin-3-*H*), 4.29 (d, *J* = 6.9 Hz, 2H, C*H*_2_CH), 3.88 (t, *J* = 5.0 Hz, 4H, piperazine-3,3′-(C*H*_2_)_2_), 3.38 (t, *J* = 5.0 Hz, 4H, piperazine-2,2′-(C*H*_2_)_2_), 2.40 (p, *J* = 7.6 Hz, 1H, CH_2_C*H*), 1.64 (td, *J* = 14.1, 6.3 Hz, 4H, cyclopentyl-2,2′,3,3′-4*H*), 1.54–1.48 (m, 2H, cyclopentyl-2,2′-2*H*), 1.32–1.25 (m, 2H, cyclopentyl-3,3′-2*H*) ppm; ^13^C NMR (151 MHz, DMSO-*d*_6_) δ 164.26, 161.43, 160.64, 154.11, 143.79, 136.67, 132.83, 132.32, 127.88, 125.84, 124.25, 123.74, 122.19, 119.29, 119.01, 117.70, 116.16, 111.75, 109.52, 97.44, 96.61, 51.40, 50.75, 44.99, 30.30, 24.92 ppm; HRMS (ESI) calculated for C_31_H_30_N_4_O_3_ [M + H]^+^, 507.2391; found, 507.2393.

#### 3.2.18. Synthesis of (*Z*)-3-(1-(cyclohexylmethyl)-1*H*-indol-3-yl)-2-(4-(2-oxo-2*H*-chromen-4-yl)piperazine-1-carbonyl)acrylonitrile (**9c**)

Compound **9c** was prepared according to the procedure described for compound **5a**, starting from intermediate **3** (75 mg, 0.25 mmol) and **8c** (61 mg, 0.25 mmol). The pure product **9c** was obtained as a yellow solid (103 mg), yield: 78.6%; M.p.: 131.4–132.3 °C; ^1^H NMR (600 MHz, DMSO-*d*_6_) δ 8.40 (s, 1H, indole-2-*H*), 8.16 (s, 1H, C=C*H*), 7.97 (d, *J* = 7.9 Hz, 1H, indole-4-*H*), 7.80 (d, *J* = 7.9 Hz, 1H, coumarin-5-*H*), 7.67 (d, *J* = 8.2 Hz, 1H, indole-7-*H*), 7.62 (t, *J* = 7.7 Hz, 1H, coumarin-7-*H*), 7.40 (d, *J* = 8.2 Hz, 1H, coumarin-8-*H*), 7.36 (t, *J* = 7.6 Hz, 1H, coumarin-6-*H*), 7.32 (t, *J* = 7.6 Hz, 1H, indole-6-*H*), 7.26 (t, *J* = 7.5 Hz, 1H, indole-5-*H*), 5.76 (s, 1H, coumarin-3-*H*), 4.21 (d, *J* = 7.2 Hz, 2H, CHC*H*_2_), 3.88 (t, *J* = 5.0 Hz, 4H, piperazine-3,3′-(C*H*_2_)_2_), 3.38 (t, *J* = 5.0 Hz, 4H, piperazine-2,2′-(C*H*_2_)_2_), 1.83 (ddh, *J* = 11.3, 7.7, 3.7 Hz, 1H, CH_2_C*H*), 1.70–1.63 (m, 2H, cyclohexyl-2,2′-2*H*), 1.62–1.57 (m, 1H, cyclohexyl-4-*H*), 1.53 (d, *J* = 12.1 Hz, 2H, cyclohexyl-3,3′-2*H*), 1.20–1.11 (m, 3H, cyclohexyl-3,3′,4-3*H*), 1.03 (q, *J* = 12.6, 12.1 Hz, 2H, cyclohexyl-2,2′-2*H*) ppm; ^13^C NMR (151 MHz, DMSO-*d*_6_) δ 164.25, 161.43, 160.64, 154.11, 143.74, 136.85, 133.20, 132.32, 127.85, 125.84, 124.25, 123.75, 122.16, 119.26, 118.99, 117.70, 116.16, 111.89, 109.42, 97.44, 96.64, 65.34, 52.86, 50.75, 44.94, 38.57, 30.46, 26.26, 25.59, 15.61 ppm; HRMS (ESI) calculated for C_32_H_32_N_4_O_3_ [M + H]^+^, 521.2547; found, 521.2547.

#### 3.2.19. Synthesis of (*Z*)-3-(1-(2-fluorobenzyl)-1*H*-indol-3-yl)-2-(4-(2-oxo-2*H*-chromen-4-yl)piperazine-1-carbonyl)acrylonitrile (**11a**)

Compound **11a** was prepared according to the procedure described for compound **5a**, starting from intermediate **3** (40 mg, 0.13 mmol) and **10a** (34 mg, 0.13 mmol). The pure product **11a** was obtained as a yellow solid (54 mg), yield: 75.0%; M.p.: 236.9–237.3 °C; ^1^H NMR (600 MHz, DMSO-*d*_6_) δ 8.56 (s, 1H, indole-2-*H*), 8.17 (s, 1H, C=C*H*), 7.99 (d, *J* = 7.8 Hz, 1H, indole-4-*H*), 7.79 (d, *J* = 7.7 Hz, 1H, coumarin-5-*H*), 7.66 (d, *J* = 8.1 Hz, 1H, indole-7-*H*), 7.62 (t, *J* = 7.6 Hz, 1H, coumarin-7-*H*), 7.41–7.38 (m, 2H, coumarin-8-*H*, benzene-4-*H*), 7.35 (t, *J* = 7.6 Hz, 1H, coumarin-6-*H*), 7.33–7.29 (m, 2H, indole-6-*H*, benzene-3-*H*), 7.28–7.24 (m, 2H, indole-5-*H*, benzene-6-*H*), 7.18 (t, *J* = 7.5 Hz, 1H, benzene-5-*H*), 5.76 (s, 1H, coumarin-3-*H*), 5.69 (s, 2H, C*H*_2_), 3.88 (t, *J* = 5.0 Hz, 4H, piperazine-3,3′-(C*H*_2_)_2_), 3.38 (t, *J* = 5.0 Hz, 4H, piperazine-2,2′-(C*H*_2_)_2_) ppm; ^13^C NMR (151 MHz, DMSO-*d*_6_) δ 164.08, 161.54, 161.43, 160.64, 159.91, 154.11, 143.43, 136.40, 132.83, 132.32, 130.87, 130.81, 130.54, 130.52, 127.98, 125.84, 125.31, 125.28, 124.25, 124.01, 122.38, 119.40, 118.74, 117.70, 116.23, 116.16, 116.09, 111.67, 110.06, 97.74, 97.45, 50.73, 44.93, 44.75 ppm; HRMS (ESI) calculated for C_32_H_25_FN_4_O_3_ [M + H]^+^, 533.1983; found, 533.1983.

#### 3.2.20. Synthesis of (*Z*)-3-(1-(2-chlorobenzyl)-1*H*-indol-3-yl)-2-(4-(2-oxo-2*H*-chromen-4-yl)piperazine-1-carbonyl)acrylonitrile (**11b**)

Compound **11b** was prepared according to the procedure described for compound **5a**, starting from intermediate **3** (40 mg, 0.13 mmol) and **10b** (36 mg, 0.13 mmol). The pure product **11b** was obtained as a yellow solid (47 mg), yield: 63.5%; M.p.: 210.8–211.3 °C; ^1^H NMR (600 MHz, DMSO-*d*_6_) δ 8.53 (s, 1H, indole-2-*H*), 8.18 (s, 1H, C=C*H*), 8.01 (d, *J* = 7.7 Hz, 1H, indole-4-*H*), 7.79 (d, *J* = 7.9 Hz, 1H, coumarin-5-*H*), 7.62–7.58 (m, 2H, indole-7-*H*, coumarin-7-*H*), 7.55 (d, *J* = 7.8 Hz, 1H, benzene-3-*H*), 7.39 (d, *J* = 8.3 Hz, 1H, coumarin-8-*H*), 7.38–7.34 (m, 2H, coumarin-6-*H*, benzene-4-*H*), 7.32–7.27 (m, 3H, indole-5-*H*, indole-6-*H*, benzene-5-*H*), 7.04 (d, *J* = 7.5 Hz, 1H, benzene-6-*H*), 5.76 (s, 1H, coumarin-3-*H*), 5.72 (s, 2H, C*H*_2_), 3.88 (t, *J* = 5.0 Hz, 4H, piperazine-3,3′-(C*H*_2_)_2_), 3.37 (t, *J* = 5.0 Hz, 4H, piperazine-2,2′-(C*H*_2_)_2_) ppm; ^13^C NMR (151 MHz, DMSO-*d*_6_) δ 164.06, 161.43, 160.64, 154.11, 143.44, 136.57, 134.30, 132.97, 132.92, 132.32, 130.34, 130.24, 129.91, 128.18, 127.97, 125.84, 124.25, 124.08, 122.44, 119.45, 118.71, 117.70, 116.16, 111.73, 110.10, 97.53, 97.45, 50.73, 48.37, 44.99 ppm; HRMS (ESI) calculated for C_32_H_25_ClN_4_O_3_ [M + H]^+^, 549.1688; found, 549.1686.

#### 3.2.21. Synthesis of (*Z*)-3-(1-(3-fluorobenzyl)-1*H*-indol-3-yl)-2-(4-(2-oxo-2*H*-chromen-4-yl)piperazine-1-carbonyl)acrylonitrile (**11c**)

Compound **11c** was prepared according to the procedure described for compound **5a**, starting from intermediate **3** (35 mg, 0.12 mmol) and **10c** (30 mg, 0.12 mmol). The pure product **11c** was obtained as a yellow solid (43 mg), yield: 68.3%; M.p.: 192.5–192.9 °C; ^1^H NMR (600 MHz, DMSO-*d*_6_) δ 8.60 (s, 1H, indole-2-*H*), 8.18 (s, 1H, C=C*H*), 8.00 (d, *J* = 7.7 Hz, 1H, indole-4-*H*), 7.80 (d, *J* = 7.9 Hz, 1H, coumarin-5-*H*), 7.63–7.61 (m, 2H, indole-7-*H*, coumarin-7-*H*), 7.41–7.38 (m, 2H, benzene-5-*H*, coumarin-8-*H*), 7.36 (t, *J* = 7.6 Hz, 1H, coumarin-6-*H*), 7.30 (t, *J* = 7.6 Hz, 1H, indole-6-*H*), 7.27 (t, *J* = 7.4 Hz, 1H, indole-5-*H*), 7.15–7.13 (m, 2H, benzene-4,6-2*H*), 7.10 (d, *J* = 7.7 Hz, 1H, benzene-2-*H*), 5.77 (s, 1H, coumarin-3-*H*), 5.68 (s, 2H, C*H*_2_), 3.89 (t, *J* = 5.0 Hz, 4H, piperazine-3,3′-(C*H*_2_)_2_), 3.38 (t, *J* = 5.0 Hz, 4H, piperazine-2,2′-(C*H*_2_)_2_) ppm; ^13^C NMR (151 MHz, DMSO-*d*_6_) δ 164.11, 163.54, 161.92, 161.44, 160.65, 154.11, 143.53, 140.17, 136.43, 132.91, 132.33, 131.33, 131.28, 128.07, 125.84, 124.26, 124.02, 123.77, 123.75, 122.39, 119.45, 118.75, 117.70, 116.16, 115.18, 115.05, 114.70, 114.56, 111.87, 110.12, 97.53, 97.46, 50.74, 49.80, 44.96 ppm; HRMS (ESI) calculated for C_32_H_25_FN_4_O_3_ [M + H]^+^, 533.1983; found, 533.1983.

#### 3.2.22. Synthesis of (*Z*)-3-(1-(3-chlorobenzyl)-1*H*-indol-3-yl)-2-(4-(2-oxo-2*H*-chromen-4-yl)piperazine-1-carbonyl)acrylonitrile (**11d**)

Compound **11d** was prepared according to the procedure described for compound **5a**, starting from intermediate **3** (35 mg, 0.12 mmol) and **10d** (32 mg, 0.12 mmol). The pure product **11d** was obtained as a yellow solid (40 mg), yield: 61.5%; M.p.: 136.4–136.7 °C; ^1^H NMR (600 MHz, DMSO-*d*_6_) δ 8.61 (s, 1H, indole-2-*H*), 8.17 (s, 1H, C=C*H*), 8.00 (d, *J* = 7.8 Hz, 1H, indole-4-*H*), 7.80 (d, *J* = 7.9 Hz, 1H, coumarin-5-*H*), 7.63–7.60 (m, 2H, indole-7-*H*, coumarin-7-*H*), 7.40–7.38 (m, 3H, coumarin-8-*H*, benzene-2,4-2*H*), 7.37–7.35 (m, 2H, coumarin-6-*H*, benzene-5-*H*), 7.31 (t, *J* = 7.6 Hz, 1H, indole-6-*H*), 7.27 (t, *J* = 7.4 Hz, 1H, indole-5-*H*), 7.22 (d, *J* = 6.8 Hz, 1H, benzene-6-*H*), 5.77 (s, 1H, coumarin-3-*H*), 5.67 (s, 2H, C*H*_2_), 3.89 (t, *J* = 5.0 Hz, 4H, piperazine-3,3′-(C*H*_2_)_2_), 3.38 (t, *J* = 5.0 Hz, 4H, piperazine-2,2′-(C*H*_2_)_2_) ppm; ^13^C NMR (151 MHz, DMSO-*d*_6_) δ 164.10, 161.44, 160.65, 154.11, 143.51, 139.84, 136.41, 133.83, 132.90, 132.33, 131.15, 128.27, 128.07, 127.63, 126.38, 125.84, 124.26, 124.05, 122.41, 119.48, 118.73, 117.70, 116.16, 111.86, 110.15, 97.59, 97.46, 50.74, 49.70, 44.99 ppm; HRMS (ESI) calculated for C_32_H_25_ClN_4_O_3_ [M + H]^+^, 549.1688; found, 549.1687.

#### 3.2.23. Synthesis of (*Z*)-3-(1-(4-fluorobenzyl)-1*H*-indol-3-yl)-2-(4-(2-oxo-2*H*-chromen-4-yl)piperazine-1-carbonyl)acrylonitrile (**11e**)

Compound **11e** was prepared according to the procedure described for compound **5a**, starting from intermediate **3** (35 mg, 0.12 mmol) and **10e** (30 mg, 0.12 mmol). The pure product **11e** was obtained as a yellow solid (45 mg), yield: 71.4%; M.p.: 163.2–163.6 °C; ^1^H NMR (600 MHz, DMSO-*d*_6_) δ 8.58 (s, 1H, indole-2-*H*), 8.17 (s, 1H, C=C*H*), 7.98 (d, *J* = 7.8 Hz, 1H, indole-4-*H*), 7.79 (d, *J* = 7.9 Hz, 1H, coumarin-5-*H*), 7.65–7.60 (m, 2H, indole-7-*H*, coumarin-7-*H*), 7.40 (d, *J* = 8.3 Hz, 1H, coumarin-8-*H*), 7.37–7.34 (m, 3H, coumarin-6-*H*, benzene-2,6-2*H*), 7.30 (t, *J* = 7.5 Hz, 1H, indole-6-*H*), 7.26 (t, *J* = 7.4 Hz, 1H, indole-5-*H*), 7.19 (t, *J* = 8.7 Hz, 2H, benzene-3,5-2*H*), 5.77 (s, 1H, coumarin-3-*H*), 5.63 (s, 2H, C*H*_2_), 3.88 (t, *J* = 5.0 Hz, 4H, piperazine-3,3′-(C*H*_2_)_2_), 3.38 (t, *J* = 5.0 Hz, 4H, piperazine-2,2′-(C*H*_2_)_2_) ppm; ^13^C NMR (151 MHz, DMSO-*d*_6_) δ 164.13, 162.97, 161.44, 161.35, 160.64, 154.11, 143.55, 136.39, 133.48, 132.78, 132.33, 130.02, 129.96, 128.12, 125.84, 124.25, 123.96, 122.36, 119.41, 118.78, 117.70, 116.12, 115.98, 111.92, 110.04, 97.45, 97.32, 50.73, 49.65, 44.98 ppm; HRMS (ESI) calculated for C_32_H_25_FN_4_O_3_ [M + H]^+^, 533.1983; found, 533.1983.

#### 3.2.24. Synthesis of (*Z*)-3-(1-(4-chlorobenzyl)-1*H*-indol-3-yl)-2-(4-(2-oxo-2*H*-chromen-4-yl)piperazine-1-carbonyl)acrylonitrile (**11f**)

Compound **11f** was prepared according to the procedure described for compound **5a**, starting from intermediate **3** (35 mg, 0.12 mmol) and **10f** (32 mg, 0.12 mmol). The pure product **11f** was obtained as a yellow solid (44 mg), yield: 67.7%; M.p.: 123.1–123.8 °C; ^1^H NMR (600 MHz, DMSO-*d*_6_) δ 8.59 (s, 1H, indole-2-*H*), 8.17 (s, 1H, C=C*H*), 7.99 (d, *J* = 7.7 Hz, 1H, indole-4-*H*), 7.80 (d, *J* = 8.0 Hz, 1H, coumarin-5-*H*), 7.63–7.59 (m, 2H, indole-7-*H*, coumarin-7-*H*), 7.43–7.39 (m, 3H, coumarin-8-*H*, benzene-3,5-2*H*), 7.36 (t, *J* = 7.6 Hz, 1H, coumarin-6-*H*), 7.30 (d, *J* = 8.5 Hz, 2H, benzene-2,6-2*H*), 7.28–7.24 (m, 2H, indole-5-*H*, indole-6-*H*), 5.77 (s, 1H, coumarin-3-*H*), 5.66 (s, 2H, C*H*_2_), 3.88 (t, *J* = 5.0 Hz, 4H, piperazine-3,3′-(C*H*_2_)_2_), 3.38 (t, *J* = 5.0 Hz, 4H, piperazine-2,2′-(C*H*_2_)_2_) ppm; ^13^C NMR (151 MHz, DMSO-*d*_6_) δ 164.11, 161.43, 160.64, 154.11, 143.52, 136.40, 136.33, 132.94, 132.86, 132.33, 129.63, 129.22, 128.11, 125.84, 124.25, 124.00, 122.38, 119.44, 118.76, 117.70, 116.16, 111.89, 110.10, 97.46, 97.43, 50.73, 49.68, 45.01 ppm; HRMS (ESI) calculated for C_32_H_25_ClN_4_O_3_ [M + H]^+^, 549.1688; found, 549.1688.

#### 3.2.25. Synthesis of (Z)-3-(1-(4-bromobenzyl)-1H-indol-3-yl)-2-(4-(2-oxo-2H-chromen-4-yl)piperazine-1-carbonyl)acrylonitrile (**11g**)

Compound **11g** was prepared according to the procedure described for compound **5a**, starting from intermediate **3** (50 mg, 0.17 mmol) and **10g** (53 mg, 0.17 mmol). The pure product **11g** was obtained as a yellow solid (72 mg), yield: 72.0%; M.p.: 176.2–177.0 °C; ^1^H NMR (400 MHz, DMSO-*d*_6_) δ 8.59 (s, 1H, indole-2-*H*), 8.18 (s, 1H, C=C*H*), 7.99 (d, *J* = 7.3 Hz, 1H, indole-4-*H*), 7.80 (d, *J* = 8.0 Hz, 1H, coumarin-5-*H*), 7.62–7.58 (m, 2H, indole-7-*H*, coumarin-7-*H*), 7.55 (d, *J* = 8.2 Hz, 2H, benzene-3,5-2*H*), 7.41–7.34 (m, 2H, coumarin-6-*H*, coumarin-8-*H*), 7.30–7.26 (m, 2H, indole-5-*H*, indole-6-*H*), 7.24 (d, *J* = 8.2 Hz, 2H, benzene-2,6-2*H*), 5.77 (s, 1H, coumarin-3-*H*), 5.64 (s, 2H, C*H*_2_), 3.89 (t, *J* = 5.0 Hz, 4H, piperazine-3,3′-(C*H*_2_)_2_), 3.38 (t, *J* = 5.0 Hz, 4H, piperazine-2,2′-(C*H*_2_)_2_) ppm; ^13^C NMR (101 MHz, DMSO-*d*_6_) δ 164.10, 161.45, 160.64, 154.09, 143.54, 136.77, 136.35, 132.86, 132.33, 132.14, 129.92, 128.11, 125.84, 124.26, 124.00, 122.39, 121.42, 119.43, 118.78, 117.70, 116.13, 111.90, 110.08, 97.46, 97.36, 50.73, 49.71, 44.95 ppm; HRMS (ESI) calculated for C_32_H_25_BrN_4_O_3_ [M + H]^+^, 593.1183; found, 593.1180.

#### 3.2.26. Synthesis of (Z)-3-(1-(4-iodobenzyl)-1H-indol-3-yl)-2-(4-(2-oxo-2H-chromen-4-yl)piperazine-1-carbonyl)acrylonitrile (**11h**)

Compound **11h** was prepared according to the procedure described for compound **5a**, starting from intermediate **3** (50 mg, 0.17 mmol) and **10h** (61 mg, 0.17 mmol). The pure product **11h** was obtained as a yellow solid (73 mg), yield: 68.1%; M.p.: 203.5–204.4 °C; ^1^H NMR (400 MHz, DMSO-*d*_6_) δ 8.58 (s, 1H, indole-2-*H*), 8.17 (s, 1H, C=C*H*), 7.99 (d, *J* = 7.0 Hz, 1H, indole-4-*H*), 7.80 (d, *J* = 7.1 Hz, 1H, coumarin-5-*H*), 7.71 (d, *J* = 8.2 Hz, 2H, benzene-3,5-2*H*), 7.63–7.57 (m, 2H, indole-7-*H*, coumarin-7-*H*), 7.41–7.36 (m, 2H, coumarin-6-*H*, coumarin-8-*H*), 7.31–7.26 (m, 2H, indole-5-*H*, indole-6-*H*), 7.08 (d, *J* = 8.2 Hz, 2H, benzene-2,6-2*H*), 5.77 (s, 1H, coumarin-3-*H*), 5.62 (s, 2H, C*H*_2_), 3.88 (t, *J* = 5.0 Hz, 4H, piperazine-3,3′-(C*H*_2_)_2_), 3.38 (t, *J* = 5.0 Hz, 4H, piperazine-2,2′-(C*H*_2_)_2_) ppm; ^13^C NMR (101 MHz, DMSO-*d*_6_) δ 164.10, 161.45, 160.64, 154.09, 143.54, 137.99, 137.16, 136.36, 132.88, 132.34, 129.99, 128.10, 125.85, 124.27, 123.99, 122.38, 119.43, 118.78, 117.70, 116.13, 111.91, 110.06, 97.45, 97.34, 94.32, 50.73, 49.85, 44.95 ppm; HRMS (ESI) calculated for C_32_H_25_IN_4_O_3_ [M + H]^+^, 641.1044; found, 641.1042.

#### 3.2.27. Synthesis of (*Z*)-3-(1-(2,4-dichlorobenzyl)-1*H*-indol-3-yl)-2-(4-(2-oxo-2*H*-chromen-4-yl)piperazine-1-carbonyl)acrylonitrile (**11i**)

Compound **11i** was prepared according to the procedure described for compound **5a**, starting from intermediate **3** (50 mg, 0.17 mmol) and **10i** (51 mg, 0.17 mmol). The pure product **11i** was obtained as a yellow solid (84 mg), yield: 85.7%; M.p.: 176.1–176.8 °C; ^1^H NMR (600 MHz, DMSO-*d*_6_) δ 8.53 (s, 1H, indole-2-*H*), 8.18 (s, 1H, C=C*H*), 8.01 (d, *J* = 7.6 Hz, 1H, indole-4-*H*), 7.79 (d, *J* = 7.9 Hz, 1H, coumarin-5-*H*), 7.72 (s, 1H, benzene-3-*H*), 7.62 (t, *J* = 7.7 Hz, 1H, coumarin-7-*H*), 7.58 (d, *J* = 7.8 Hz, 1H, indole-7-*H*), 7.41–7.39 (m, 2H, benzene-5-*H*, coumarin-8-*H*), 7.36 (t, *J* = 7.5 Hz, 1H, coumarin-6-*H*), 7.33–7.26 (m, 2H, indole-5,6-2*H*), 7.01 (d, *J* = 8.3 Hz, 1H, benzene-6-*H*), 5.76 (s, 1H, coumarin-3-*H*), 5.71 (s, 2H, C*H*_2_), 3.88 (t, *J* = 5.0 Hz, 4H, piperazine-3,3′-(C*H*_2_)_2_), 3.38 (t, *J* = 5.0 Hz, 4H, piperazine-2,2′-(C*H*_2_)_2_) ppm; ^13^C NMR (151 MHz, DMSO-*d*_6_) δ 164.03, 161.47, 160.65, 154.09, 143.41, 136.49, 134.00, 133.87, 133.57, 132.94, 132.34, 131.09, 129.73, 128.34, 127.94, 125.84, 124.27, 124.17, 122.51, 119.49, 118.68, 117.69, 116.14, 111.69, 110.21, 97.70, 97.42, 50.72, 47.89, 44.97 ppm; HRMS (ESI) calculated for C_32_H_24_Cl_2_N_4_O_3_ [M + H]^+^, 583.1298; found, 583.1297.

#### 3.2.28. Synthesis of (*Z*)-3-(1-(cyanomethyl)-1*H*-indol-3-yl)-2-(4-(2-oxo-2*H*-chromen-4-yl)piperazine-1-carbonyl)acrylonitrile (**13a**)

Compound **13a** was prepared according to the procedure described for compound **5a**, starting from intermediate **3** (50 mg, 0.17 mmol) and **12a** (31 mg, 0.17 mmol). The pure product **13a** was obtained as a yellow solid (52 mg), yield: 66.7%; M.p.: 205.2–205.7 °C; ^1^H NMR (600 MHz, DMSO-*d*_6_) δ 8.57 (s, 1H, indole-2-*H*), 8.17 (s, 1H, C=C*H*), 8.03 (d, *J* = 7.9 Hz, 1H, indole-4-*H*), 7.80 (d, *J* = 7.8 Hz, 1H, coumarin-5-*H*), 7.74 (d, *J* = 8.2 Hz, 1H, indole-7-*H*), 7.62 (t, *J* = 7.6 Hz, 1H, coumarin-7-*H*), 7.44 (t, *J* = 7.6 Hz, 1H, indole-6-*H*), 7.40 (d, *J* = 8.2 Hz, 1H, coumarin-8-*H*), 7.38–7.34 (m, 2H, coumarin-6-*H*, indole-5-*H*), 5.81 (s, 2H, C*H*_2_C≡N), 5.77 (s, 1H, coumarin-3-*H*), 3.89 (t, *J* = 5.0 Hz, 4H, piperazine-3,3′-(C*H*_2_)_2_), 3.39 (t, *J* = 5.0 Hz, 4H, piperazine-2,2′-(C*H*_2_)_2_) ppm; ^13^C NMR (151 MHz, DMSO-*d*_6_) δ 163.71, 161.43, 160.64, 154.11, 142.82, 135.99, 132.32, 132.05, 127.91, 125.83, 124.57, 124.26, 122.91, 119.70, 118.28, 117.70, 116.31, 116.15, 111.29, 111.04, 99.25, 97.49, 50.71, 44.98, 35.13 ppm; HRMS (ESI) calculated for C_27_H_21_N_5_O_3_ [M + H]^+^, 464.1717; found, 464.1718.

#### 3.2.29. Synthesis of (*Z*)-3-(1-(2-hydroxyethyl)-1*H*-indol-3-yl)-2-(4-(2-oxo-2*H*-chromen-4-yl)piperazine-1-carbonyl)acrylonitrile (**13b**)

Compound **13b** was prepared according to the procedure described for compound **5a**, starting from intermediate **3** (60 mg, 0.20 mmol) and **12b** (38 mg, 0.20 mmol). The pure product **13b** was obtained as a yellow solid (77 mg), yield: 81.4%; M.p.: 215.7–216.9 °C; ^1^H NMR (600 MHz, DMSO-*d*_6_) δ 8.49 (s, 1H, indole-2-*H*), 8.16 (s, 1H, C=C*H*), 7.96 (d, *J* = 7.7 Hz, 1H, indole-4-*H*), 7.79 (d, *J* = 7.5 Hz, 1H, coumarin-5-*H*), 7.66 (d, *J* = 8.0 Hz, 1H, indole-7-*H*), 7.61 (t, *J* = 7.3 Hz, 1H, coumarin-7-*H*), 7.39 (d, *J* = 8.1 Hz, 1H, coumarin-8-*H*), 7.36 (t, *J* = 7.5 Hz, 1H, coumarin-6-*H*), 7.32 (t, *J* = 7.4 Hz, 1H, indole-6-*H*), 7.26 (t, *J* = 7.2 Hz, 1H, indole-5-*H*), 5.76 (s, 1H, coumarin-3-*H*), 4.99 (s, 1H, O*H*), 4.40 (t, *J* = 4.9 Hz, 2H, C*H*_2_CH_2_OH), 3.88 (t, *J* = 5.0 Hz, 4H, piperazine-3,3′-(C*H*_2_)_2_), 3.78 (t, *J* = 4.9 Hz, 2H, CH_2_C*H*_2_OH), 3.38 (t, *J* = 5.0 Hz, 4H, piperazine-2,2′-(C*H*_2_)_2_) ppm; ^13^C NMR (151 MHz, DMSO-*d*_6_) δ 164.35, 161.43, 160.64, 154.11, 143.83, 136.66, 133.75, 132.31, 128.03, 125.84, 124.25, 123.63, 122.18, 119.14, 119.02, 117.69, 116.17, 111.78, 109.42, 97.45, 96.23, 60.27, 50.74, 49.84, 44.99 ppm; HRMS (ESI) calculated for C_27_H_24_N_4_O_4_ [M + H]^+^, 469.1870; found, 469.1872.

#### 3.2.30. Synthesis of ethyl (*Z*)-2-(3-(2-cyano-3-oxo-3-(4-(2-oxo-2*H*-chromen-4-yl)piperazin-1-yl) prop-1-en-1-yl)-1*H*-indol-1-yl)acetatee (**13c**)

Compound **13c** was prepared according to the procedure described for compound **5a**, starting from intermediate **3** (65 mg, 0.22 mmol) and **12c** (51 mg, 0.22 mmol). The pure product **13c** was obtained as a yellow solid (75 mg), yield: 67.0%; M.p.: 183.0–183.6 °C; ^1^H NMR (600 MHz, DMSO-*d*_6_) δ 8.49 (s, 1H, indole-2-*H*), 8.16 (s, 1H, C=C*H*), 7.98 (d, *J* = 7.8 Hz, 1H, indole-4-*H*), 7.80 (d, *J* = 8.0 Hz, 1H, coumarin-5-*H*), 7.61 (t, *J* = 7.7 Hz, 1H, coumarin-7-*H*), 7.56 (d, *J* = 8.1 Hz, 1H, indole-7-*H*), 7.40 (d, *J* = 8.3 Hz, 1H, coumarin-8-*H*), 7.36 (t, *J* = 7.5 Hz, 1H, coumarin-6-*H*), 7.32 (t, *J* = 7.5 Hz, 1H, indole-6-*H*), 7.28 (t, *J* = 7.4 Hz, 1H, indole-5-*H*), 5.76 (s, 1H, coumarin-3-*H*), 5.40 (s, 2H, C*H*_2_COO), 4.18 (q, *J* = 7.0 Hz, 2H, C*H*_2_CH_3_), 3.88 (t, *J* = 5.0 Hz, 4H, piperazine-3,3′-(C*H*_2_)_2_), 3.38 (t, *J* = 5.0 Hz, 4H, piperazine-2,2′-(C*H*_2_)_2_), 1.23 (t, *J* = 7.0 Hz, 3H, CH_2_C*H*_3_) ppm; ^13^C NMR (151 MHz, DMSO-*d*_6_) δ 168.68, 164.08, 161.43, 160.65, 154.11, 143.44, 137.02, 133.69, 132.33, 127.74, 125.85, 124.26, 123.96, 122.33, 119.19, 118.61, 117.70, 116.16, 111.51, 110.12, 97.57, 97.45, 61.73, 50.73, 48.22, 44.96, 14.51 ppm; HRMS (ESI) calculated for C_29_H_26_N_4_O_5_ [M + H]^+^, 511.1976; found, 511.1975.

### 3.3. Biological Assay

#### 3.3.1. Antibacterial Activity

All the target PHCIs were evaluated for their antibacterial activities against five Gram-positive bacteria and five Gram-negative bacteria. The bacterial suspension was adjusted with sterile saline to a concentration of 1 × 10^5^ CFU/mL. These compounds and norfloxacin were separately dissolved in dimethyl sulfoxide to prepare the stock solutions. The stock solutions were serially diluted in Mueller–Hinton broth (Guangdong huaikai microbial sci.& tech co., Ltd., Guangzhou, Guangdong, China) to the desired concentrations in 96-well plate. The plates were followed by the addition of ~10^5^ CFU/mL bacterial suspension (100 μL) in each well and incubated at 37 °C for 24 h to determine minimum inhibitory concentrations (MICs).

#### 3.3.2. Resistance Study

The strain of *P. aeruginosa* 27,853 was exposed to sub-MICs of derivative **11f** for sustained passages, which were determined every 24 h after propagation of *P. aeruginosa* 27,853 cultures and then the MICs of compound **11f** were obtained against each passage of *P. aeruginosa* 27853. The process was repeated for 20 passages, using norfloxacin as control group.

#### 3.3.3. Hemolytic Assay

The fresh human blood was centrifuged at 5000 r/min for 5 min and laved three times with normal saline to collect erythrocytes, which was subsequently resuspended to prepare 5% RBC suspension. The each well of 96-well plates was injected with 100 μL red cell suspension. PHCI **11f** (512 μg/mL, 100 μL) was added to the first column of plates and was double-serially diluted. The well without compound and the well added with 100 μL 0.1% Triton X-100 were used as negative and positive control, respectively. The plates were incubated for different times at 37 °C and then were centrifuged to extract the liquid supernatant of each hole. The absorbance of liquid supernatants was measured at 540 nm by microplate reader. The hemolysis ratio could be calculated as (A − A_0_)/(A_total_ − A_0_) × 100 (A: the absorbance of tested samples, A_0_: the absorbance of negative control, A_total_: the absorbance of positive control).

#### 3.3.4. Outer Membrane Permeability

The *N*-phenylnaphthylamine (NPN) dye (10 mM, 50 μL) was added to *P. aeruginosa* 27,853 cell suspension (~10^6^ CFU/mL, 1 mL) for the incubation of 30 min. After the cultivation, the bacterial suspension was centrifuged to isolate cell pellets, and the obtained cell pellets were washed and resuspended in phosphate buffer saline. Then PHCI **11f** at different concentrations were added and the fluorescence intensity at different time intervals were measured at an excitation wavelength of 350 nm and an emission wavelength of 420 nm. Dimethyl sulfoxide was used as a negative control.

#### 3.3.5. Inner Membrane Permeability

The *P. aeruginosa* 27,853 cells were cultured, washed, and resuspended in 5 mM glucose and 5 mM HEPES buffer (pH = 7.2) at 1:1 ratio. The bacterial suspension (1 mL) was added with propidium iodide (PI) dye (10 μM, 50 μL) and various concentrations of **11f** rendering that the final concentrations of **11f** in suspension should be 0, 0.5, 1, 2, 4, and 8 μg/mL. After coincubation for about 30 min, the fluorescence intensities were measured at excitation wavelength of 535 nm and emission wavelength of 617 nm. Dimethyl sulfoxide without **11f** was used as a negative control. All samples were run in triplicates.

#### 3.3.6. Depolarization of Membrane

The *P. aeruginosa* 27,853 cells were cultured to the mid log phase, and then were washed and resuspended in a buffer solution (5 mM HEPES buffer, 5 mM glucose, *v*/*v*, 1:1). The depolarization dye DiSC3(5) (10 μM, 50 μL) and bacterial suspension (1 mL) were coincubated at 37 °C for 1 h, following which 100 mM KCl was added to the cell suspensions. After preincubation with dye, the suspensions were treated with various concentrations of **11f** and the fluorescence of suspensions at different concentrations were monitored with an excitation wavelength of 622 nm and an emission wavelength of 670 nm.

#### 3.3.7. Cell Morphology Observation

The samples for SEM observation were prepared according to previously published method with slight modifications [58]. The *P. aeruginosa* 27,853 cells were treated with PHCI **11f** at 2 μg/mL for different times (3, 6 and 12 h), and then were washed three times with phosphate buffer saline to get cell pellets. The cell pellets were fixed with 2.5% glutaraldehyde at 4 °C for 12 h, then dehydrated with different gradients of ethanol (30, 50, 70, 90, and 100%). After that, dehydrated cells were transferred to the silicon chip and dried. No compound treatment was used as negative control. The samples were coated with gold and the cell morphologies were observed through a scanning electron microscope (Phenom Pro10102, Phenom-World, Eindhoven, The Netherlands).

#### 3.3.8. The Leakage of Intracellular Components

The *P. aeruginosa* 27,853 suspensions were incubated with increased concentrations of PHCI **11f** (0, 1, 2, 4, 6, and 8 μg/mL) at 37 °C for 8 h. The bacterial suspension added with phosphate buffer saline was used as a control. After cultivation, the suspensions were centrifuged, then the supernatant was collected. The content of leaked proteins in supernatant was determined according to standard Bradford assay. A volume of 100 μL supernatant was added into 96-well plate and the OD_260_ values were measured under a microplate reader to evaluate the leaked nucleic acids. All samples were conducted in triplicates.

#### 3.3.9. The Oxidative Stress Assay

Intracellular ROS accumulation was measured using DCFH-DA as fluorescent probe. The 10^6^ CFU/mL of bacterial cells were treated with increased concentrations of PHCI **11f** for 6 h at 37 °C. Following treatment, the cells were washed with phosphate buffer saline and incubated with DCFH-DA dye (100 μM, 10 μL) for 30 min in dark. The fluorescence intensity which was positively related to the ROS levels was measured at an excitation wavelength of 488 nm and an emission wavelength of 522 nm.

#### 3.3.10. Metabolic Activity

The *P. aeruginosa* 27,853 cells (~10^5^ CFU/mL) were treated with increasing concentrations of PHCI **11f** (0, 1, 2, 4, and 8 μg/mL) for 6 h at 37 °C. The cell suspensions (1 mL) were incubated with alamar blue (250 μg/mL, 100 μL) for an additional 20 min. The fluorescence intensity of each group was measured at excitation wavelength of 550 nm and emission wavelength of 590 nm.

#### 3.3.11. Supramolecular Interaction of Indolylcyanoenone **11f** with DNA

The supramolecular interaction of DNA and **11f** was explored through UV–vis spectra using calf thymus DNA as a substrate. The compound **11f** was dissolved in DMF to prepare a stock solution (5 × 10^−3^ mol/L). The DNA was dissolved in Tris-HCl buffer solution at a concentration of 7.1 × 10^−5^ mol/L. Compound **11f** was added to the DNA solution to make the final concentrations of **11f** ranging from 0 to 1.333 × 10^−5^ mol/L.

Acridine orange (AO) was added to calf thymus DNA solution (*c*(DNA) = 7.1 × 10^−5^ mol/L). Compound **11f** was added to the above AO-DNA system to make the final concentrations of **11f** ranging from 0 to 1.333 × 10^−5^ mol/L. The fluorescence spectra were recorded under each gradient at an excitation wavelength of 488 nm.

#### 3.3.12. Molecular Docking

DNA gyrase (Code: 2XCS) in pdb format was downloaded from Protein Data Bank (https://www.rcsb.org/structure/2XCS, accessed on 4 August 2022). AutoDockTools 1.5.6 was used to perform docking study. The 3D and 2D diagrams of the docking result was exported by PyMOL 2.3.4 and Discovery Studio 4.5, respectively.

## 4. Conclusions

In conclusion, a series of piperazine hybridized coumarin indolylcyanoenones as new antibacterial scaffolds were developed. Antibacterial assessment manifested that most of PHCIs exhibited significant inhibitory efficiencies on the growth of *P. aeruginosa* and *P. aeruginosa* 27853, especially 4-chlorobenzyl derivative **11f**, which was four times more effective than norfloxacin in inhibiting *P. aeruginosa* 27,853 with an MIC as low as 1 μg/mL. Noticeably, the active molecule **11f** showed a safe hemolysis level and hardly induced bacterial resistance, displaying great potential as a drug candidate. Preliminary mechanistic exploration indicated that PHCI **11f** could destroy bacterial membrane integrity, resulting in changes of membrane potential and permeability, deformation of cell membrane, and leakage of intracellular contents. Destruction of membrane interfered the homeostasis of intracellular environment, further causing excessive ROS production and metabolic vitality decline. Moreover, subsequent studies found that PHCI **11f** could also insert into DNA by forming a stable DNA–**11f** supramolecular complex and target DNA gyrase through multiple noncovalent interactions to hinder bacterial replication. These synergistic antibacterial advantages provided a new inspiration for the further exploitation of piperazine hybridized coumarin indolylcyanoenones as effective antibacterial agents.

## Figures and Tables

**Table 1:** Antibacterial data (MIC, μg/mL) for the target piperazine hybridized coumarin indolylcyanoenones **5a–g**, **7a–f**, **9a–c**, **11a–i,** and **13a–c**.

Compounds	Gram-Positive Bacteria ^a^					Gram-Negative Bacteria ^b^				
	*S. a.*	*S. a.* 26003	*S. a.* 25923	*S. a.* 6538	*E. f.*	*E. c.* 9027	*E. c.* 25922	*P. a.*	*P. a.* 27853	*A. b.*
**5a**	128	16	16	16	64	64	32	4	8	16
**5b**	64	128	64	4	32	32	128	16	16	64
**5c**	64	128	128	128	64	64	16	4	4	64
**5d**	32	32	64	64	16	64	32	16	4	64
**5e**	16	4	2	32	32	64	64	4	4	16
**5f**	64	4	16	64	64	64	64	2	8	32
**5g**	64	16	128	16	64	32	128	16	4	64
**7a**	64	32	256	128	32	64	64	8	8	8
**7b**	64	32	16	64	64	128	8	8	4	64
**7c**	64	32	128	32	32	64	256	16	8	64
**7d**	32	16	32	64	64	64	32	4	4	64
**7e**	128	32	128	64	64	32	16	8	4	64
**7f**	128	8	4	32	32	128	128	4	4	32
**9a**	64	16	128	8	32	64	32	16	4	64
**9b**	64	64	32	16	64	64	16	8	4	64
**9c**	64	16	64	4	32	64	64	8	8	32
**11a**	8	32	128	32	64	32	64	4	8	64
**11b**	16	32	128	64	64	32	128	8	8	64
**11c**	4	32	128	4	64	32	128	4	8	64
**11d**	4	64	128	16	32	32	64	4	8	64
**11e**	128	32	16	64	8	32	64	8	2	32
**11f**	64	32	128	32	32	64	128	4	1	64
**11g**	8	32	32	64	16	32	64	4	2	64
**11h**	32	16	16	64	16	32	64	8	8	32
**11i**	16	16	16	64	64	32	16	2	2	64
**13a**	4	16	32	8	64	32	64	4	8	16
**13b**	128	16	8	32	128	64	64	4	8	64
**13c**	64	32	8	32	64	32	128	4	16	64
Norfloxacin	2	2	1	2	2	8	2	4	4	8

^a^*S. a.*, *Staphylococcus aureus*; *S. a.* 26003, *Staphylococcus aureus* CMCC(B) 26003; *S. a.* 25923, *Staphylococcus aureus* ATCC 25923; S. a. 6538, *Staphylococcus aureus* ATCC 6538; *E. f*., *Enterococcus faecalis*; ^b^ *E. c.* 9027, *Escherichia coli* ATCC 9027; *E. c.* 25922, *Escherichia coli* ATCC 25922; *P. a*., *Pseudomonas aeruginosa*; *P. a.* 27853, *Pseudomonas aeruginosa* ATCC 27853; *A. b*., *Acinetobacter baumannii*.

## Data Availability

Not applicable.

## Supplementary Materials

The following supporting information can be downloaded at: https://www.mdpi.com/article/10.3390/molecules28062511/s1, Figures S1−S5: The molecular docking results of DNA gyrase and PHCIs **5a**, **5b**, **7a**, **9a**, and **13a**.

## Sample Availability

Samples of the compounds are available from the authors.

## References

[1] Han D.L., Liu X.M., Wu S.L. (2022). Metal organic framework-based antibacterial agents and their underlying mechanisms. Chem. Soc. Rev..

[2] Lázár V., Snitser O., Barkan D., Kishony R. (2022). Antibiotic combinations reduce *Staphylococcus aureus* clearance. Nature.

[3] Brown E.D., Wright G.D. (2016). Antibacterial drug discovery in the resistance era. Nature.

[4] Sun H., Huang S.Y., Jeyakkumar P., Cai G.X., Fang B., Zhou C.H. (2022). Natural berberine-derived azolyl ethanols as new structural antibacterial agents against drug-resistant *Escherichia coli*. J. Med. Chem..

[5] Levin-Reisman I., Ronin I., Gefen O., Braniss I., Shoresh N., Balaban N.Q. (2017). Antibiotic tolerance facilitates the evolution of resistance. Science.

[6] Shukla R., Lavore F., Maity S., Derks M.G.N., Jones C.R., Vermeulen B.J.A., Melcrová A., Morris M.A., Becker L.M., Wang X. (2022). Teixobactin kills bacteria by a two-pronged attack on the cell envelope. Nature.

[7] Li F.F., Zhao W.H., Tangadanchu V.K.R., Meng J.P., Zhou C.H. (2022). Discovery of novel phenylhydrazone-based oxindole-thiolazoles as potent antibacterial agents toward *Pseudomonas aeruginosa*. Eur. J. Med. Chem..

[8] Lin S.M., Wade J.D., Liu S.P. (2021). De novo design of flavonoid-based mimetics of cationic antimicrobial peptides: Discovery, development, and applications. Acc. Chem. Res..

[9] Sun H., Ansari M.F., Fang B., Zhou C.H. (2021). Natural berberine-hybridized benzimidazoles as novel unique bactericides against *Staphylococcus aureus*. J. Agric. Food Chem..

[10] Zhang L., Peng X.M., Damu G.L.V., Geng R.X., Zhou C.H. (2014). Comprehensive review in current developments of imidazole-based medicinal chemistry. Med. Res. Rev..

[11] Sahoo C.R., Sahoo J., Mahapatra M., Lenka D., Sahu P.K., Dehury B., Padhy R.N., Paidesetty S.K. (2021). Coumarin derivatives as promising antibacterial agent(s). Arab. J. Chem..

[12] Yang X.C., Hu C.F., Zhang P.L., Li S., Hu C.S., Geng R.X., Zhou C.H. (2022). Coumarin thiazoles as unique structural skeleton of potential antimicrobial agents. Bioorg. Chem..

[13] Peng X.M., Damu G.L.V., Zhou C.H. (2013). Current developments of coumarin compounds in medicinal chemistry. Curr. Pharm. Des..

[14] Broeck A.V., McEwen A.G., Chebaro Y., Potier N., Lamour V. (2019). Structural basis for DNA gyrase interaction with coumermycin A1. J. Med. Chem..

[15] Peng X.M., Kumar K.V., Damu G.L.V., Zhou C.H. (2016). Coumarin-derived azolyl ethanols: Synthesis, antimicrobial evaluation and preliminary action mechanism study. Sci. China Chem..

[16] Yang X.C., Zeng C.M., Avula S.R., Peng X.M., Geng R.X., Zhou C.H. (2023). Novel coumarin aminophosphonates as potential multitargeting antibacterial agents against *Staphylococcus aureus*. Eur. J. Med. Chem..

[17] Hu Y., Shen Y.F., Wu X.H., Tu X., Wang G.X. (2018). Synthesis and biological evaluation of coumarin derivatives containing imidazole skeleton as potential antibacterial agents. Eur. J. Med. Chem..

[18] Yang X.C., Zhang P.L., Kumar K.V., Li S., Geng R.X., Zhou C.H. (2022). Discovery of unique thiazolidinone-conjugated coumarins as novel broad spectrum antibacterial agents. Eur. J. Med. Chem..

[19] Hu C.F., Zhang P.L., Sui Y.F., Lv J.S., Ansari M.F., Battini N., Li S., Zhou C.H., Geng R.X. (2020). Ethylenic conjugated coumarin thiazolidinediones as new efficient antimicrobial modulators against clinical methicillin-resistant *Staphylococcus aureus*. Bioorg. Chem..

[20] Wang J., Ansari M.F., Zhou C.H. (2021). Identification of unique quinazolone thiazoles as novel structural scaffolds for potential gram-negative bacterial conquerors. J. Med. Chem..

[21] Tangadanchu V.K.R., Sui Y.F., Zhou C.H. (2021). Isatin-derived azoles as new potential antimicrobial agents: Design, synthesis and biological evaluation. Bioorg. Med. Chem. Lett..

[22] Yoshizawa M., Itoh T., Hori T., Kato A., Anami Y., Yoshimoto N., Yamamoto K. (2018). Identification of the Histidine residue in vitamin D receptor that covalently binds to electrophilic ligands. J. Med. Chem..

[23] Lee J.H., Wood T.K., Lee J. (2015). Roles of indole as an interspecies and interkingdom signaling molecule. Trends Microbiol..

[24] Boon N., Kaur M., Aziz A., Bradnick M., Shibayama K., Eguchi Y., Lund P.A. (2020). The signaling molecule indole inhibits induction of the AR2 acid resistance system in *Escherichia coli*. Front. Microbiol..

[25] Li Z.Z., Tangadanchu V.K.R., Battini N., Bheemanaboina R.R.Y., Zang Z.L., Zhang S.L., Zhou C.H. (2019). Indole-nitroimidazole conjugates as efficient manipulators to decrease the genes expression of methicillin-resistant *Staphylococcus aureus*. Eur. J. Med. Chem..

[26] Li H.D., Wu S., Yang X., He H.F., Wu Z.X., Song B.A., Song R.J. (2022). Synthesis, antibacterial activity, and mechanisms of novel indole derivatives containing pyridinium moieties. J. Agric. Food Chem..

[27] Chen Y.Z., Li H.X., Liu J.Y., Zhong R.C., Li H.Z., Fang S.F., Liu S.P., Lin S.M. (2021). Synthesis and biological evaluation of indole-based peptidomimetics as antibacterial agents against Gram-positive bacteria. Eur. J. Med. Chem..

[28] Qin R., Zhao Q., Han B., Zhu H.P., Peng C., Zhan G., Huang W. (2022). Indole-based small molecules as potential therapeutic agents for the treatment of fibrosis. Front. Pharmacol..

[29] Zhang P.L., Gopala L., Zhang S.L., Cai G.X., Zhou C.H. (2022). An unanticipated discovery towards novel naphthalimide corbelled aminothiazoximes as potential anti-MRSA agents and allosteric modulators for PBP2a. Eur. J. Med. Chem..

[30] Hu Y.Y., Bheemanaboina R.R.Y., Battini N., Zhou C.H. (2019). Sulfonamide-derived four-component molecular hybrids as novel DNA-targeting membrane active potentiators against clinical *Escherichia coli*. Mol. Pharmaceutics.

[31] Chen J.P., Battini N., Ansari M.F., Zhou C.H. (2021). Membrane active 7-thiazoxime quinolones as novel DNA binding agents to decrease the genes expression and exert potent antimethicillin-resistant *Staphylococcus aureus* activity. Eur. J. Med. Chem..

[32] Liang W.X., Yu Q., Zheng Z.X., Liu J.Y., Cai Q.N., Liu S.P., Lin S.M. (2022). Design and synthesis of phenyl sulfide-based cationic amphiphiles as membrane-targeting antimicrobial agents against Gram-Positive pathogens. J. Med. Chem..

[33] Yang X., Sun H., Maddili S.K., Li S., Yang R.G., Zhou C.H. (2022). Dihydropyrimidinone imidazoles as unique structural antibacterial agents for drug-resistant gram-negative pathogens. Eur. J. Med. Chem..

[34] Sui Y.F., Ansari M.F., Zhou C.H. (2021). Pyrimidinetrione-imidazoles as a unique structural type of potential agents towards *Candida albicans*: Design, synthesis and biological evaluation. Chem. Asian J..

[35] Mitcheltree M.J., Pisipati A., Syroegin E.A., Silvestre K.J., Klepacki D., Mason J.D., Terwilliger D.W., Testolin G., Pote A.R., Wu K.J.Y. (2021). A synthetic antibiotic class overcoming bacterial multidrug resistance. Nature.

[36] Wang J., Battini N., Ansari M.F., Zhou C.H. (2021). Synthesis and biological evaluation of quinazolonethiazoles as new potential conquerors towards *Pseudomonas aeruginosa*. Chin. J. Chem..

[37] Zhou X.M., Hu Y.Y., Fang B., Zhou C.H. (2023). Benzenesulfonyl thiazoloimines as unique multitargeting antibacterial agents towards *Enterococcus faecalis*. Eur. J. Med. Chem..

[38] Zhou C.H., Wang Y. (2012). Recent researches in triazole compounds as medicinal drugs. Curr. Med. Chem..

[39] Zhang C.Y., Yu R.J., Wang L.Q., Huang H.Y., Wang J.T., Liao X.W., Duan X.M., Xiong Y.S. (2022). Design, synthesis, and evaluation of aryl-thioether ruthenium polypyridine complexes: A multi-target antimicrobial agents against Gram-positive bacteria. Eur. J. Med. Chem..

[40] Sui Y.F., Ansari M.F., Fang B., Zhang S.L., Zhou C.H. (2021). Discovery of novel purinylthiazolylethanone derivatives as anti-*Candida albicans* agents through possible multifaceted mechanisms. Eur. J. Med. Chem..

[41] Cui S.F., Addla D., Zhou C.H. (2016). Novel 3-aminothiazolquinolones: Design, synthesis, bioactive evaluation, SARs, and preliminary antibacterial mechanism. J. Med. Chem..

[42] Pham D.D.D., Mojr V., Helusová M., Mikušová G., Pohl R., Dávidová E., Šanderová H., Vítovská D., Bogdanová K., Večeřová R. (2022). LEGO-Lipophosphonoxins: A novel approach in designing membrane targeting antimicrobials. J. Med. Chem..

[43] Li F.F., Zhang P.L., Tangadanchu V.K.R., Li S., Zhou C.H. (2022). Novel metronidazole-derived three-component hybrids as promising broad-spectrum agents to combat oppressive bacterial resistance. Bioorg. Chem..

[44] Nourbakhsh F., Lotfalizadeh M., Badpeyma M., Shakeri A., Soheili V. (2022). From plants to antimicrobials: Natural products against bacterial membranes. Phytother. Res..

[45] Wang J., Ansari M.F., Lin J.M., Zhou C.H. (2021). Design and synthesis of sulfanilamide aminophosphonates as novel antibacterial agents towards *Escherichia coli*. Chin. J. Chem..

[46] Liang X.Y., Battini N., Sui Y.F., Ansari M.F., Gan L.L., Zhou C.H. (2021). Aloe-emodin derived azoles as a new structural type of potential antibacterial agents: Design, synthesis, and evaluation of the action on membrane, DNA, and MRSA DNA isomerase. RSC Med. Chem..

[47] Zhong C., Zhang F.Y., Yao J., Zhu Y.W., Zhu N.Y., Zhang J.Y., Ouyang X., Zhang T.Y., Li B.B., Xie J.Q. (2021). New antimicrobial peptides with repeating unit against multidrug resistant bacteria. ACS Infect. Dis..

[48] Wang J., Zhang P.L., Ansari M.F., Li S., Zhou C.H. (2021). Molecular design and preparation of 2-aminothiazole sulfanilamide oximes as membrane active antibacterial agents for drug resistant *Acinetobacter baumannii*. Bioorg. Chem..

[49] Li H.X., Liu J.Y., Liu C.F., Li H.Z., Luo J.C., Fang S.F., Chen Y.Z., Zhong R.C., Liu S.P., Lin S.M. (2021). Design, synthesis, and biological evaluation of membrane-active bakuchiol derivatives as effective broad-spectrum antibacterial agents. J. Med. Chem..

[50] Xie Y.P., Sangaraiah N., Meng J.P., Zhou C.H. (2022). Unique Carbazole-oxadiazole derivatives as new potential antibiotics for combating gram-positive and -negative bacteria. J. Med. Chem..

[51] Xing M., Liu S., Yu Y.P., Guo L., Wang Y., Feng Y.G., Fei P., Kang H.B., Ali M.A. (2022). Antibacterial mode of *Eucommia ulmoides* male flower extract against *Staphylococcus aureus* and its application as a natural preservative in cooked beef. Front. Microbiol..

[52] Zhang P.L., Lv J.S., Ansari M.F., Battini N., Cai G.X., Zhou C.H. (2021). Synthesis of naphthalimide triazoles with a novel structural framework and their anti-*Aspergillus fumigatus* effects. Sci. Sin. Chim..

[53] Zhang E., Bai P.Y., Cui D.Y., Chu W.C., Hua Y.G., Liu Q., Yin H.Y., Zhang Y.J., Qin S.S., Liu H.M. (2018). Synthesis and bioactivities study of new antibacterial peptide mimics: The dialkyl cationic amphiphiles. Eur. J. Med. Chem..

[54] Pasero C., D’Agostino I., Luca F.D., Zamperini C., Deodato D., Truglio G.I., Sannio F., Prete R.D., Ferraro T., Visaggio D. (2018). Alkyl-guanidine compounds as potent broad-spectrum antibacterial agents: Chemical library extension and biological characterization. J. Med. Chem..

[55] Kumar P., Shaikh A.A., Kumar P., Gupta V.K., Dhyani R., Sharma T.K., Hussain A., Gangele K., Poluri K.M., Rao K.N. (2022). Double-edged nanobiotic platform with protean functionality: Leveraging the synergistic antibacterial activity of a food-grade peptide to mitigate multidrug-resistant bacterial pathogens. ACS Appl. Mater. Interfaces.

[56] Deng Z., Bheemanaboina R.R.Y., Luo Y., Zhou C.H. (2022). Aloe emodin-conjugated sulfonyl hydrazones as novel type of antibacterial modulators against *S. aureus* 25923 through multifaceted synergistic effects. Bioorg. Chem..

[57] Zhang X.C., Zhang Z.C., Shu Q.M., Xu C., Zheng Q.Q., Guo Z., Wang C., Hao Z.X., Liu X., Wang G.Q. (2021). Copper clusters: An effective antibacterial for eradicating multidrug-resistant bacterial infection in vitro and in vivo. Adv. Funct. Mater..

[58] Guo F.Y., Chen Q.P., Liang Q., Zhang M., Chen W.X., Chen H.M., Yun Y.H., Zhong Q.P., Chen W.J. (2021). Antimicrobial activity and proposed action mechanism of linalool against *Pseudomonas fluorescens*. Front. Microbiol..

[59] Yang X., Syed R., Fang B., Zhou C.H. (2022). A new discovery towards novel skeleton of benzimidazole-conjugated pyrimidinones as unique effective antibacterial agents. Chin. J. Chem..

[60] Yu J.H., Xu X.F., Hou W., Meng Y., Huang M.Y., Lin J., Chen W.M. (2021). Synthetic cajaninstilbene acid derivatives eradicate methicillinresistant *Staphylococcus aureus* persisters and biofilms. Eur. J. Med. Chem..

[61] Zhang P.L., Laiche M.H., Li Y.L., Gao W.W., Lin J.M., Zhou C.H. (2022). An unanticipated discovery of novel naphthalimidopropanediols as potential broad-spectrum antibacterial members. Eur. J. Med. Chem..

[62] Vigyasa S., Anirban P., Mahendra P.D. (2015). A polyphenolic flavonoid glabridin: Oxidative stress response in multidrug-resistant *Staphylococcus aureus*. Free Radical Bio. Med..

[63] Zhang P.L., Gopala L., Yu Y., Fang B., Zhou C.H. (2021). Identification of a novel antifungal backbone of naphthalimide thiazoles with synergistic potential for chemical and dynamic treatment. Future Med. Chem..

[64] Yagya P.S., Paul R., Michelle G., Jon Y.T., Cheng-Wei T.C. (2019). Development of fungal selective amphiphilic kanamycin: Cost-effective synthesis and use of fluorescent analogs for mode of action investigation. ACS Infect. Dis..

[65] Deng Z., Sun H., Bheemanaboina R.R.Y., Luo Y., Zhou C.H. (2022). Natural aloe emodin-hybridized sulfonamide aminophosphates as novel potential membrane-perturbing and DNA-intercalating agents against *Enterococcus faecalis*. Bioorg. Med. Chem. Lett..

[66] Shounak R., Anupam M., Varnika Y., Ankita S., Ruptanu B., Pallab S., Amit J. (2019). Mechanistic insight into the antibacterial activity of chitosan exfoliated MOS_2_ nanosheets: Membrane damage, metabolic inactivation, and oxidative stress. ACS Appl. Bio Mater..

[67] Wang J., Ansari M.F., Zhou C.H. (2021). Unique para-aminobenzenesulfonyl oxadiazoles as novel structural potential membrane active antibacterial agents towards drug-resistant methicillin resistant *Staphylococcus aureus*. Bioorg. Med. Chem. Lett..

[68] Sun H., Li Z.Z., Jeyakkumar P., Zang Z.L., Fang B., Zhou C.H. (2022). A new discovery of unique 13-(benzimidazolylmethyl)berberines as promising broad-spectrum antibacterial agents. J. Agric. Food Chem..

[69] Zhang J., Battini N., Ou J.M., Zhang S.L., Zhang L., Zhou C.H. (2023). New efforts toward aminothiazolylquinolones with multitargeting antibacterial potential. J. Agric. Food Chem..

[70] Zhang P.L., Tangadanchu V.K.R., Zhou C.H. (2022). Identification of novel antifungal skeleton of hydroxyethy naphthalimides with synergistic potential for chemical and dynamic treatments. Molecules.

[71] Zhang W.J., Li Z., Zhou M., Wu F., Hou X.Y., Luo H., Liu H., Han X., Yan G.Y., Ding Z.Y. (2014). Synthesis and biological evaluation of 4-(1,2,3-triazol-1-yl)coumarin derivatives as potential antitumor agents. Bioorg. Med. Chem. Lett..

