# Supplementary material for: Synthesis and Biological Evaluation of Piperazine Hybridized Coumarin Indolylcyanoenones with Antibacterial Potential
Source: Molecules. 2023 Mar 9;28(6):2511. doi: 10.3390/molecules28062511 (PMC10056909; doi:10.3390/molecules28062511)
Supplement: Supplementary file 1 [file molecules-28-02511-s001.zip › molecules-2210282-supplementary.pdf]

1. The molecular docking results

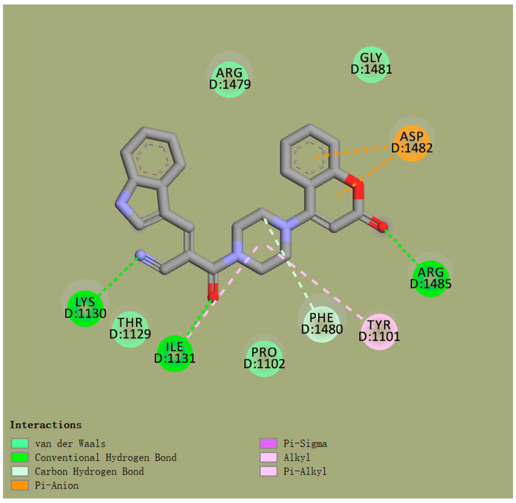

Figure S1. 2D image of DNA gyrase docked with **5a**

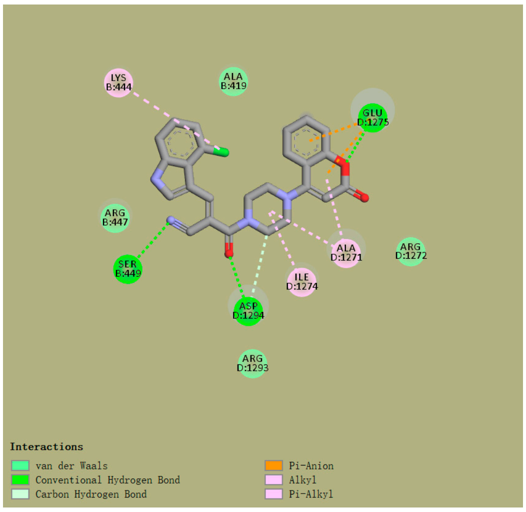

Figure S2. 2D image of DNA gyrase docked with **5b**

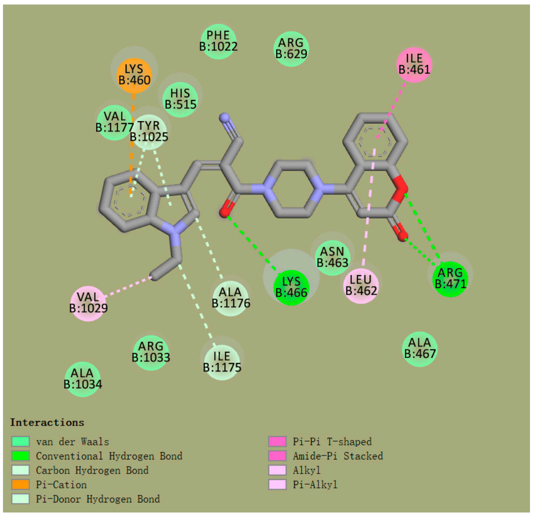

Figure S3. 2D image of DNA gyrase docked with **7a**

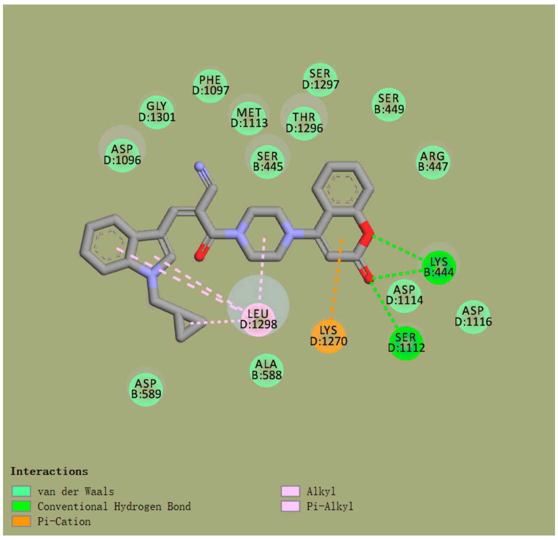

Figure S4. 2D image of DNA gyrase docked with **9a**

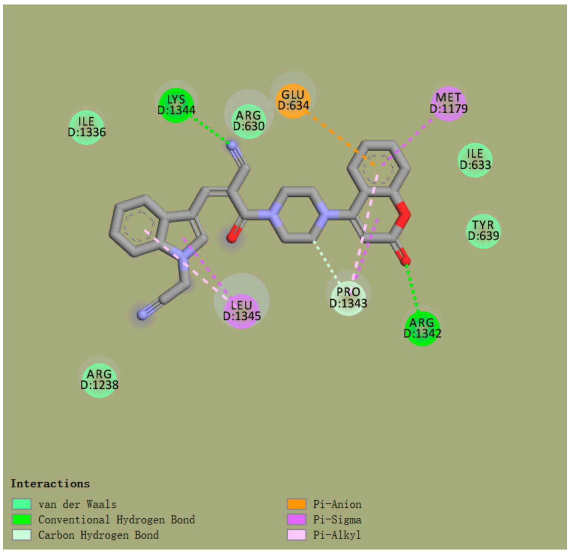

Figure S5. 2D image of DNA gyrase docked with **13a**

2. Characterizations of some representative compounds

2.1. Spectra of compound **3**

^{1}H NMR spectrum (600 MHz, 25 °C, DMSO-d_6)

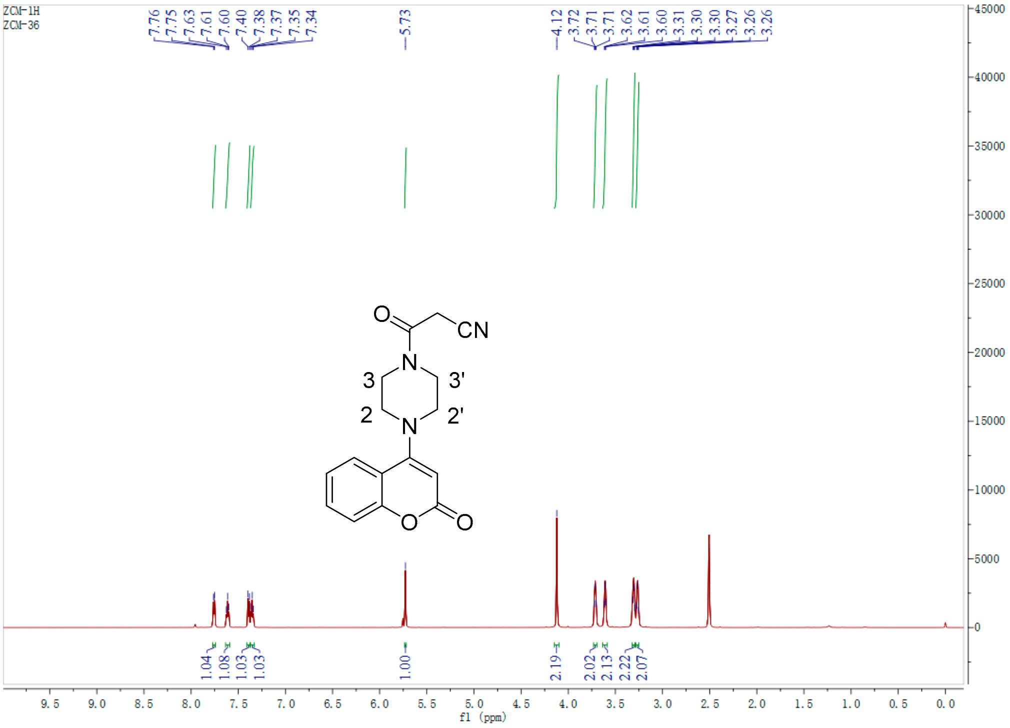

HRMS spectrum

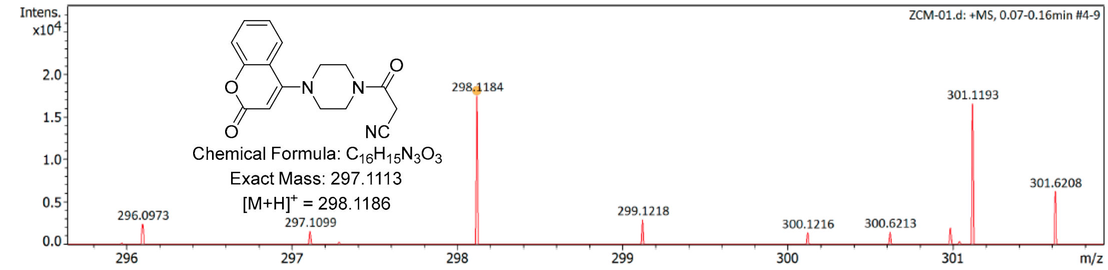

Meas. m/z	#	Ion Formula	Sum Formula	Adduct	m/z	err [ppm]	mSigma	# mSigma	Score	rdb	e¦¥ Conf	N-Rule
298.1184	1	C16H16N3O3	C16H15N3O3	M+H	298.1186	0.8	35.3	1	100.00	11.0	even	ok

2.2. Spectra of compound **5a**

^{1}H NMR spectrum (600 MHz, 25 °C, DMSO-d_6)

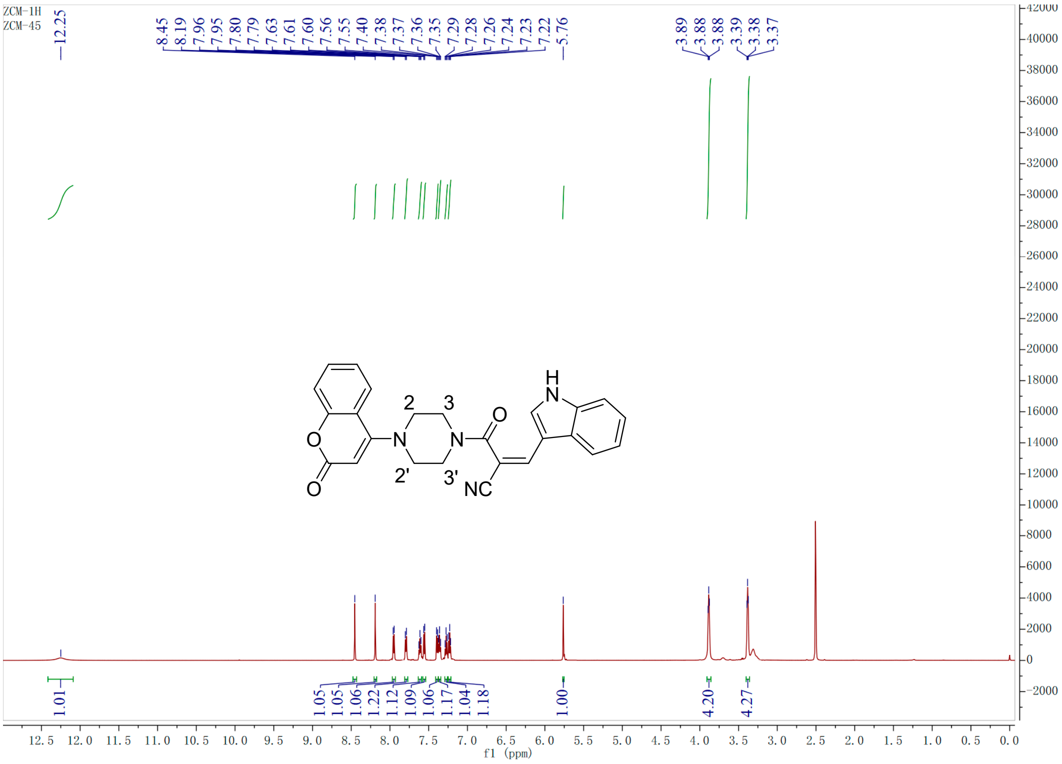

^{13}C NMR spectrum (151 MHz, 25 ºC, DMSO-d_6)

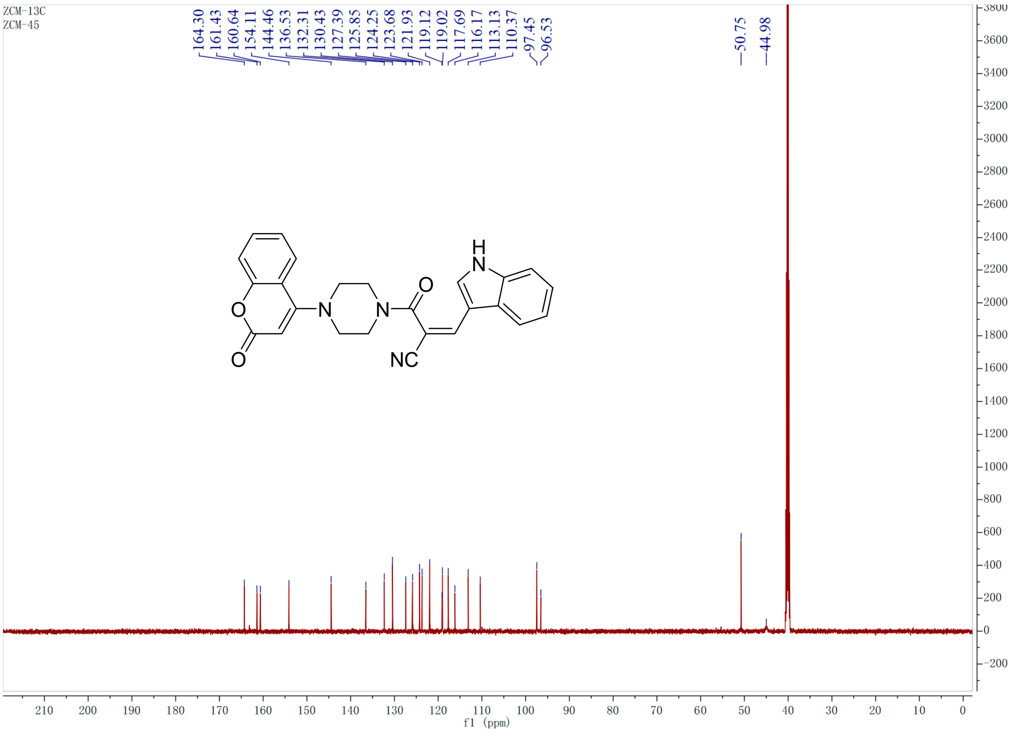

HRMS spectrum

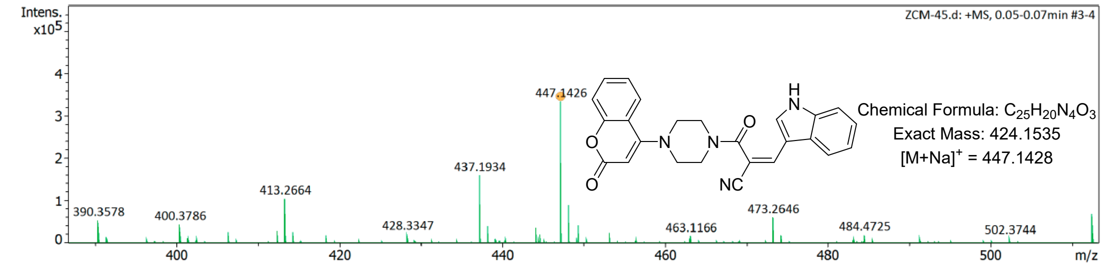

Meas. m/z	#	Ion Formula	Sum Formula	Adduct	m/z	err [ppm]	mSigma	# mSigma	Score	rdb	e¡¥ Conf	N-Rule
447.1426	1	C25H20N4NaO3	C25H20N4O3	M+Na	447.1428	0.4	12.8	1	100.00	18.0	even	ok

2.3. Spectra of compound **5b**

^{1}H NMR spectrum (600 MHz, 25 ºC, DMSO-d_6)

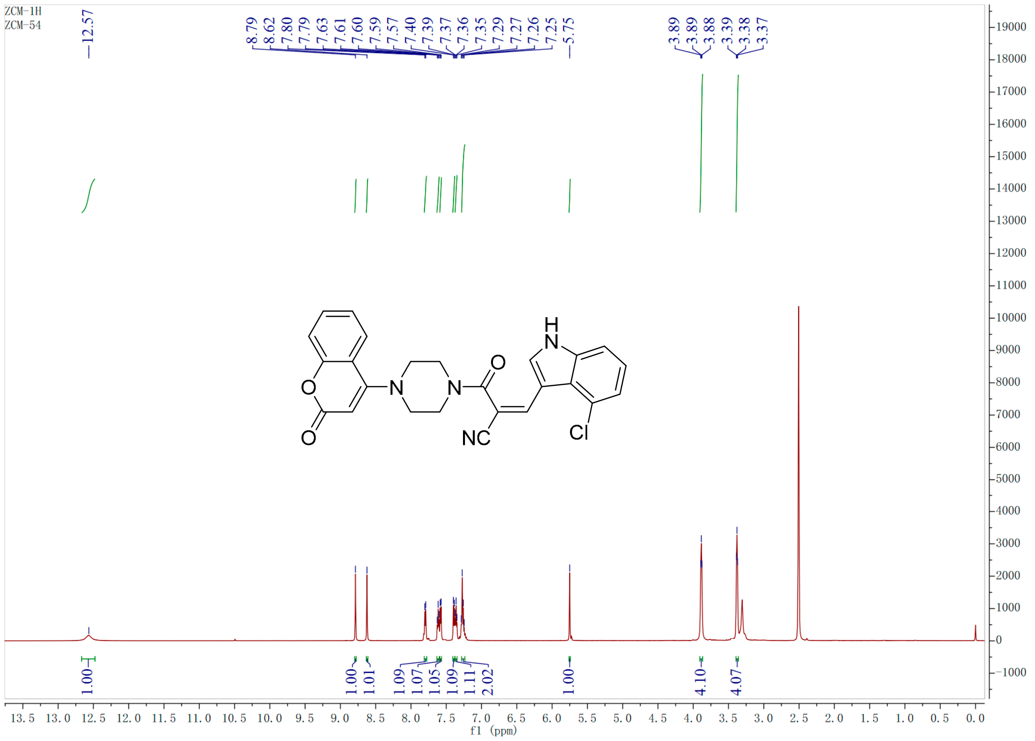

^{13}C NMR spectrum (151 MHz, 25 ºC, DMSO-d_6)

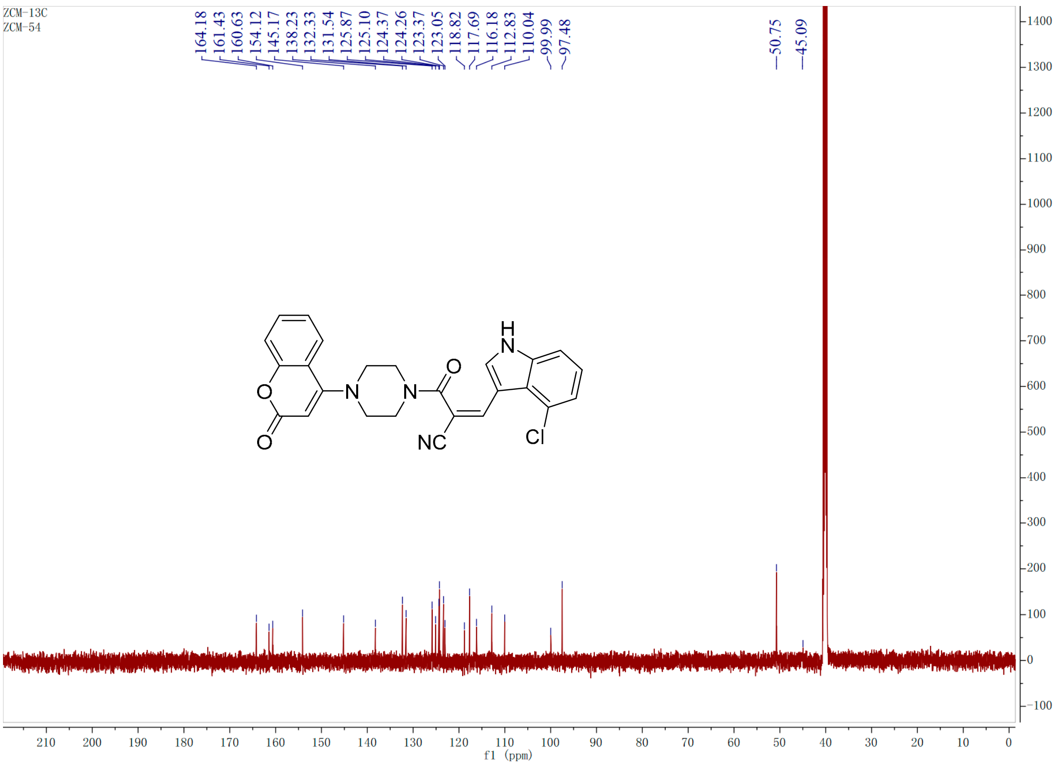

HRMS spectrum

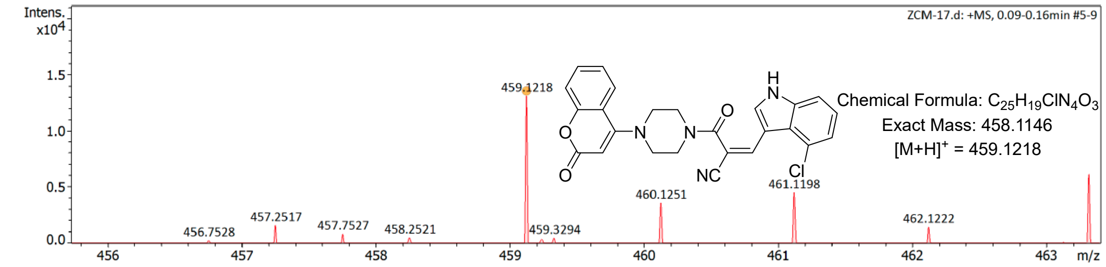

Meas. m/z	#	Ion Formula	Sum Formula	Adduct	m/z	err [ppm]	mSigma	# mSigma	Score	rdb	e¡¥ Conf	N-Rule
459.1218	1	C25H20ClN4O3	C25H19ClN4O3	M+H	459.1218	0.1	13.1	1	100.00	21.0	even	ok

2.4. Spectra of compound **5c**

^1H NMR spectrum (600 MHz, 25 °C, DMSO-d_6)

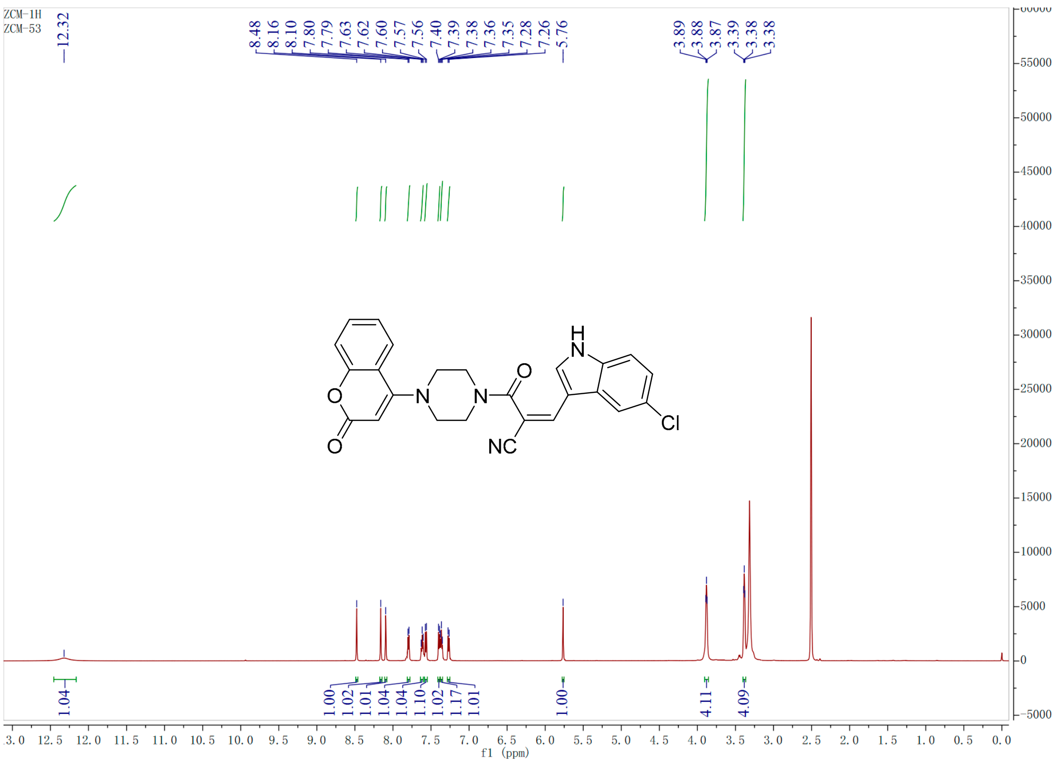

^{13}C NMR spectrum (151 MHz, 25 ºC, DMSO-d_6)

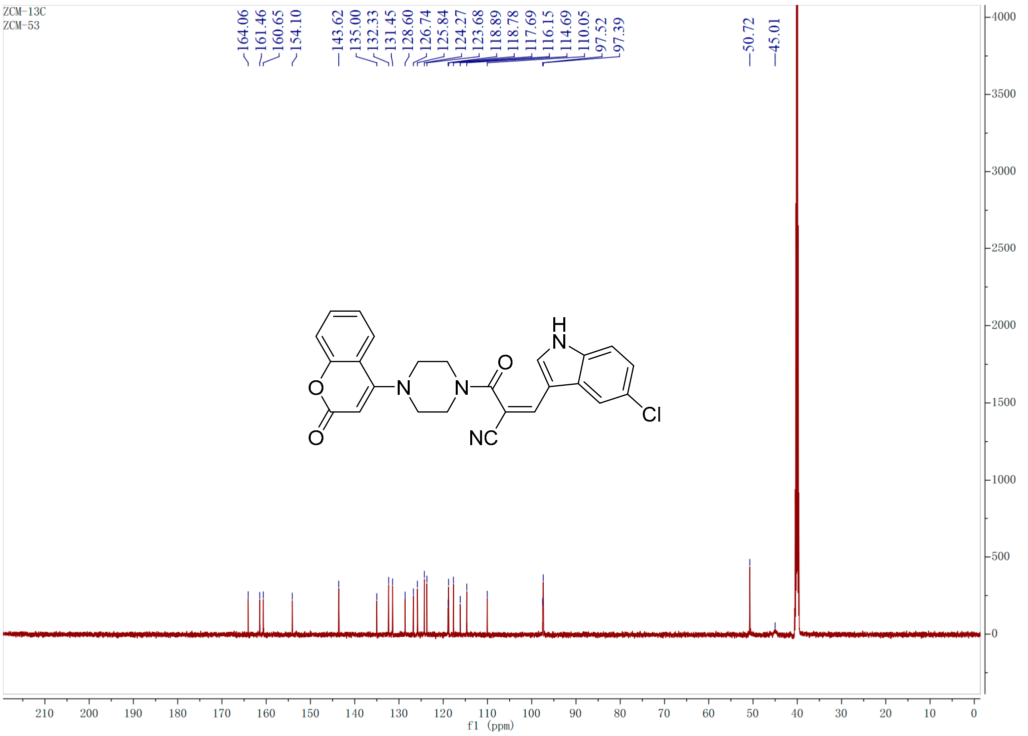

HRMS spectrum

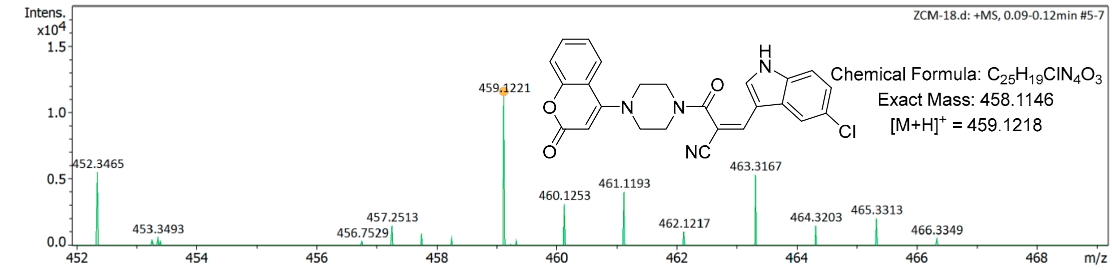

Meas. m/z	#	Ion Formula	Sum Formula	Adduct	m/z	err [ppm]	mSigma	# mSigma	Score	rdb	e¡¥ Conf	N-Rule
459.1221	1	C25H20ClN4O3	C25H19ClN4O3	M+H	459.1218	-0.5	9.2	1	100.00	21.0	even	ok

2.5. Spectra of compound **5d**

^{1}H NMR spectrum (600 MHz, 25 ºC, DMSO-d_6)

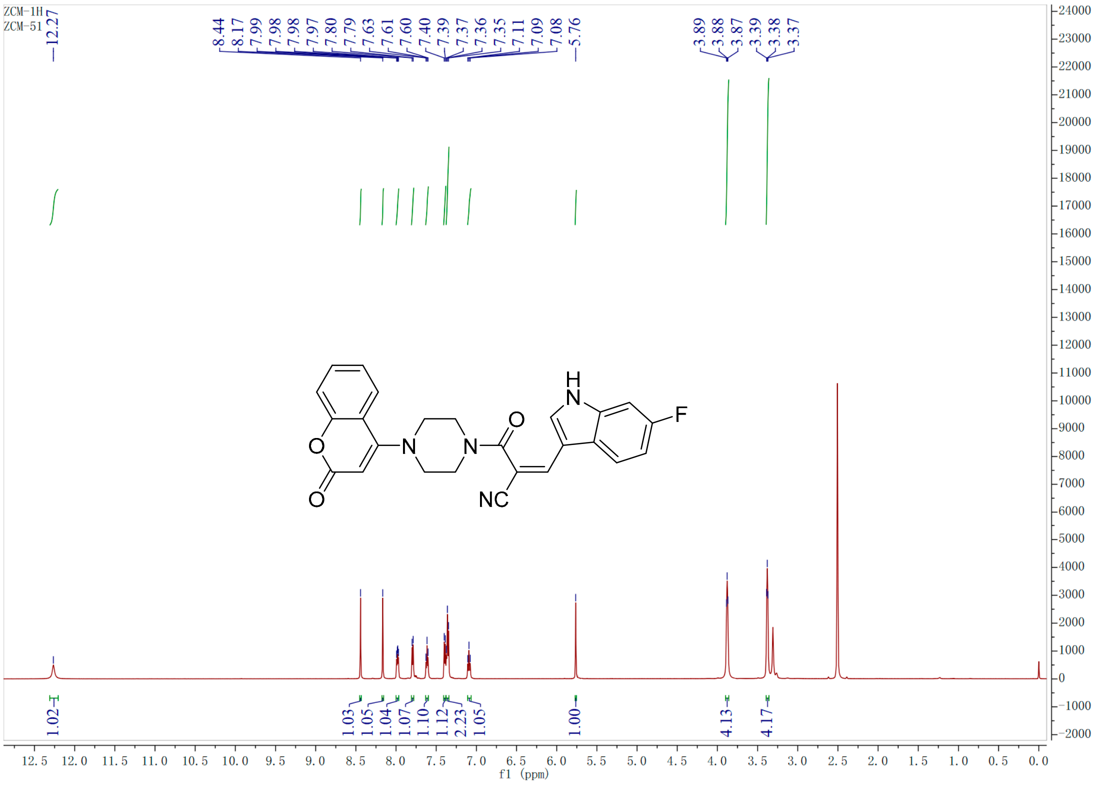

^{13}C NMR spectrum (151 MHz, 25 ºC, DMSO-d_6)

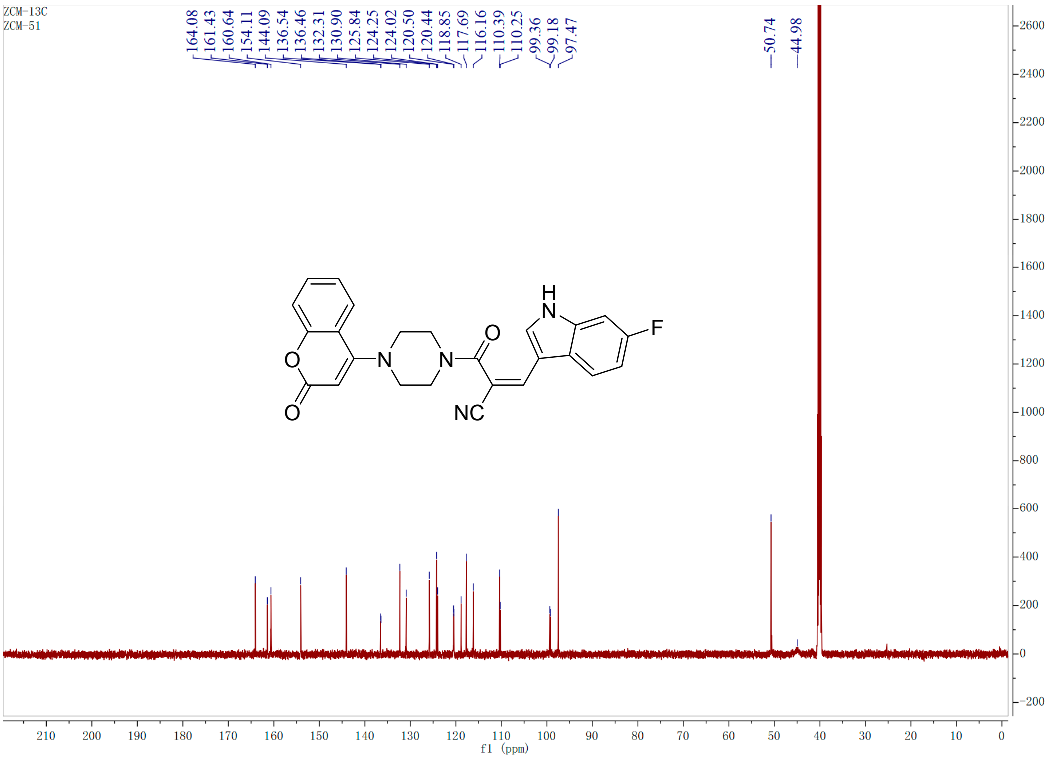

HRMS spectrum

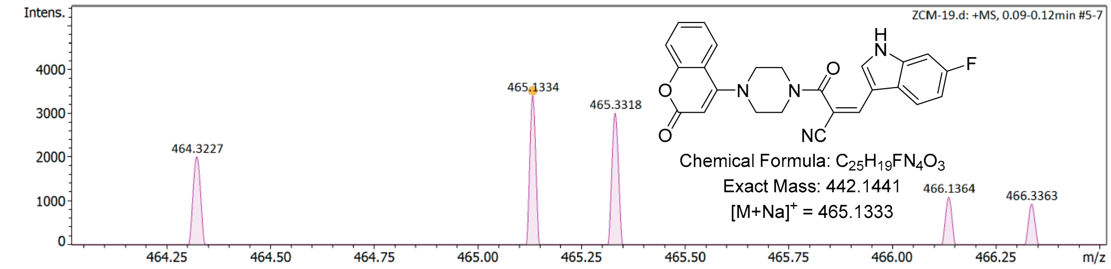

Meas. m/z	#	Ion Formula	Sum Formula	Adduct	m/z	err [ppm]	mSigma	# mSigma	Score	rdb	e¡¥ Conf	N-Rule
465.1334	1	C25H19FN4NaO3	C25H19FN4O3	M+Na	465.1333	-0.0	32.1	1	100.00	18.0	even	ok

2.6. Spectra of compound **5e**

^{1}H NMR spectrum (600 MHz, 25 °C, DMSO-d_6)

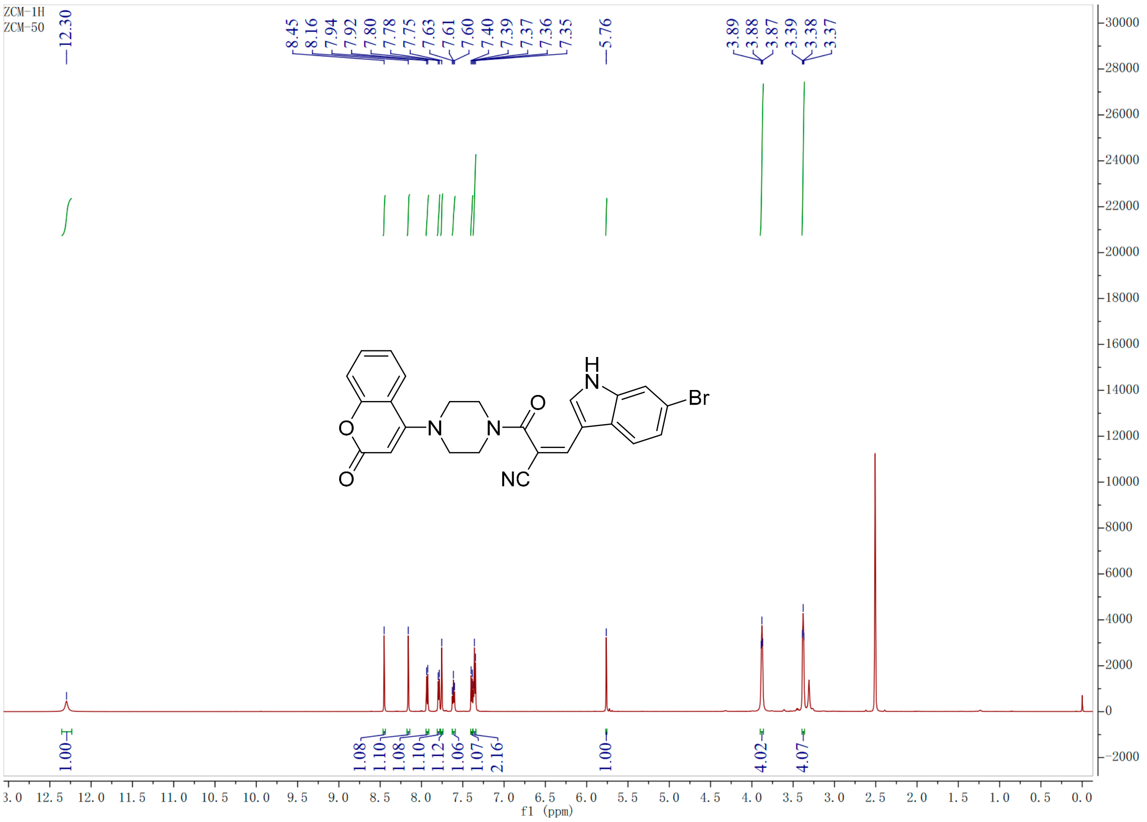

^{13}C NMR spectrum (151 MHz, 25 ºC, DMSO-d_6)

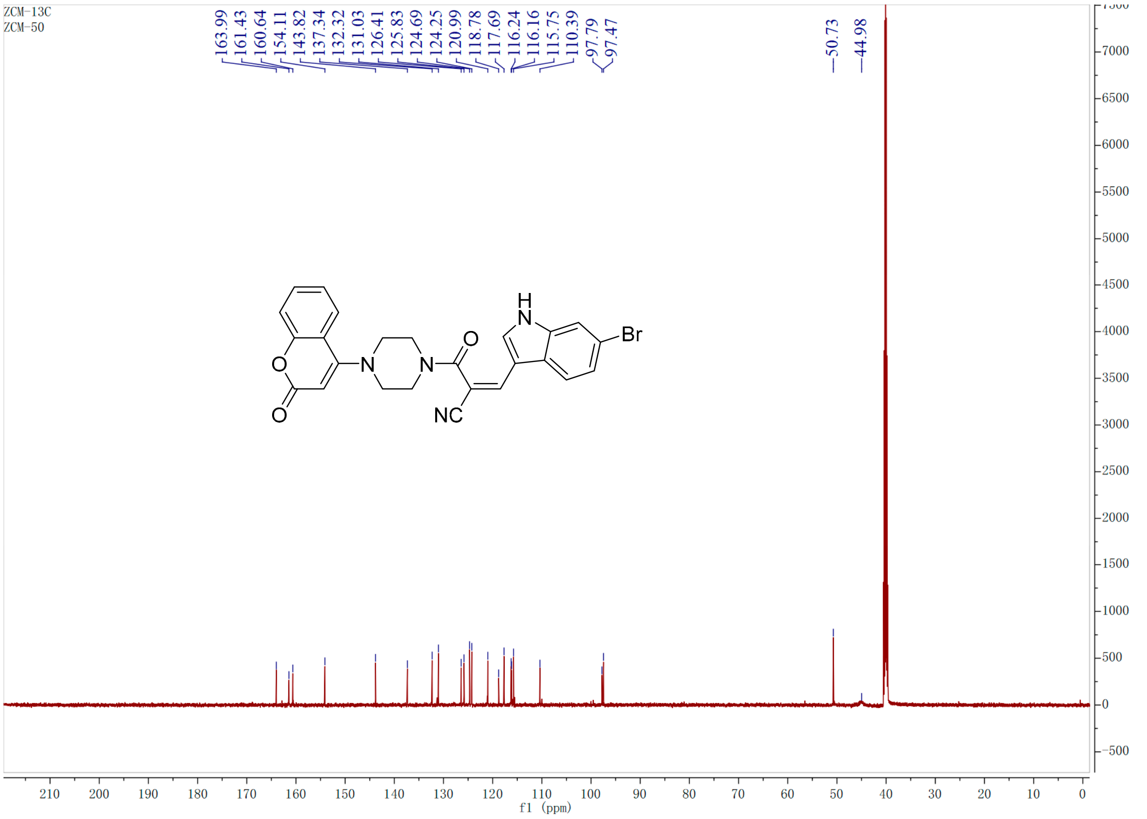

HRMS spectrum

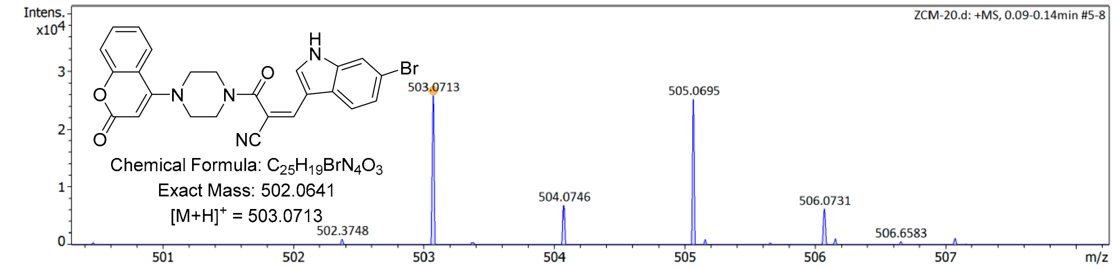

Meas. m/z	#	Ion Formula	Sum Formula	Adduct	m/z	err [ppm]	mSigma	# mSigma	Score	rdb	e¡¥ Conf	N-Rule
503.0713	1	C25H20BrN4O3	C25H19BrN4O3	M+H	503.0713	0.0	23.4	1	100.00	20.0	even	ok

2.7. Spectra of compound **5f**

^{1}H NMR spectrum (600 MHz, 25 ºC, DMSO-d_6)

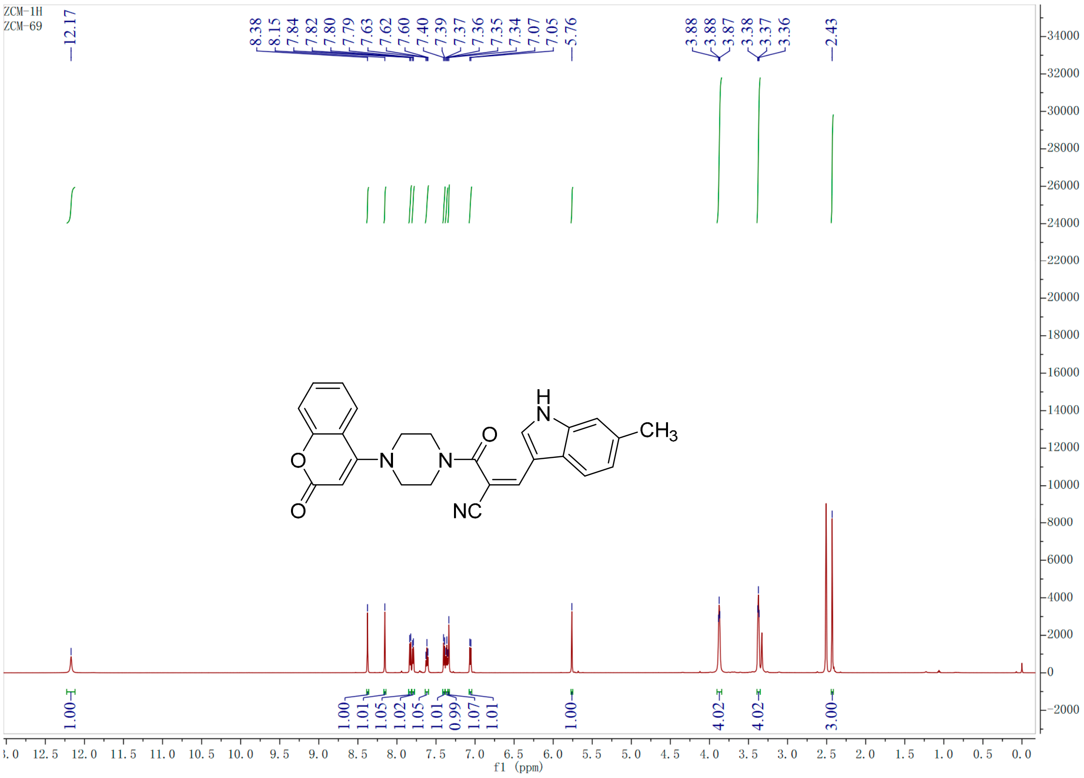

^{13}C NMR spectrum (151 MHz, 25 ºC, DMSO-d_6)

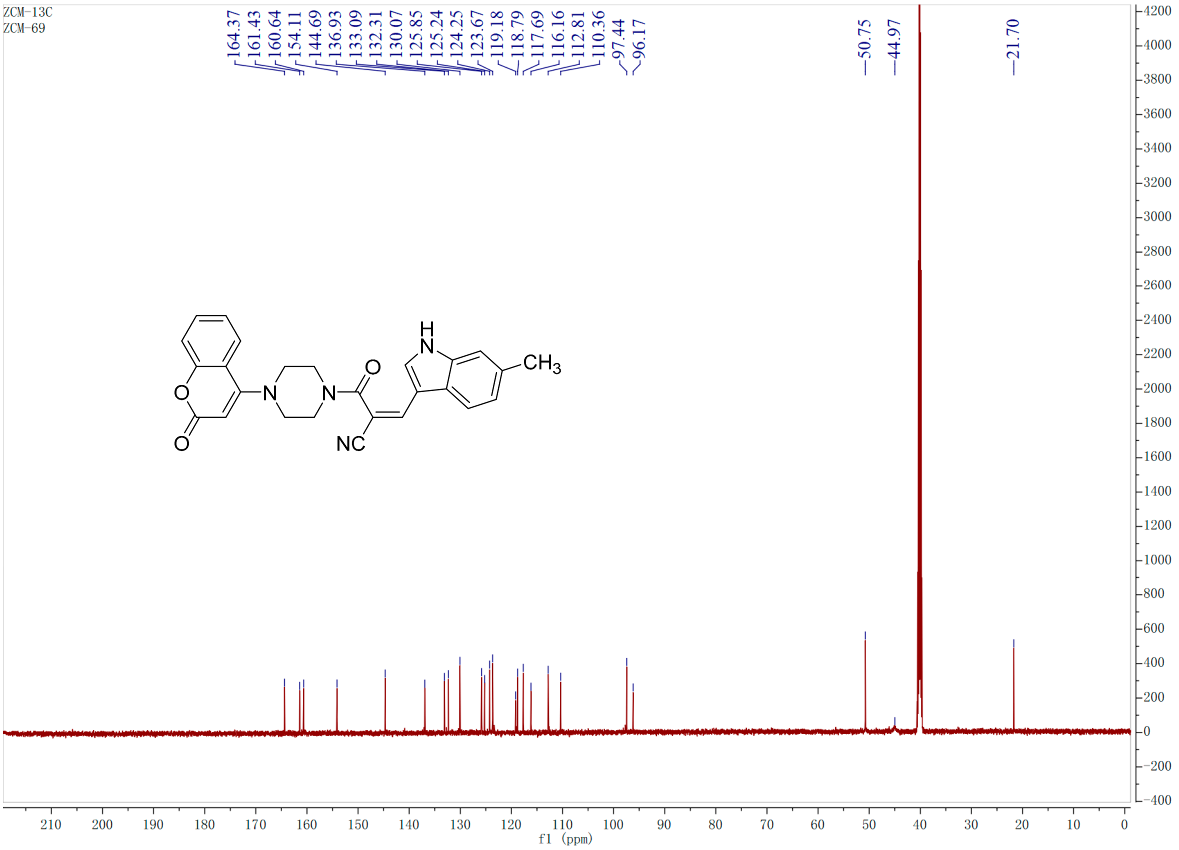

HRMS spectrum

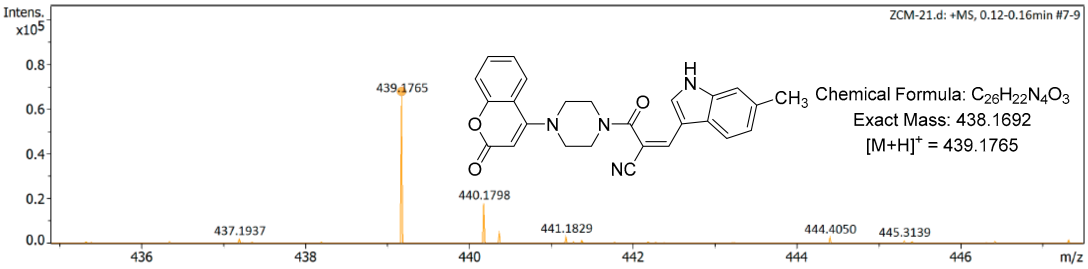

Meas. m/z	#	Ion Formula	Sum Formula	Adduct	m/z	err [ppm]	mSigma	# mSigma	Score	rdb	e¡¥ Conf	N-Rule
439.1765	1	C26H23N4O3	C26H22N4O3	M+H	439.1765	0.0	15.4	1	100.00	18.0	even	ok

2.8. Spectra of compound **5g**

^{1}H NMR spectrum (600 MHz, 25 °C, DMSO-*d*$_6$)

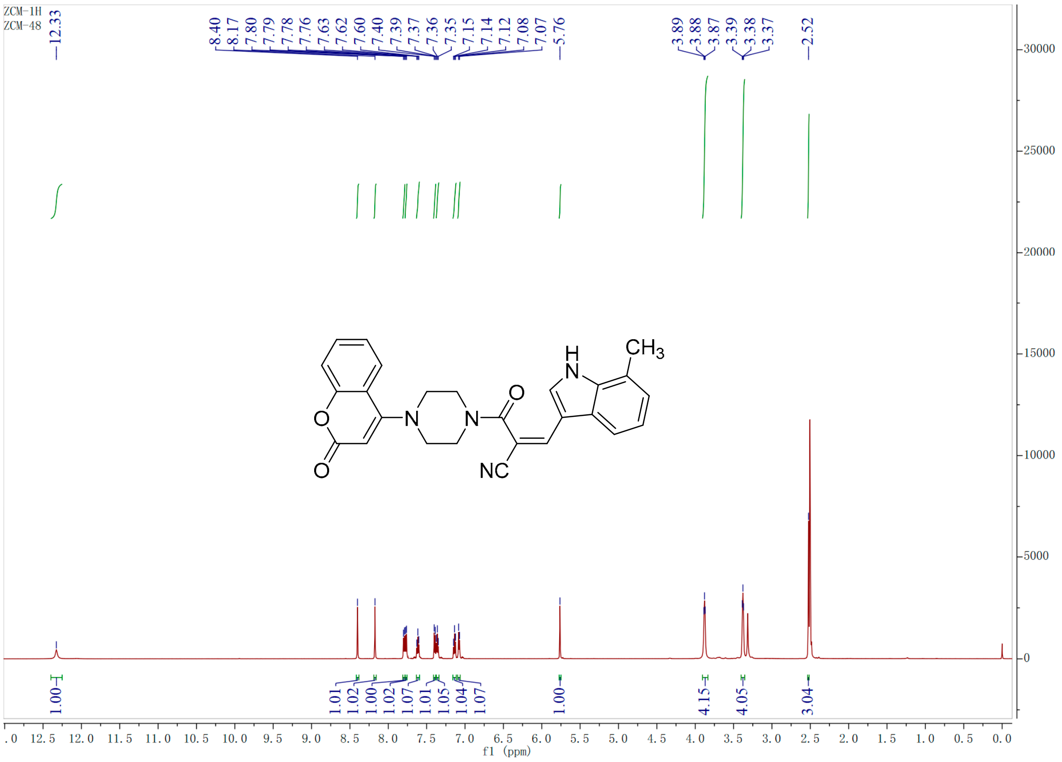

^{13}C NMR spectrum (151 MHz, 25 ºC, DMSO-d_6)

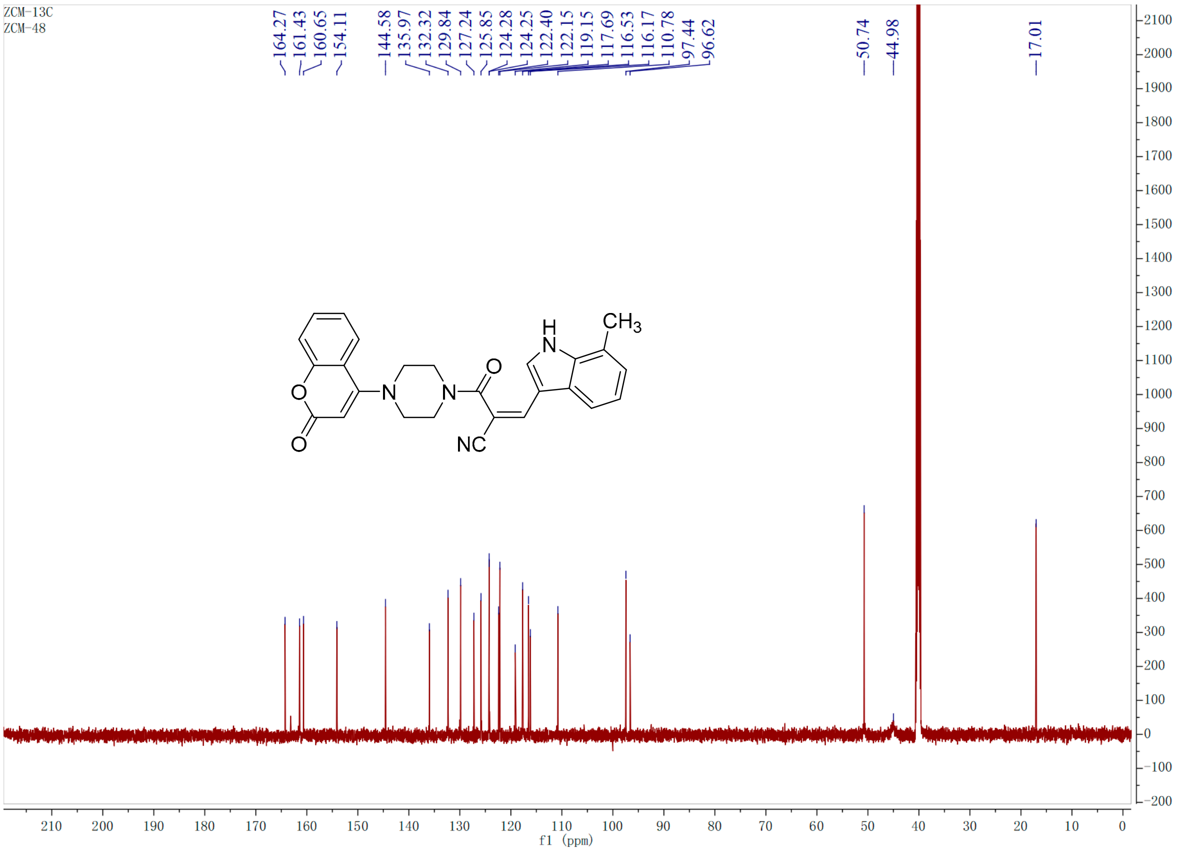

HRMS spectrum

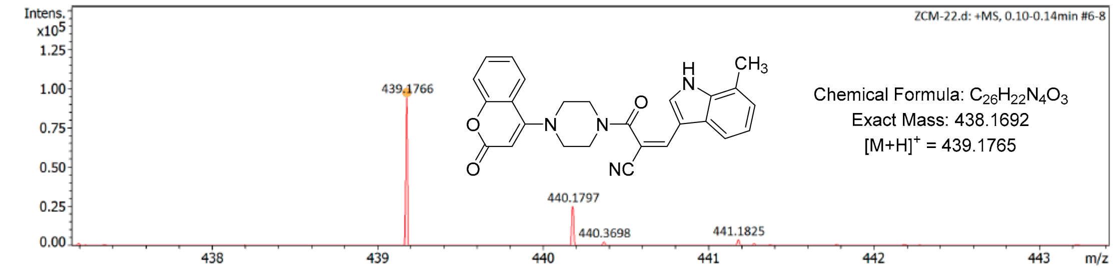

Meas. m/z	#	Ion Formula	Sum Formula	Adduct	m/z	err [ppm]	mSigma	# mSigma	Score	rdb	e¡¥ Conf	N-Rule
439.1766	1	C26H23N4O3	C26H22N4O3	M+H	439.1765	-0.4	19.0	1	100.00	18.0	even	ok

2.9. Spectra of compound **7a**

^{1}H NMR spectrum (600 MHz, 25 ºC, DMSO-d_6)

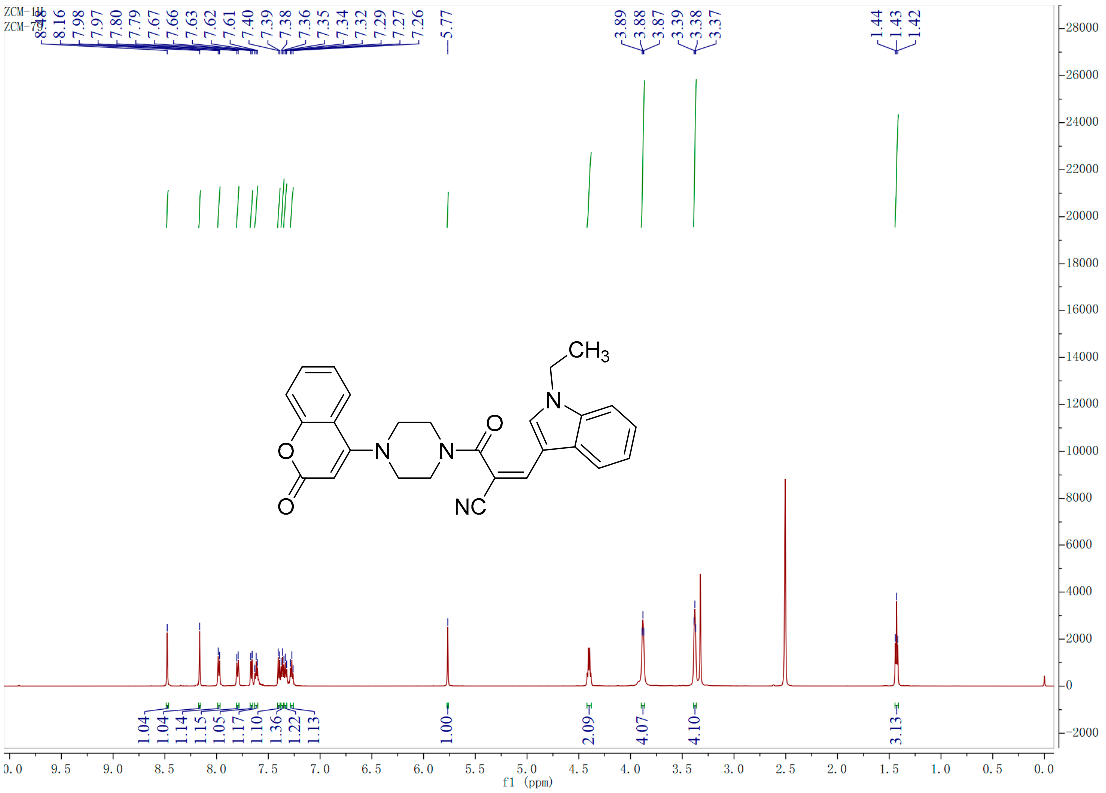

^{13}C NMR spectrum (151 MHz, 25 ºC, DMSO-d_6)

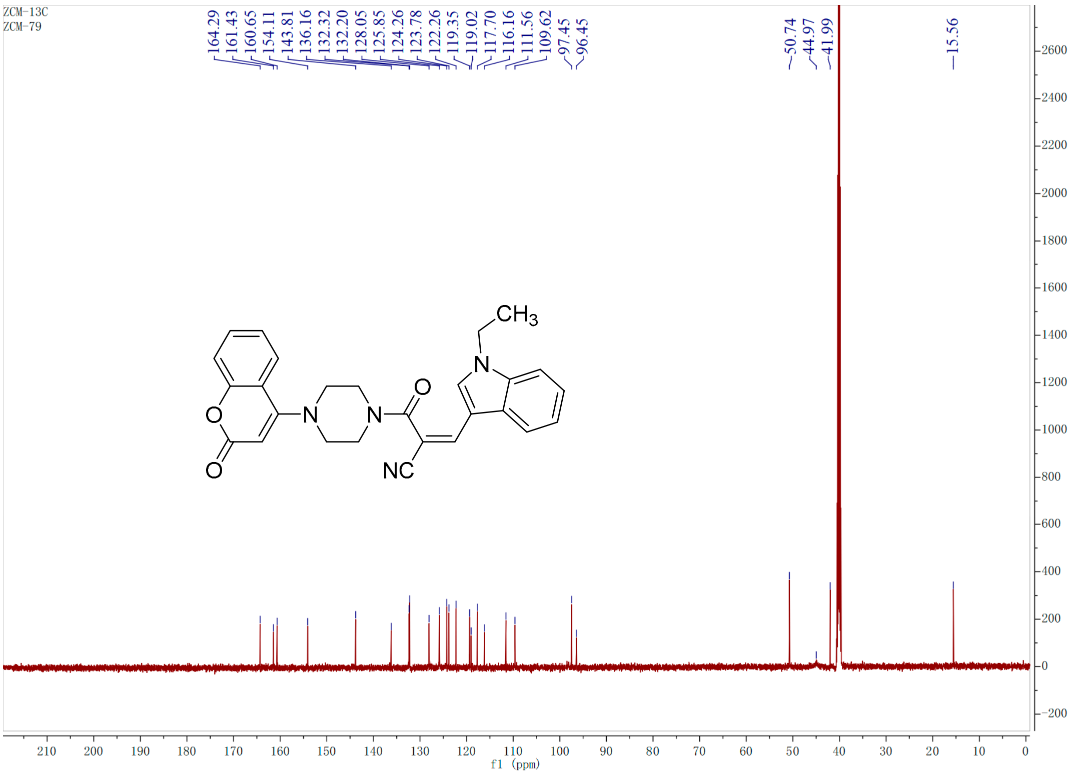

HRMS spectrum

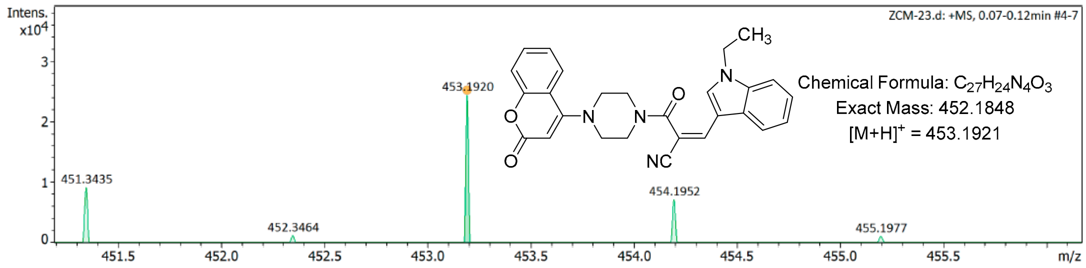

Meas. m/z	#	Ion Formula	Sum Formula	Adduct	m/z	err [ppm]	mSigma	# mSigma	Score	rdb	e¦¥ Conf	N-Rule
453.1920	1	C27H25N4O3	C27H24N4O3	M+H	453.1921	0.2	10.6	1	100.00	18.0	even	ok

2.10. Spectra of compound **7b**

^{1}H NMR spectrum (600 MHz, 25 °C, DMSO-d_6)

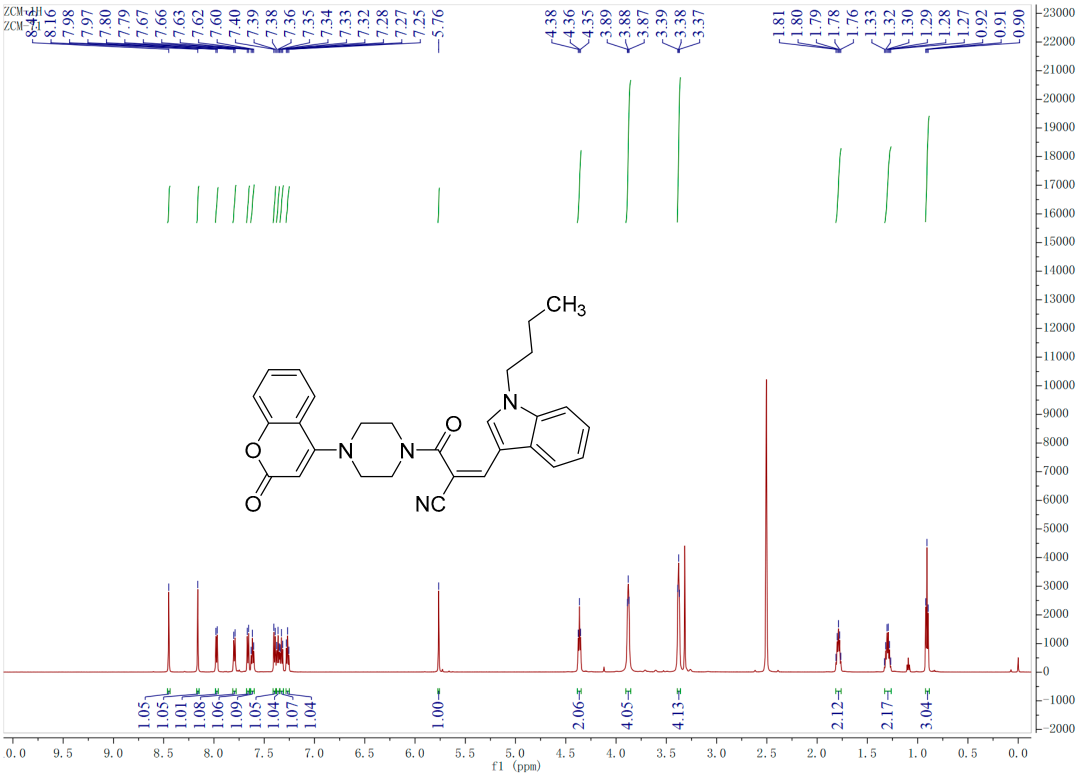

^{13}C NMR spectrum (151 MHz, 25 ºC, DMSO-d_6)

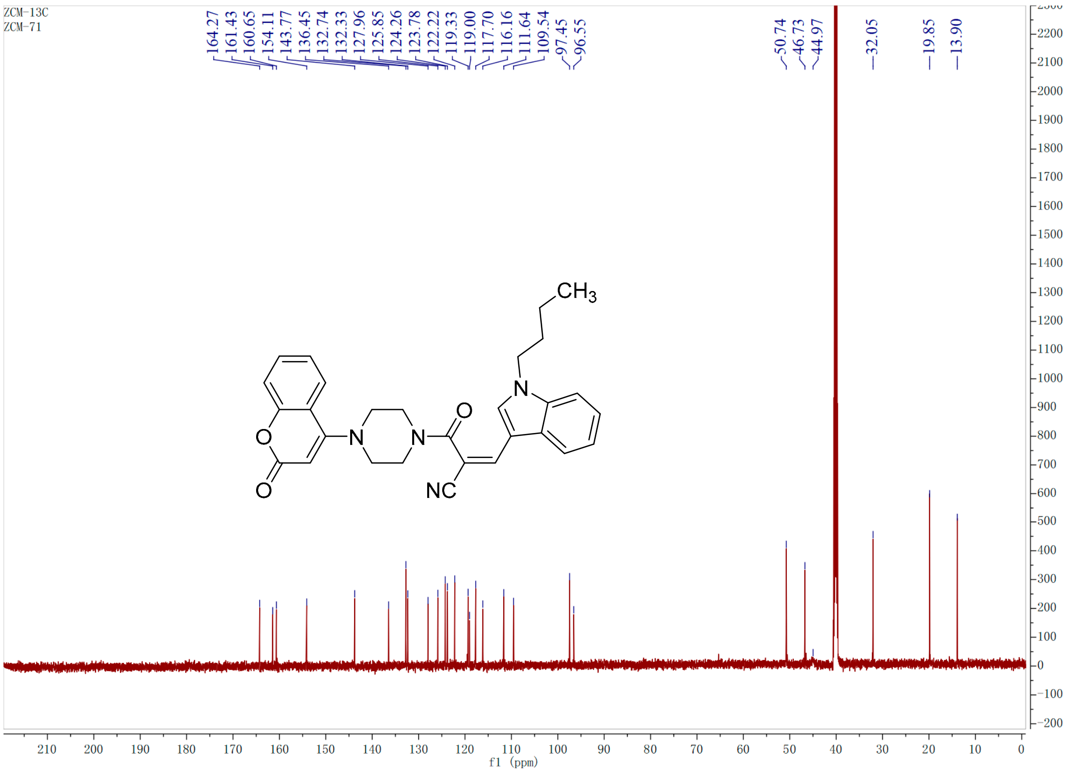

HRMS spectrum

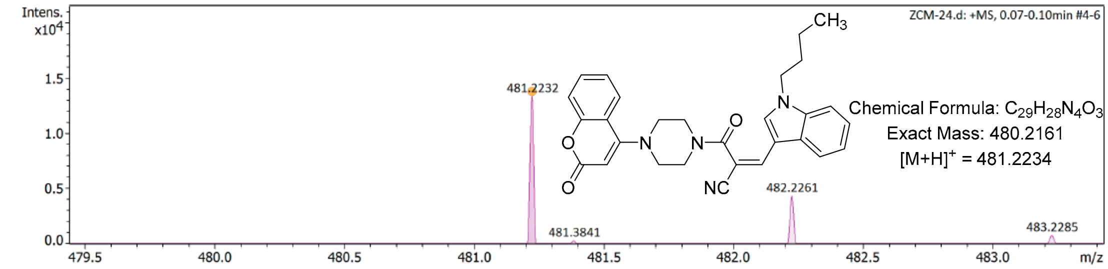

Meas. m/z	#	Ion Formula	Sum Formula	Adduct	m/z	err [ppm]	mSigma	# mSigma	Score	rdb	e¦¥ Conf	N-Rule
481.2232	1	C29H29N4O3	C29H28N4O3	M+H	481.2234	0.4	5.5	1	100.00	18.0	even	ok

2.11. Spectra of compound **7c**

^{1}H NMR spectrum (600 MHz, 25 ºC, DMSO-d_6)

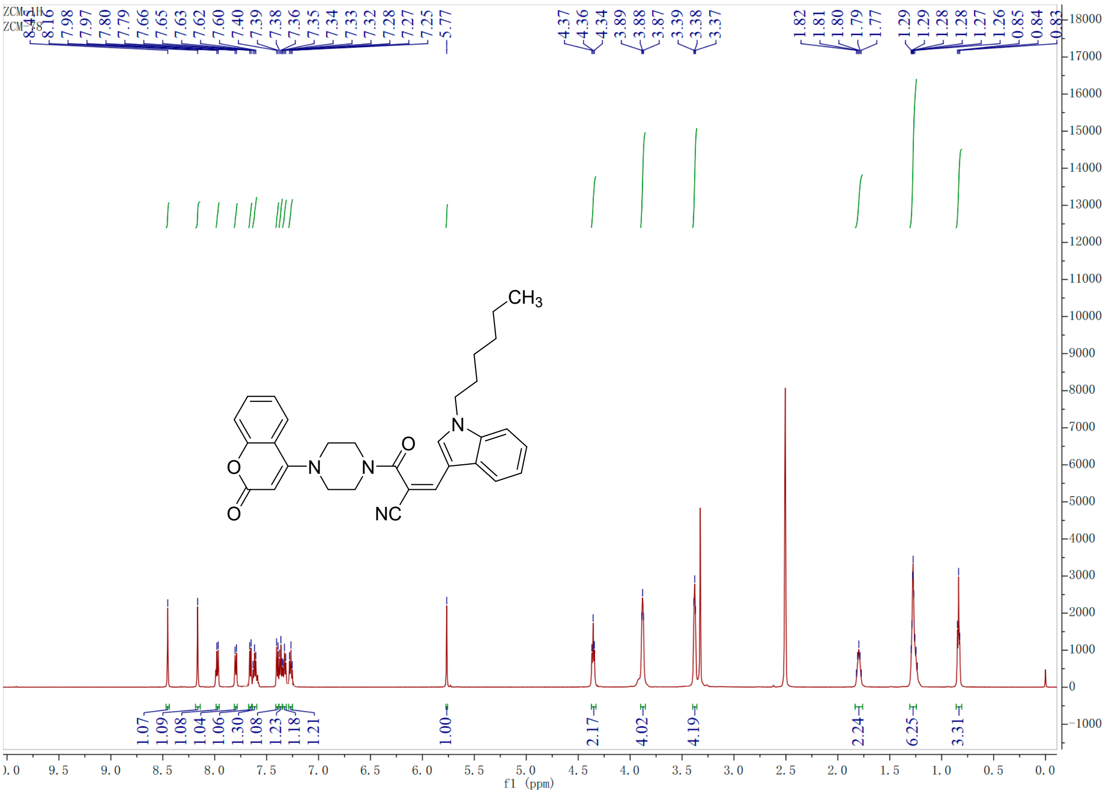

^{13}C NMR spectrum (151 MHz, 25 ºC, DMSO-d_6)

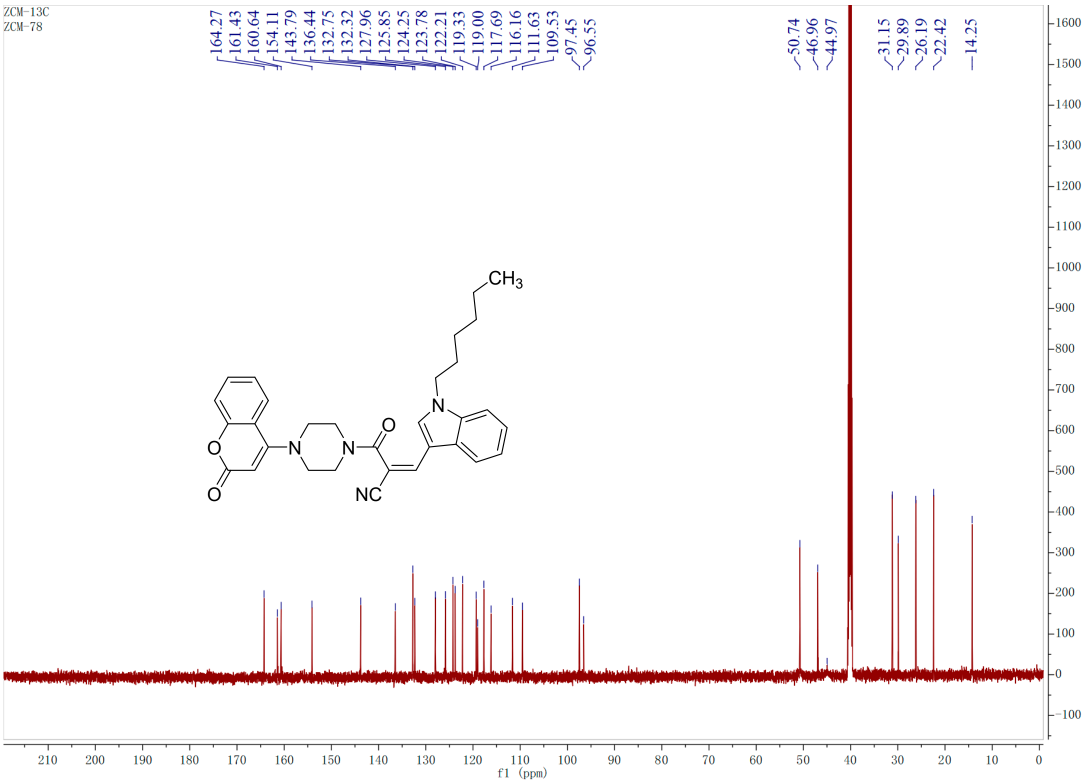

HRMS spectrum

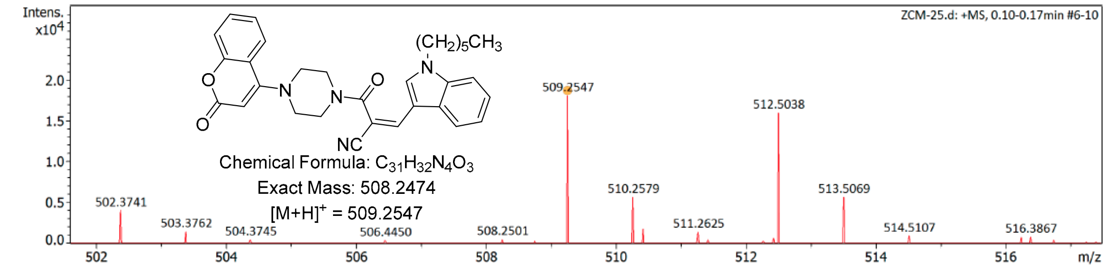

Meas. m/z	#	Ion Formula	Sum Formula	Adduct	m/z	err [ppm]	mSigma	# mSigma	Score	rdb	e¦¥ Conf	N-Rule
509.2547	1	C31H33N4O3	C31H32N4O3	M+H	509.2547	-0.0	20.7	1	100.00	18.0	even	ok

2.12. Spectra of compound **7d**

^{1}H NMR spectrum (600 MHz, 25 °C, DMSO-d_6)

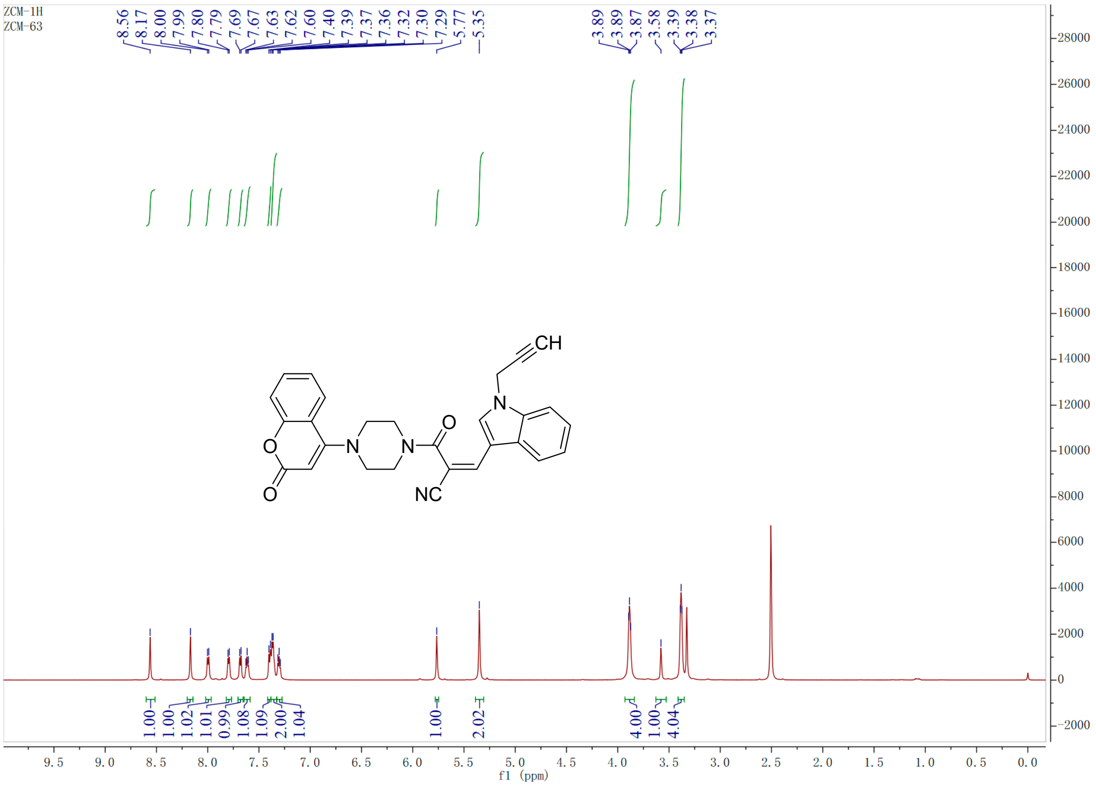

^{13}C NMR spectrum (151 MHz, 25 ºC, DMSO-d_6)

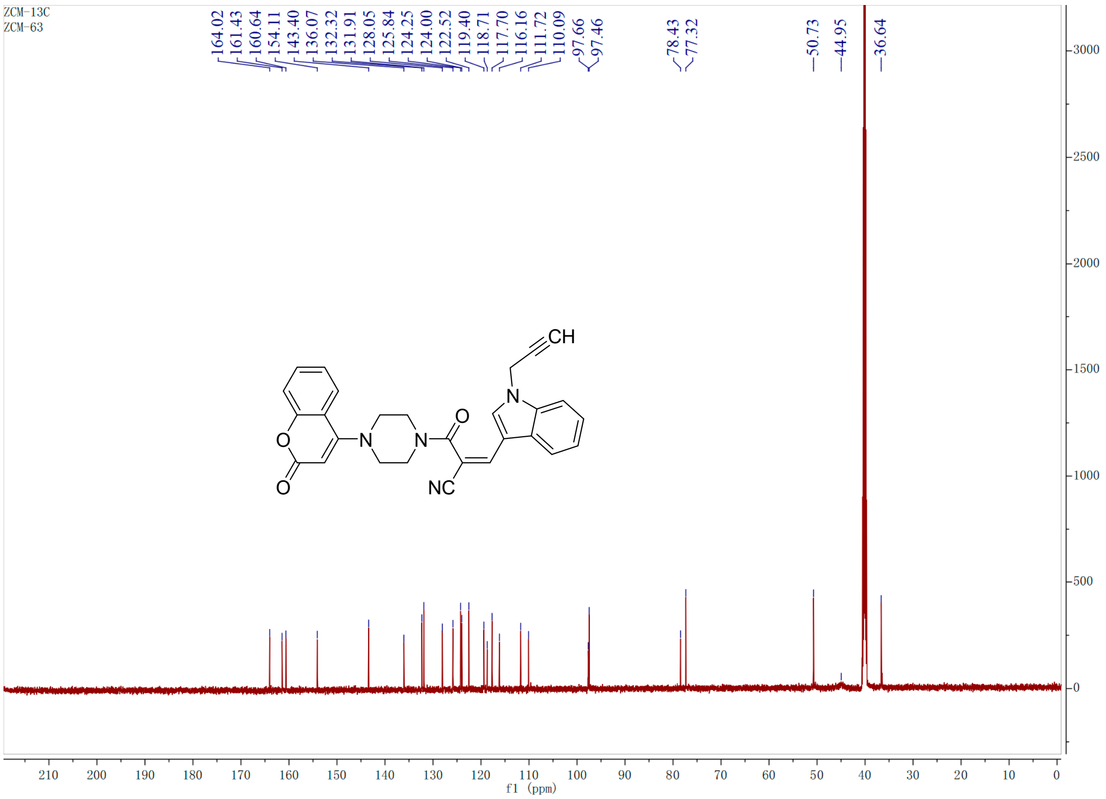

HRMS spectrum

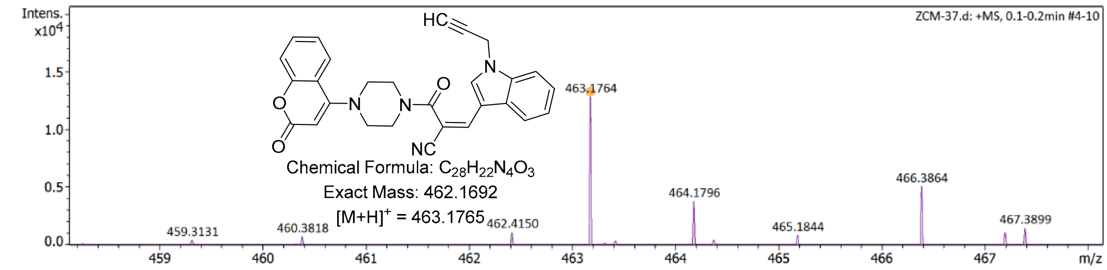

Meas. m/z	#	Ion Formula	Sum Formula	Adduct	m/z	err [ppm]	mSigma	# mSigma	Score	rdb	e¦¥ Conf	N-Rule
463.1764	1	C28H23N4O3	C28H22N4O3	M+H	463.1765	0.2	13.0	1	100.00	20.0	even	ok

2.13. Spectra of compound **7e**

^{1}H NMR spectrum (600 MHz, 25 °C, DMSO-d_6)

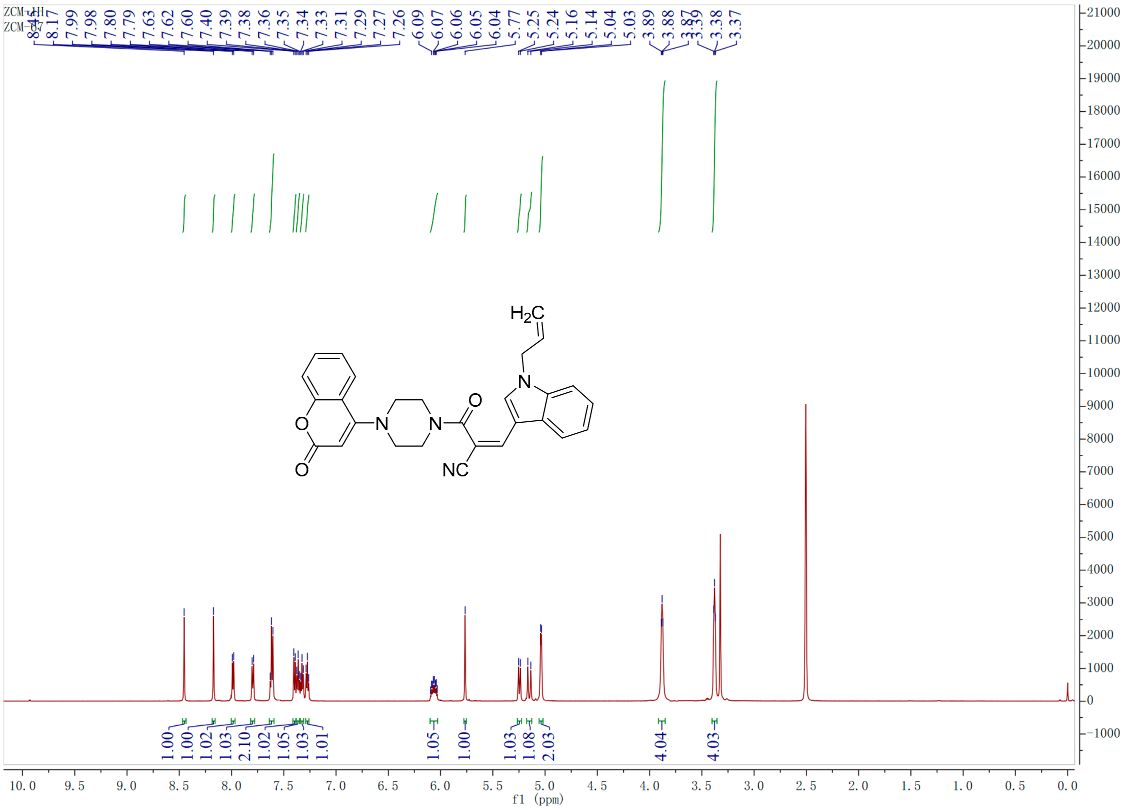

^{13}C NMR spectrum (151 MHz, 25 °C, DMSO-d_6)

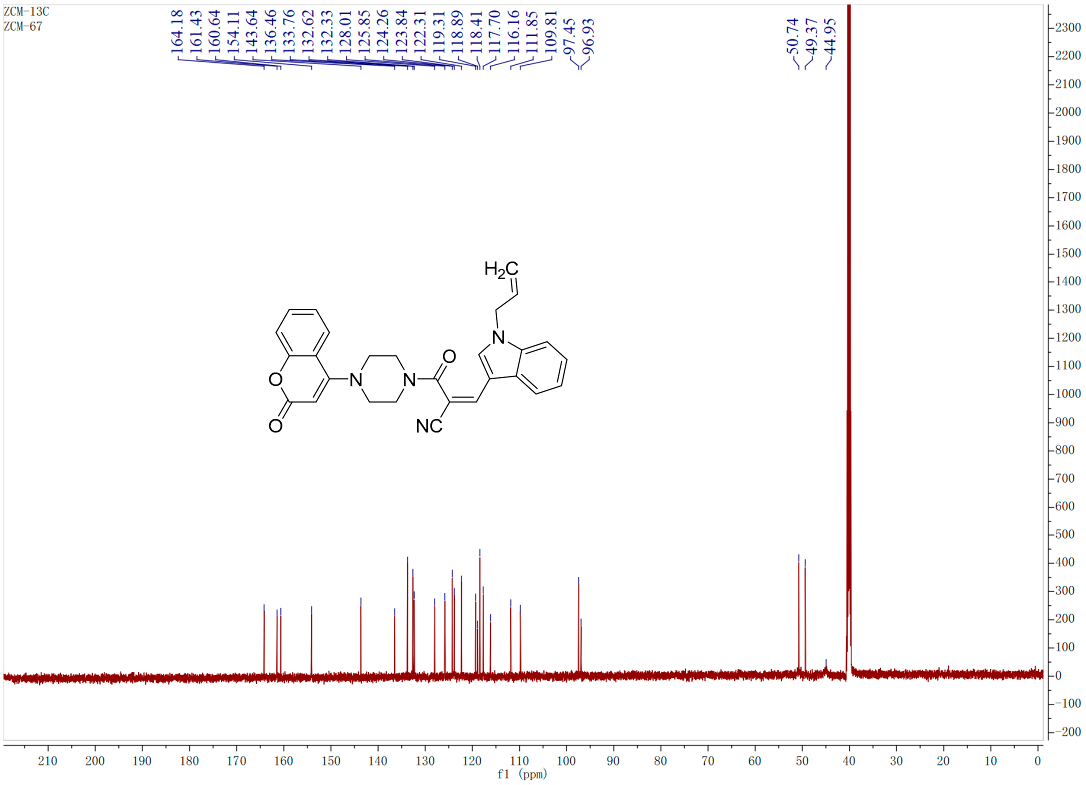

HRMS spectrum

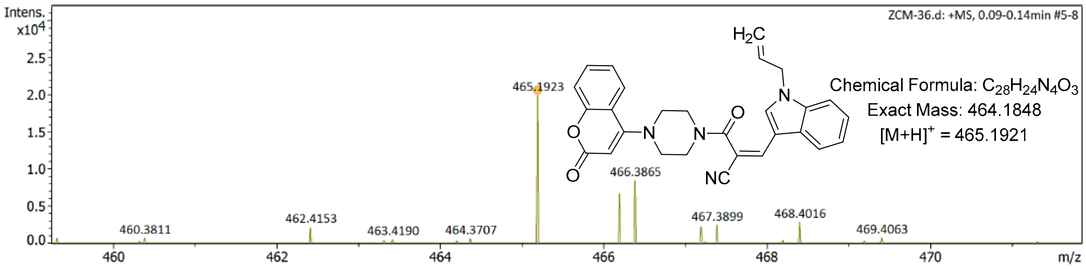

Meas. m/z	#	Ion Formula	Sum Formula	Adduct	m/z	err [ppm]	mSigma	# mSigma	Score	rdb	e¦¥ Conf	N-Rule
465.1923	1	C28H25N4O3	C28H24N4O3	M+H	465.1921	-0.3	31.5	1	100.00	19.0	even	ok

2.14. Spectra of compound **7f**

^{1}H NMR spectrum (600 MHz, 25 °C, DMSO-d_6)

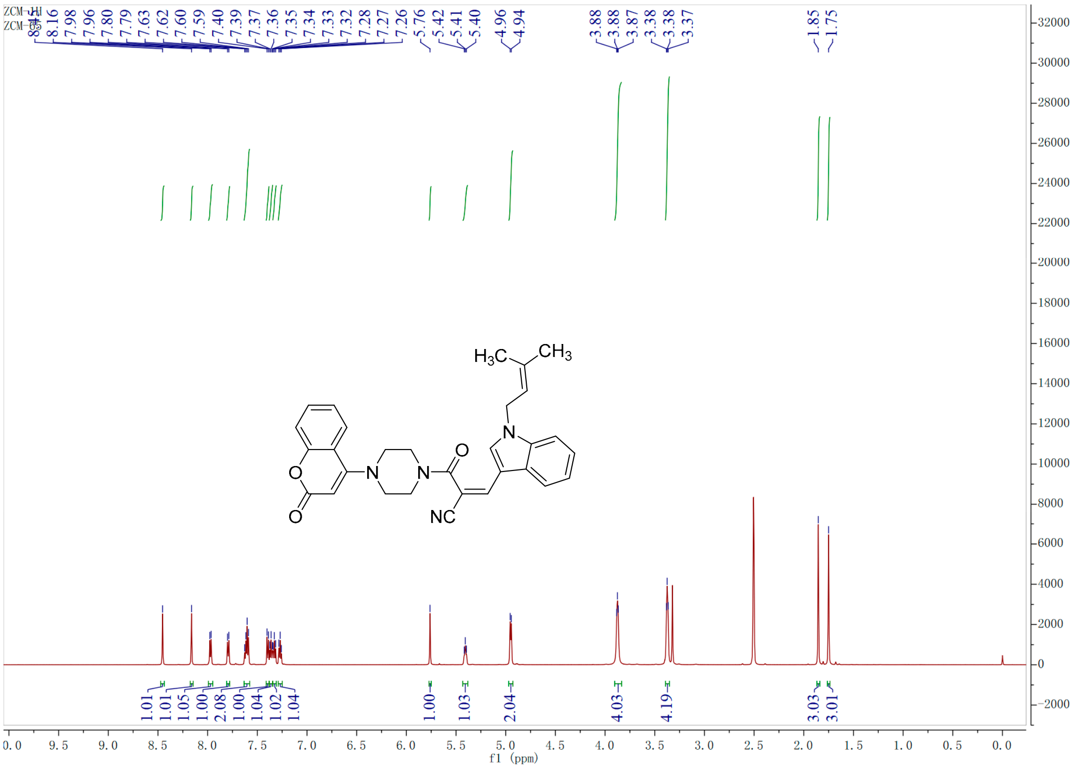

^{13}C NMR spectrum (151 MHz, 25 ºC, DMSO-d_6)

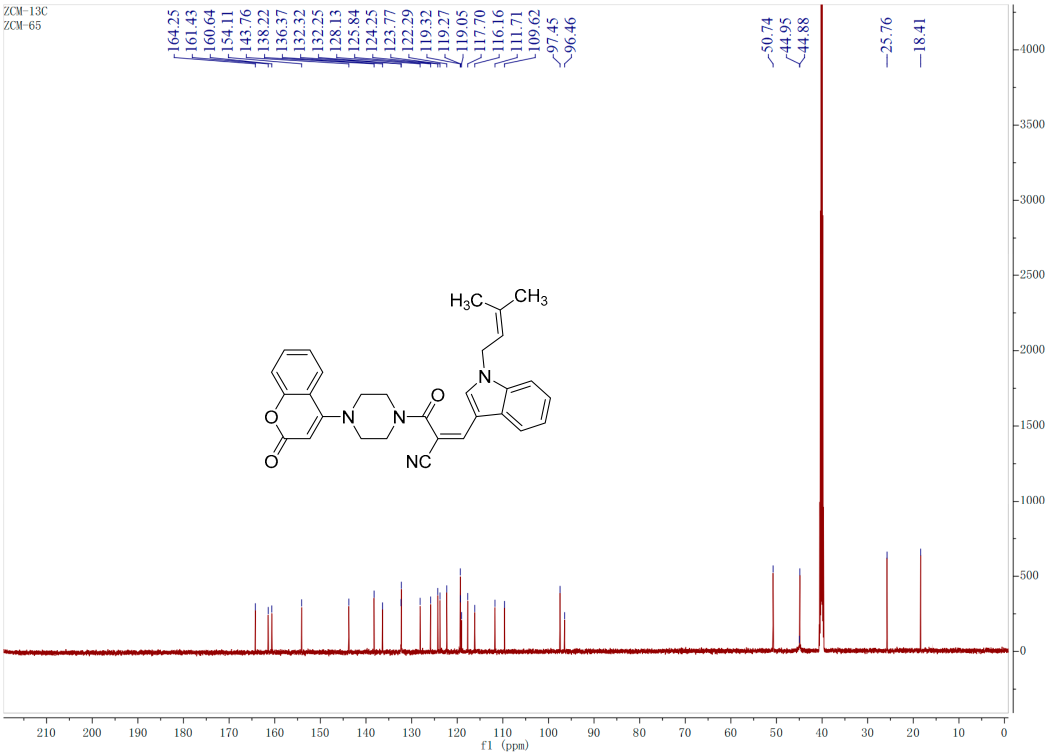

HRMS spectrum

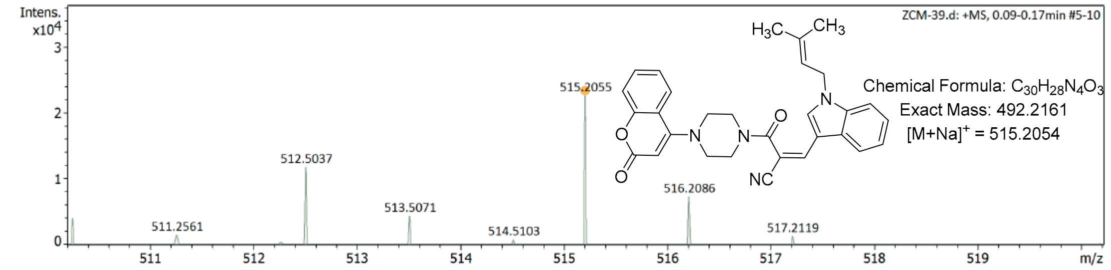

Meas. m/z	#	Ion Formula	Sum Formula	Adduct	m/z	err [ppm]	mSigma	# mSigma	Score	rdb	e¡¥ Conf	N-Rule
515.2055	1	C30H28N4NaO3	C30H28N4O3	M+Na	515.2054	-0.2	10.9	1	100.00	19.0	even	ok

2.15. Spectra of compound **9a**

^{1}H NMR spectrum (600 MHz, 25 ºC, DMSO-d_6)

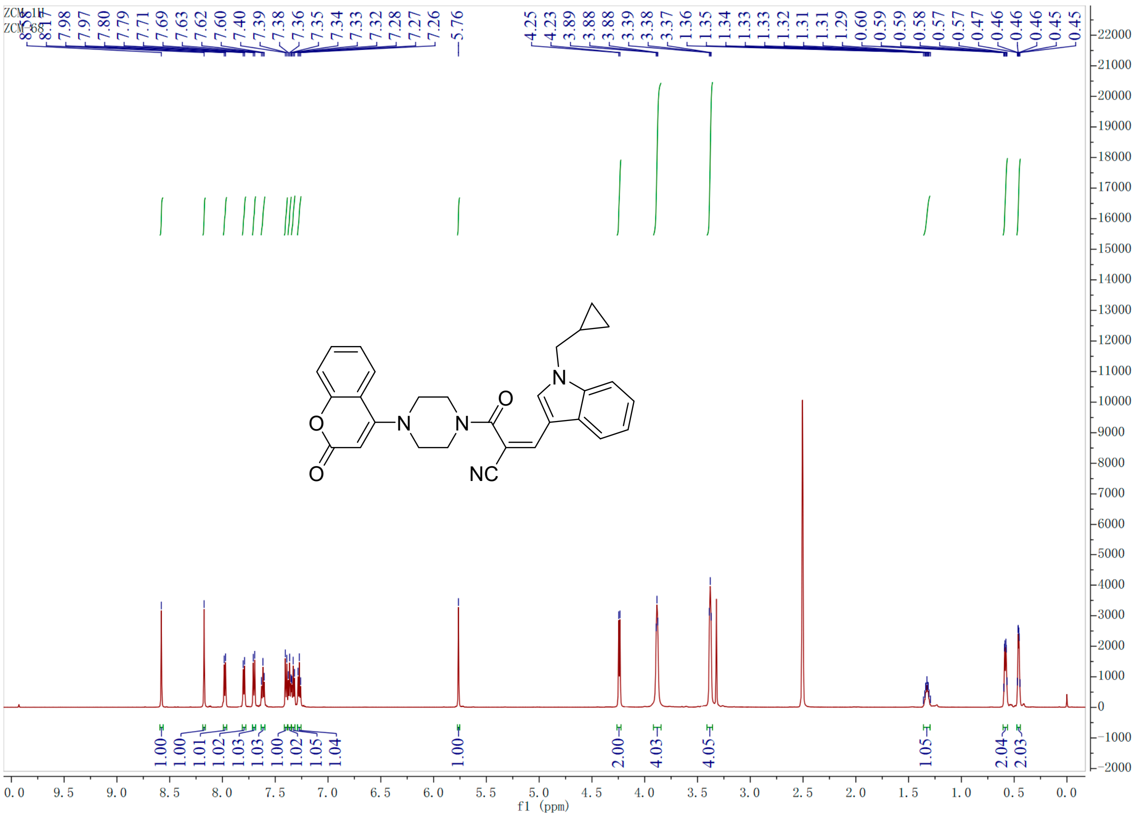

^{13}C NMR spectrum (151 MHz, 25 ºC, DMSO-d_6)

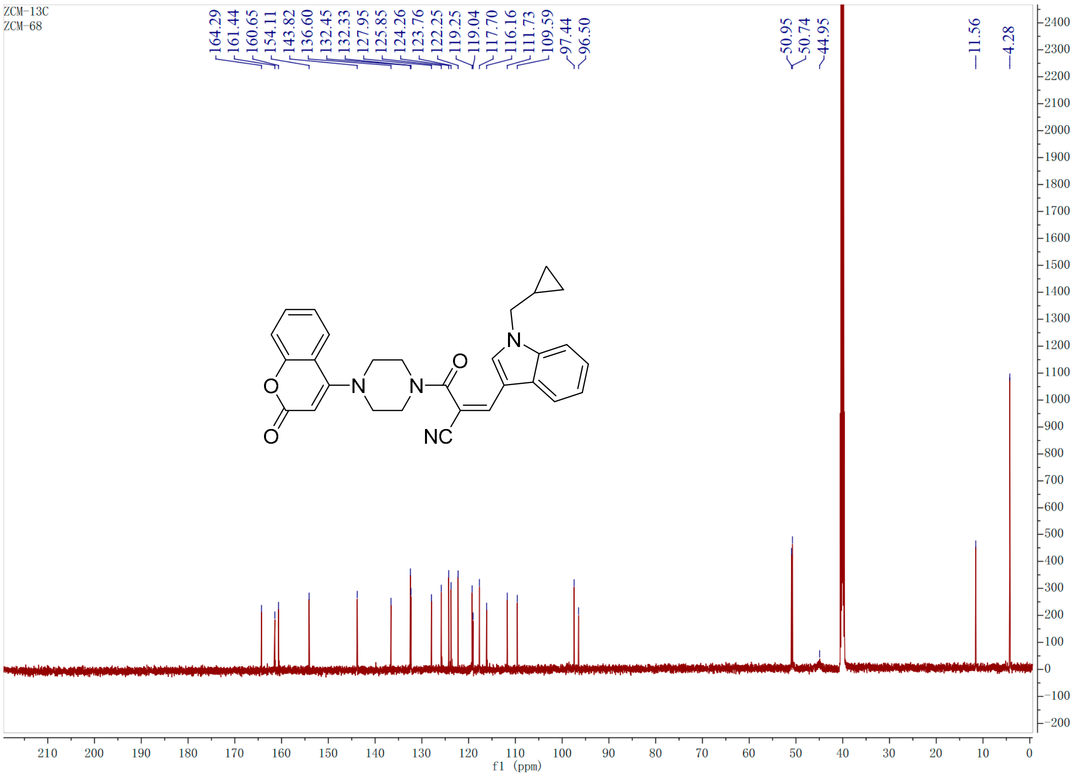

HRMS spectrum

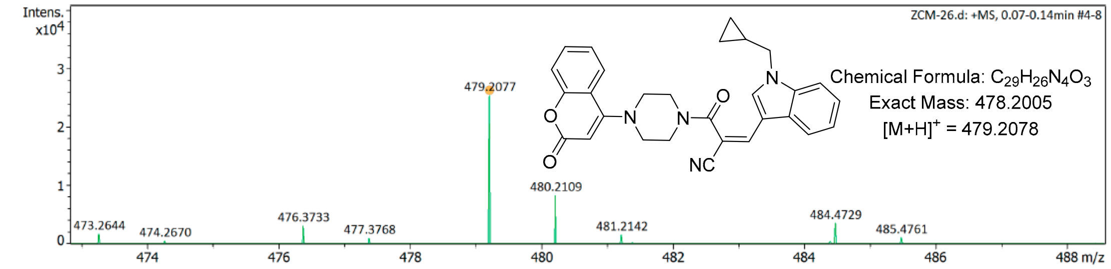

Meas. m/z	#	Ion Formula	Sum Formula	Adduct	m/z	err [ppm]	mSigma	# mSigma	Score	rdb	e¡¥ Conf	N-Rule
479.2077	1	C29H27N4O3	C29H26N4O3	M+H	479.2078	0.1	4.5	1	100.00	19.0	even	ok

2.16. Spectra of compound **9b**

^{1}H NMR spectrum (600 MHz, 25 °C, DMSO-d_6)

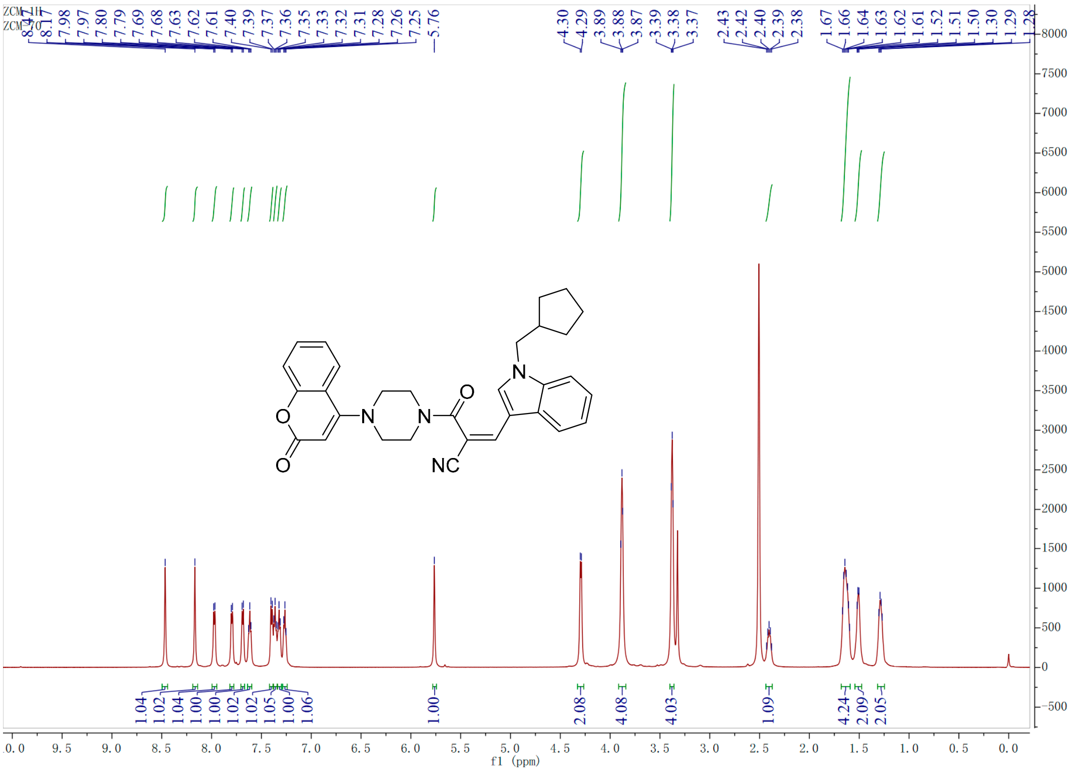

^{13}C NMR spectrum (151 MHz, 25 °C, DMSO-d_6)

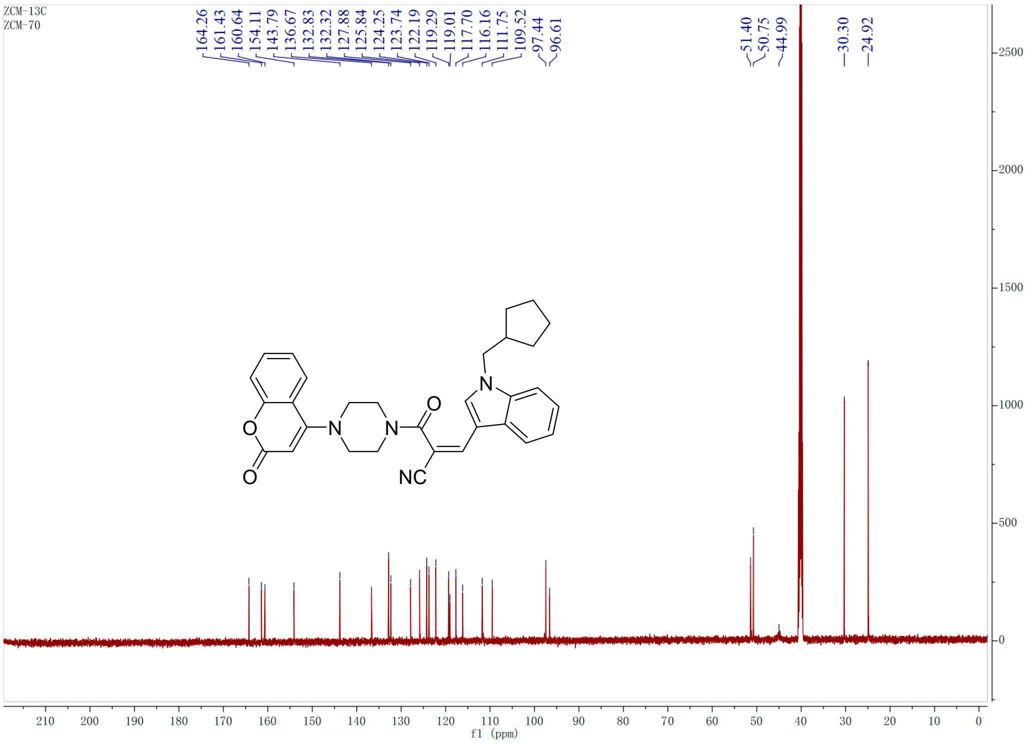

HRMS spectrum

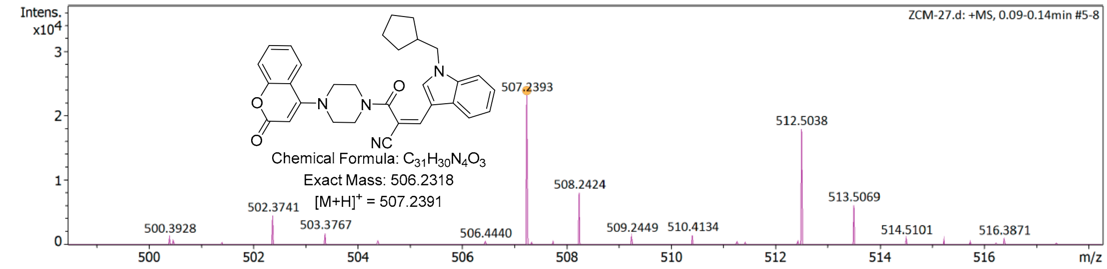

Meas. m/z	#	Ion Formula	Sum Formula	Adduct	m/z	err [ppm]	mSigma	# mSigma	Score	rdb	e¡¥ Conf	N-Rule
507.2393	1	C31H31N4O3	C31H30N4O3	M+H	507.2391	-0.4	7.8	1	100.00	19.0	even	ok

2.17. Spectra of compound **9c**

^{1}H NMR spectrum (600 MHz, 25 ºC, DMSO-d_6)

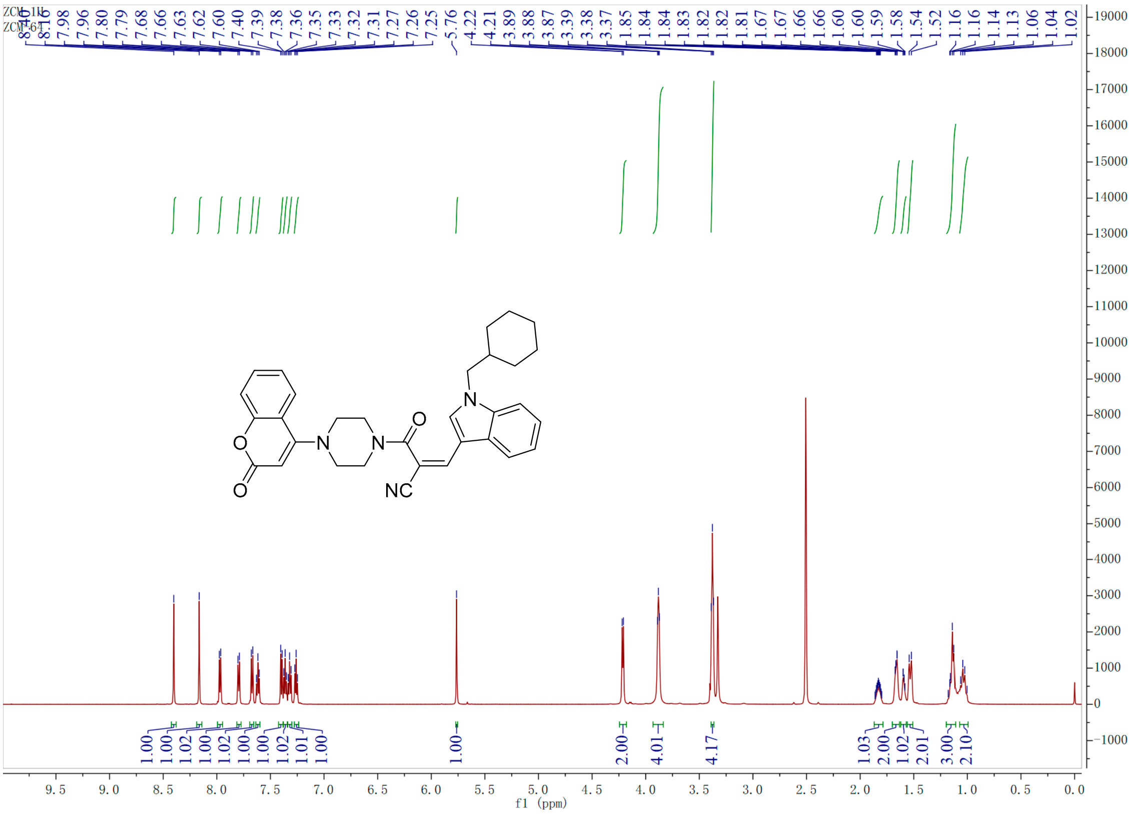

^{13}C NMR spectrum (151 MHz, 25 ºC, DMSO-d_6)

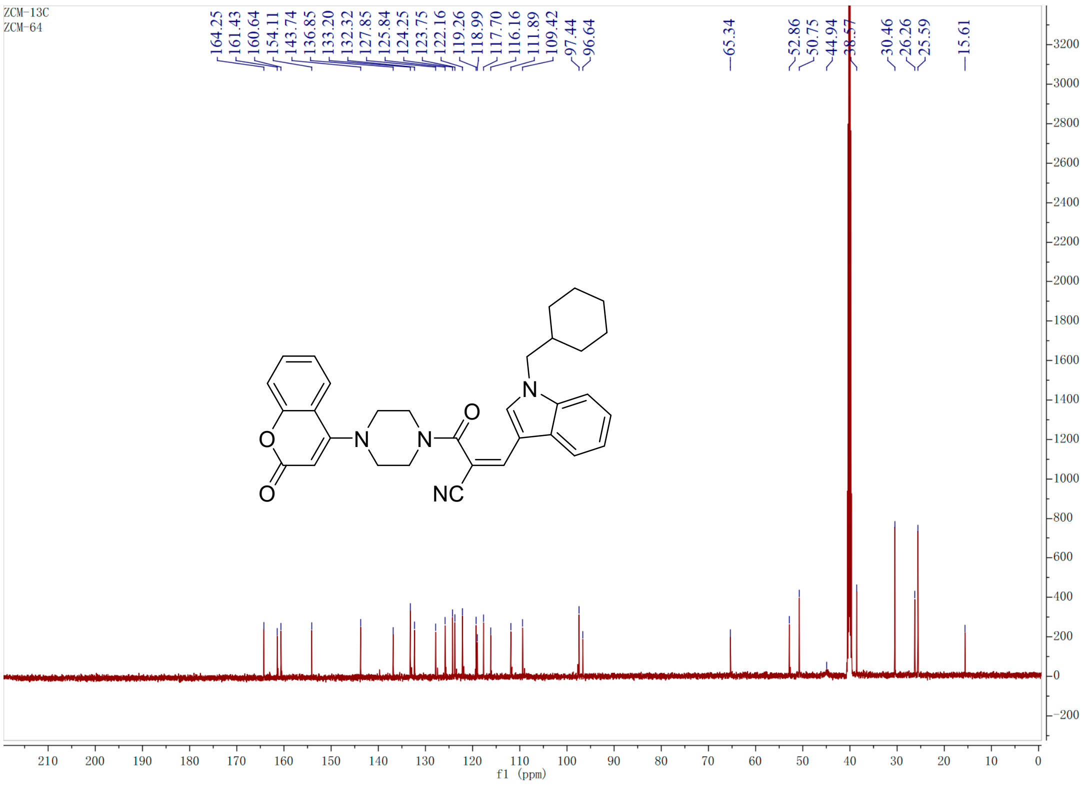

HRMS spectrum

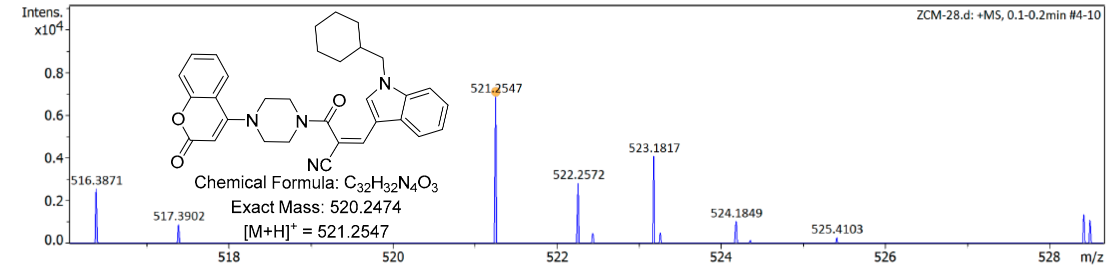

Meas. m/z	#	Ion Formula	Sum Formula	Adduct	m/z	err [ppm]	mSigma	# mSigma	Score	rdb	e¯ Conf	N-Rule
521.2547	1	C32H33N4O3	C32H32N4O3	M+H	521.2547	-0.0	24.1	1	100.00	19.0	even	ok

2.18. Spectra of compound **11a**

^{1}H NMR spectrum (600 MHz, 25 °C, DMSO-d_6)

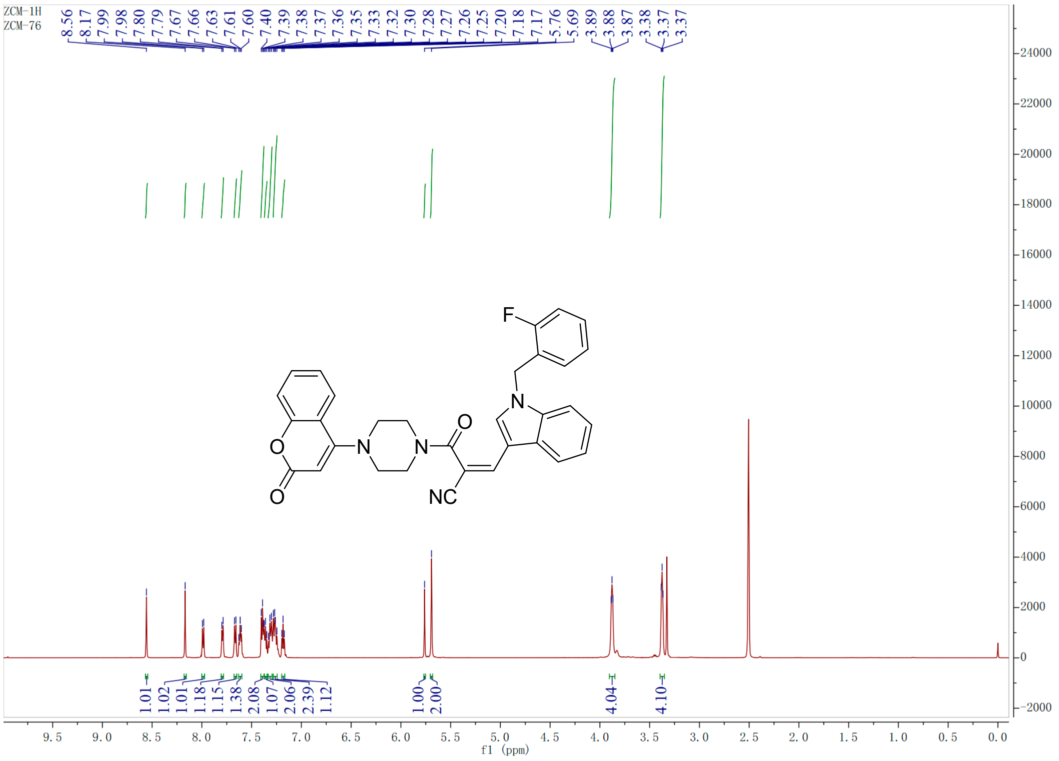

^{13}C NMR spectrum (151 MHz, 25 ºC, DMSO-d_6)

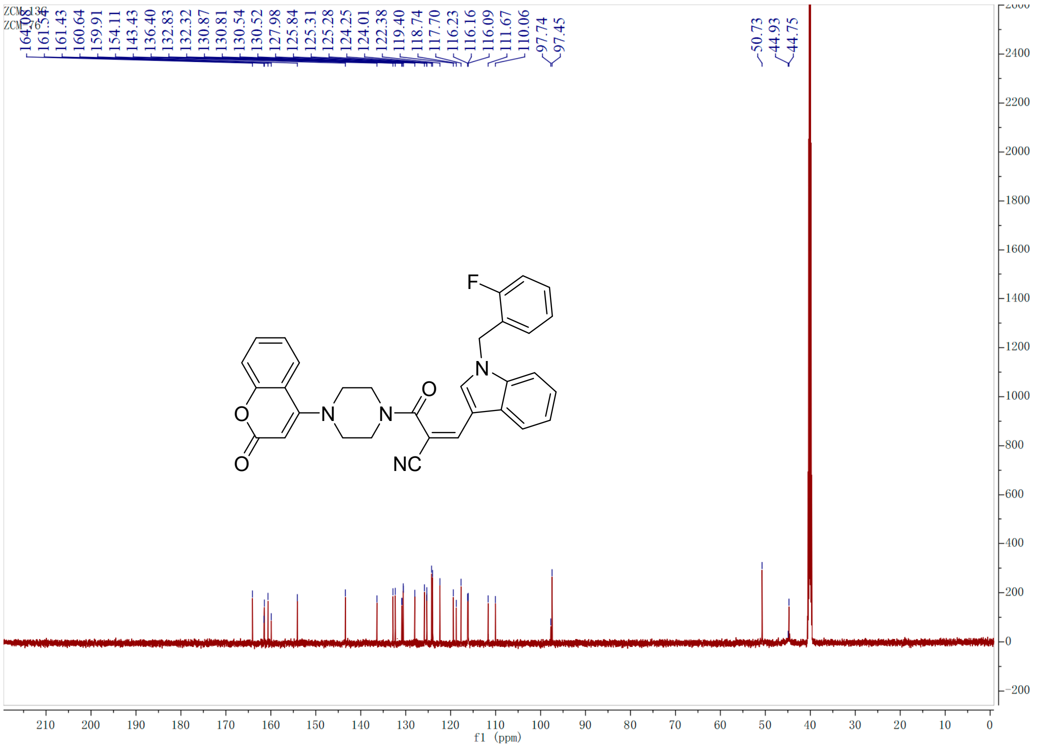

HRMS spectrum

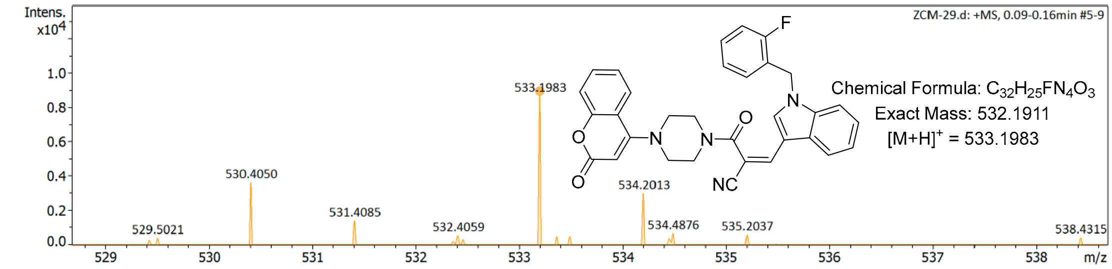

Meas. m/z	#	Ion Formula	Sum Formula	Adduct	m/z	err [ppm]	mSigma	# mSigma	Score	rdb	e¡¥ Conf	N-Rule
533.1983	1	C32H26FN4O3	C32H25FN4O3	M+H	533.1983	0.2	11.2	1	100.00	22.0	even	ok

2.19. Spectra of compound **11b**

^{1}H NMR spectrum (600 MHz, 25 ºC, DMSO-d_6)

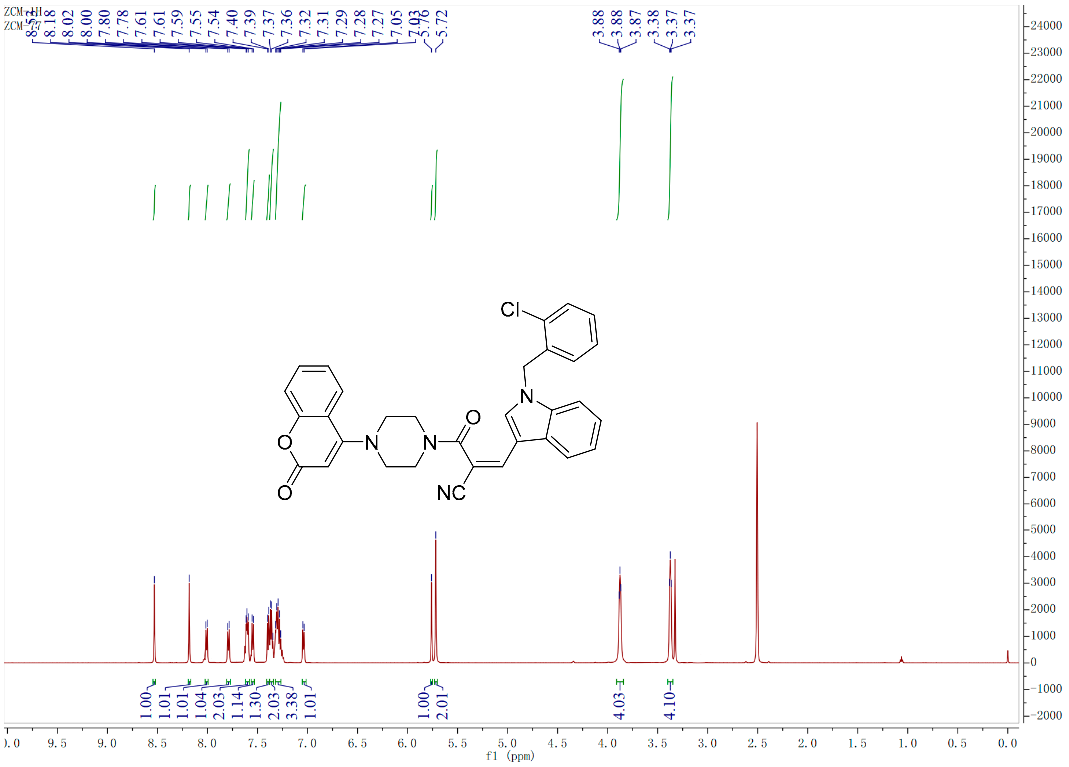

^{13}C NMR spectrum (151 MHz, 25 ºC, DMSO-d_6)

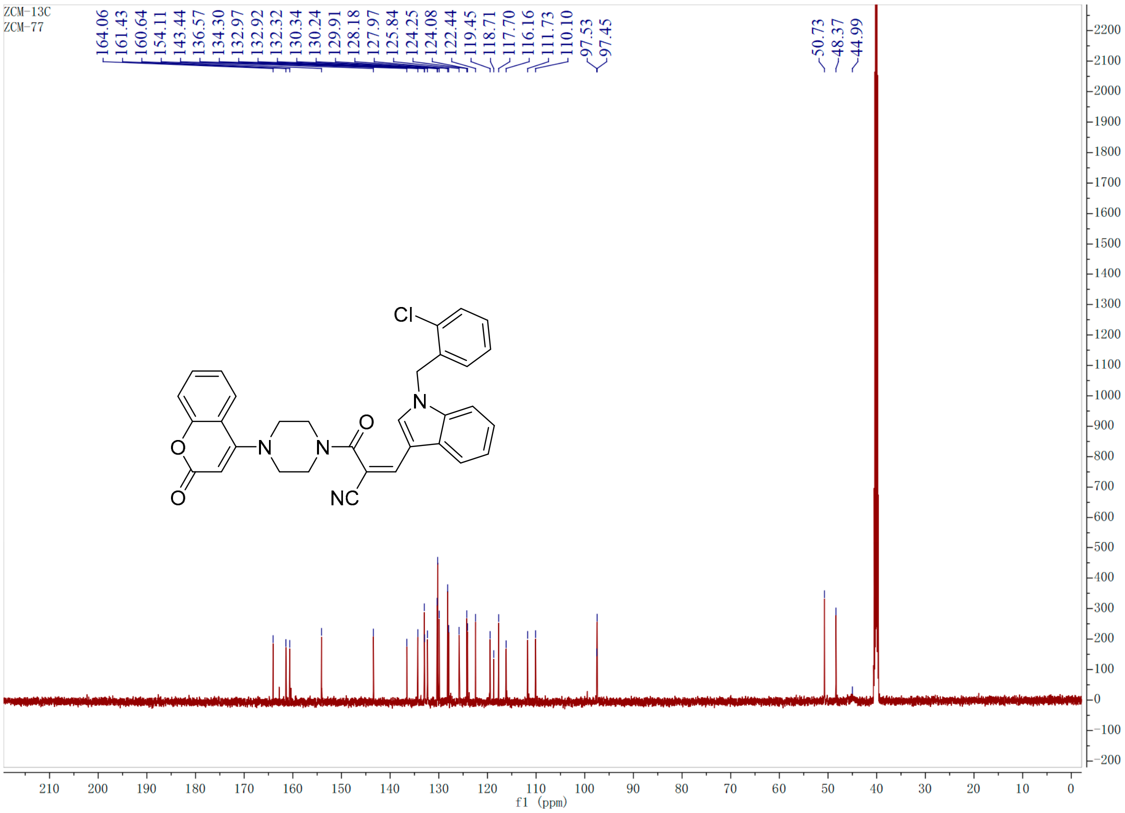

HRMS spectrum

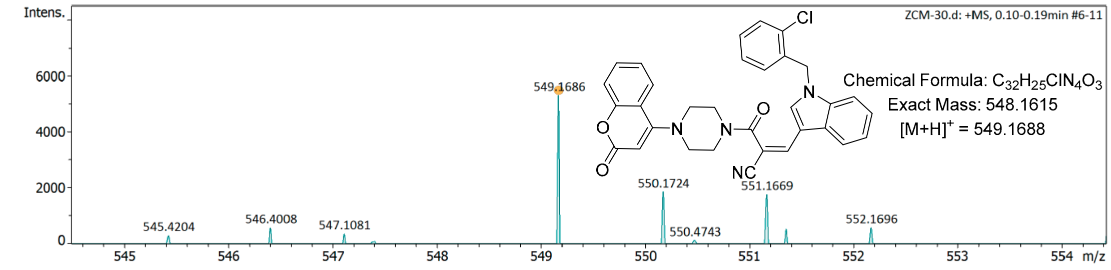

Meas. m/z	#	Ion Formula	Sum Formula	Adduct	m/z	err [ppm]	mSigma	# mSigma	Score	rdb	e¡¥ Conf	N-Rule
549.1686	1	C32H26ClN4O3	C32H25ClN4O3	M+H	549.1688	0.3	29.9	1	100.00	25.0	even	ok

2.20. Spectra of compound **11c**

^{1}H NMR spectrum (600 MHz, 25 °C, DMSO-d_6)

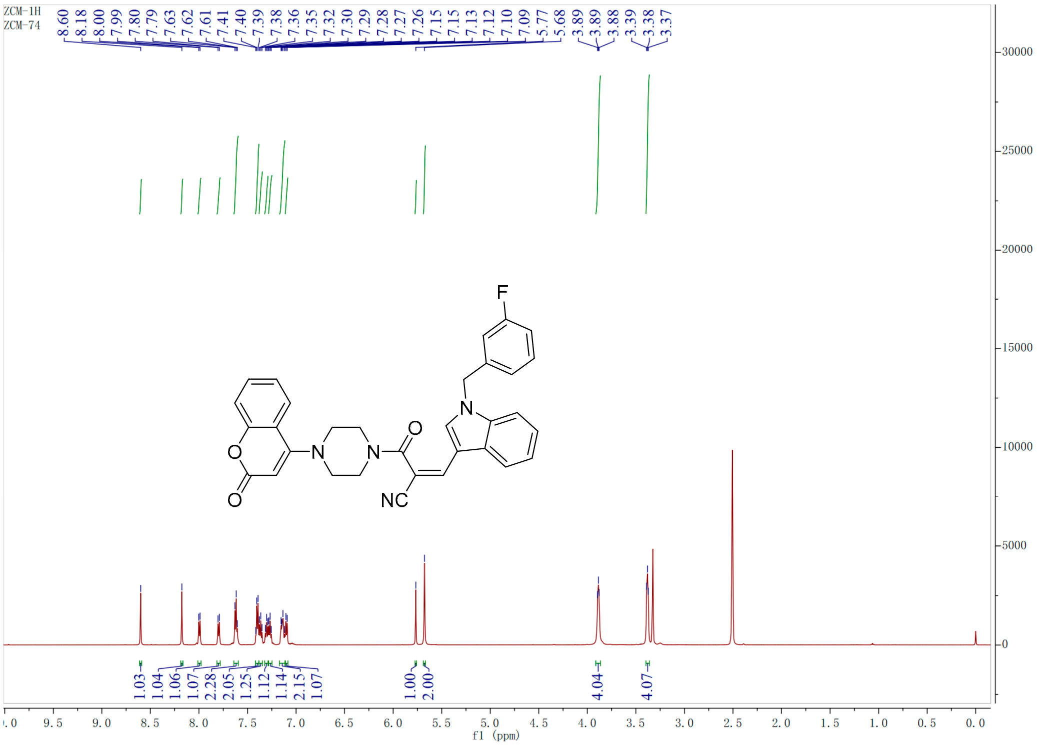

^{13}C NMR spectrum (151 MHz, 25 ºC, DMSO-d_6)

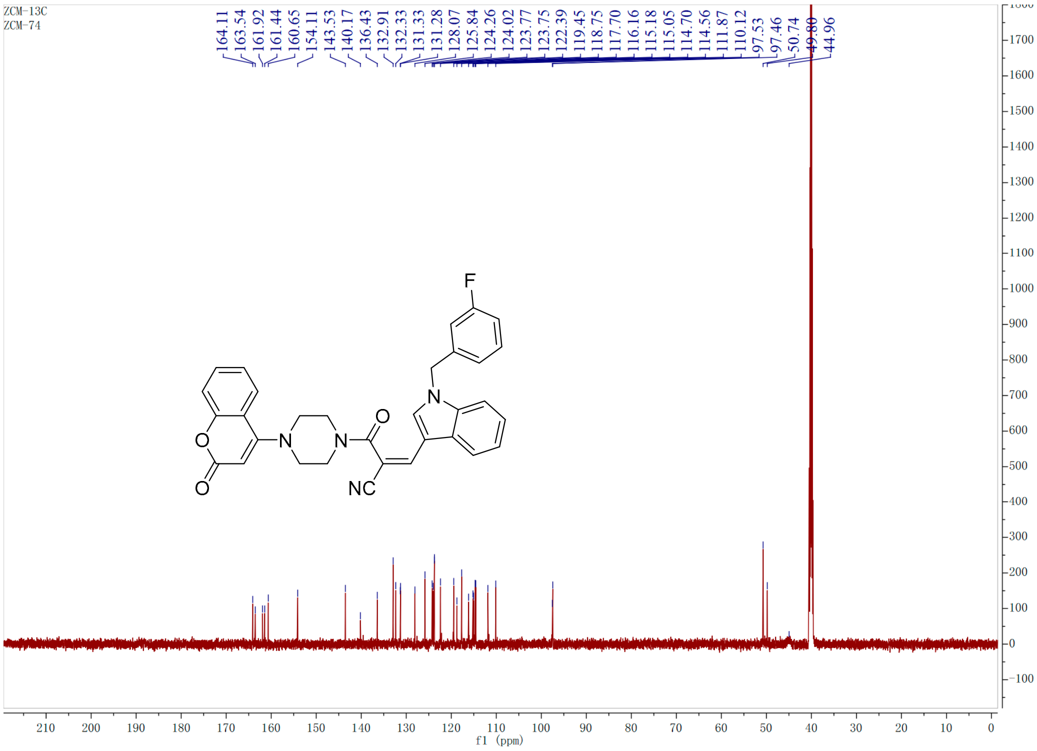

HRMS spectrum

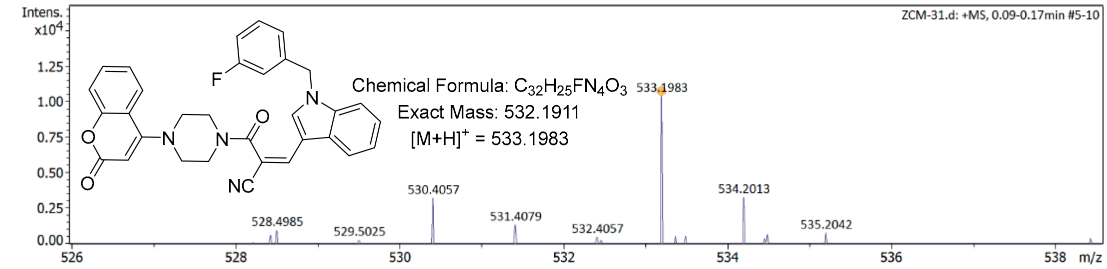

Meas. m/z	#	Ion Formula	Sum Formula	Adduct	m/z	err [ppm]	mSigma	# mSigma	Score	rdb	e¡¥ Conf	N-Rule
533.1983	1	C32H26FN4O3	C32H25FN4O3	M+H	533.1983	0.1	24.3	1	100.00	22.0	even	ok

2.21. Spectra of compound **11d**

^{1}H NMR spectrum (600 MHz, 25 ºC, DMSO-d_6)

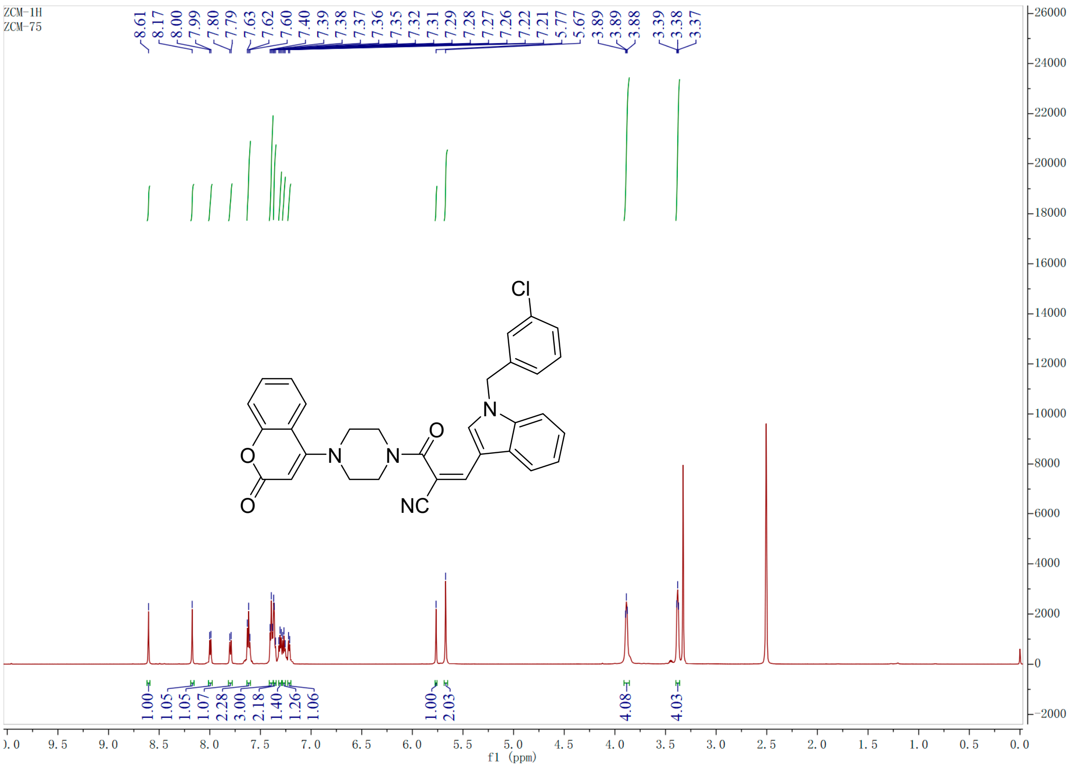

^{13}C NMR spectrum (151 MHz, 25 ºC, DMSO-d_6)

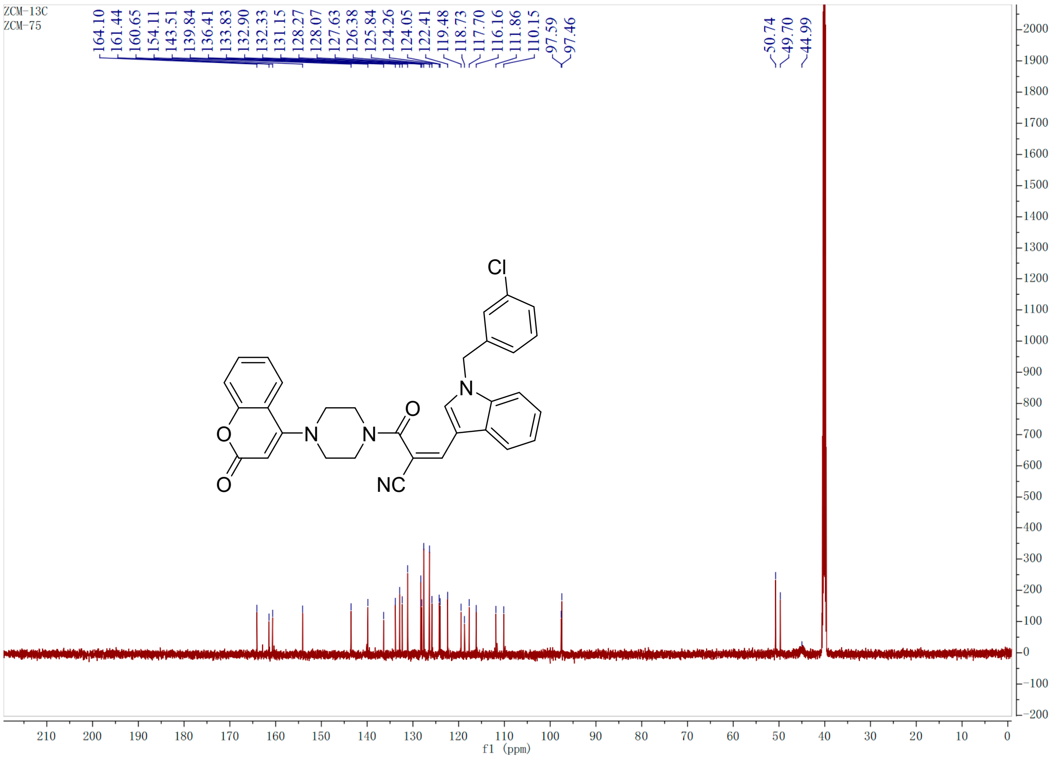

HRMS spectrum

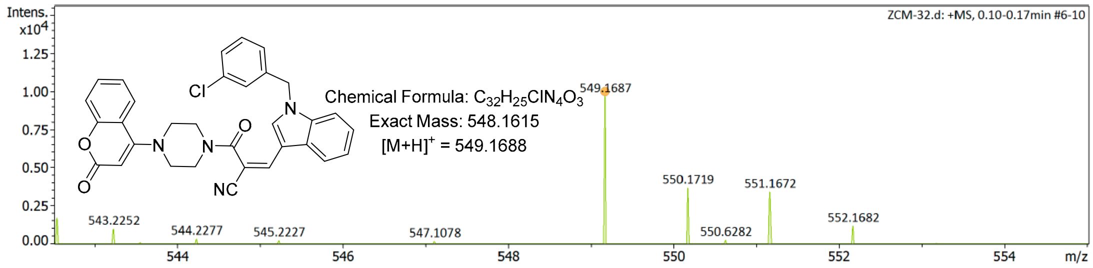

Meas. m/z	#	Ion Formula	Sum Formula	Adduct	m/z	err [ppm]	mSigma	# mSigma	Score	rdb	e¡¥ Conf	N-Rule
549.1687	1	C32H26ClN4O3	C32H25ClN4O3	M+H	549.1688	0.2	20.3	1	100.00	25.0	even	ok

2.22. Spectra of compound **11e**

^{1}H NMR spectrum (600 MHz, 25 °C, DMSO-d_6)

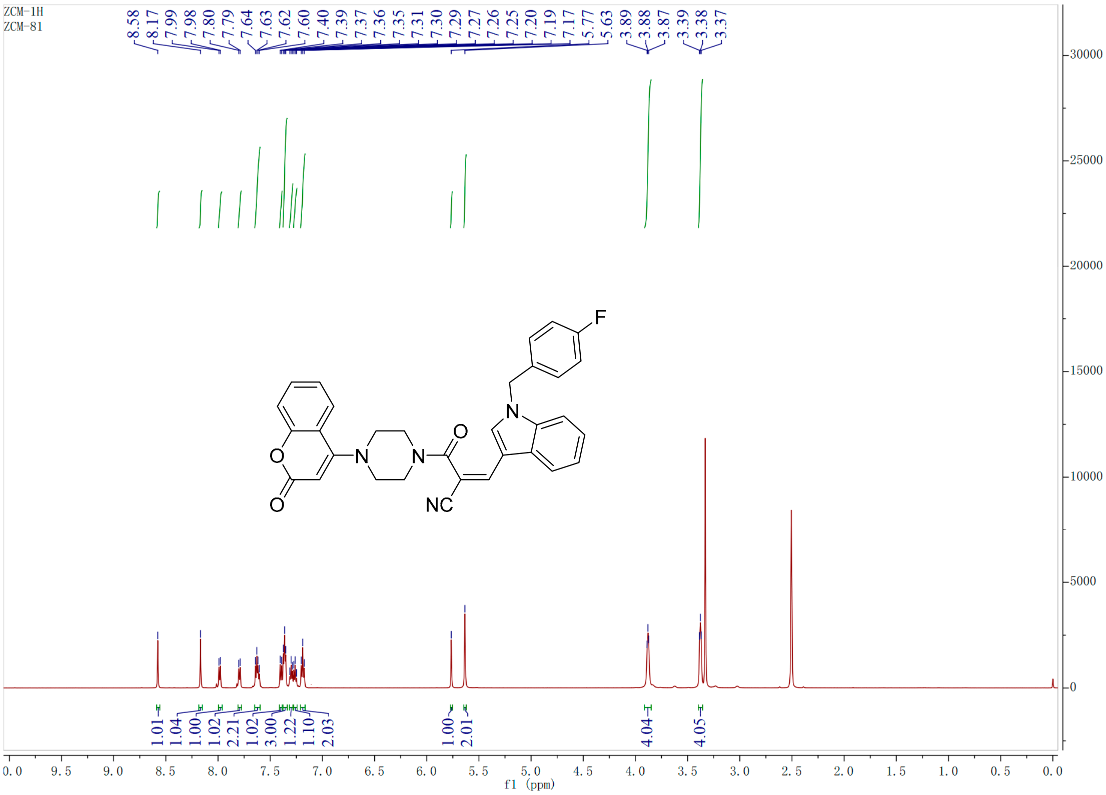

^{13}C NMR spectrum (151 MHz, 25 ºC, DMSO-d_6)

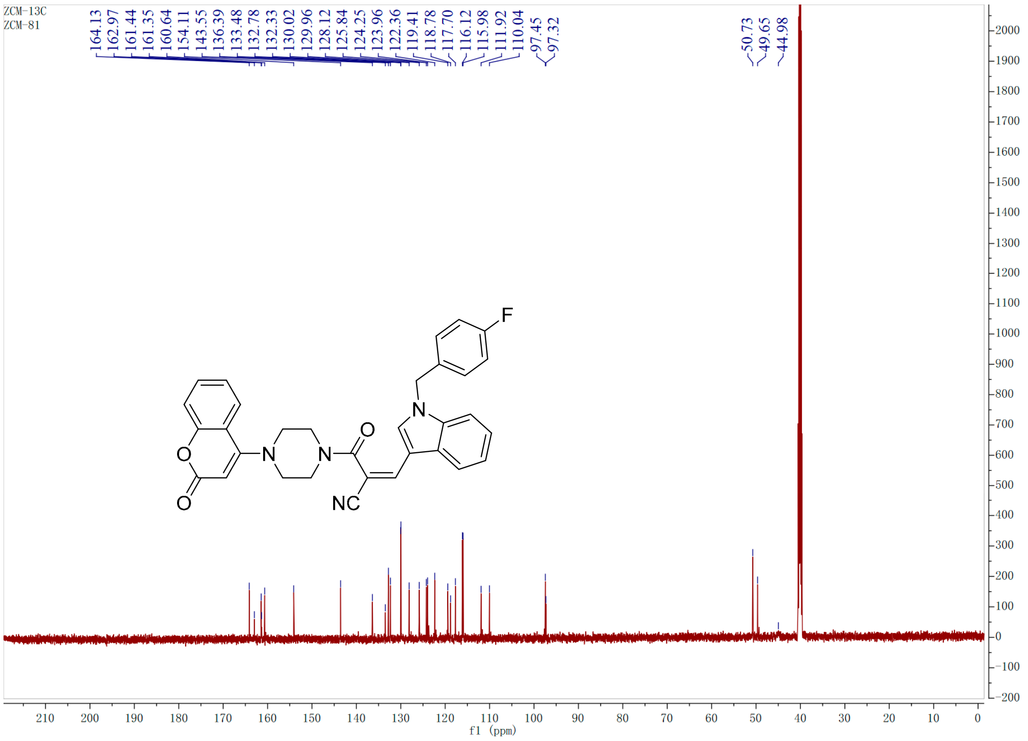

HRMS spectrum

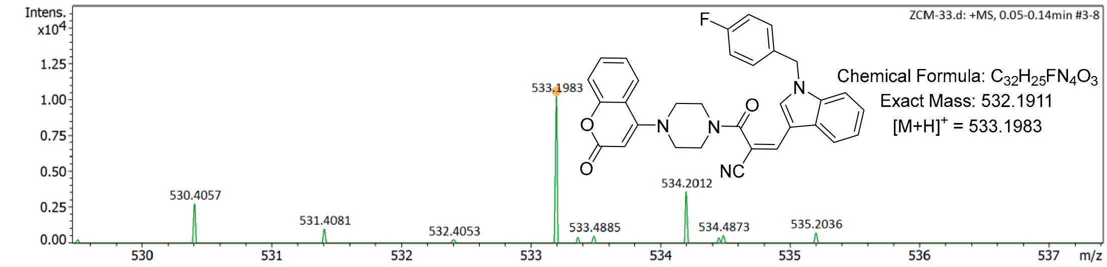

Meas. m/z	#	Ion Formula	Sum Formula	Adduct	m/z	err [ppm]	mSigma	# mSigma	Score	rdb	e¡¥ Conf	N-Rule
533.1983	1	C32H26FN4O3	C32H25FN4O3	M+H	533.1983	0.1	8.9	1	100.00	22.0	even	ok

2.23. Spectra of compound **11f**

^{1}H NMR spectrum (600 MHz, 25 ºC, DMSO-d_6)

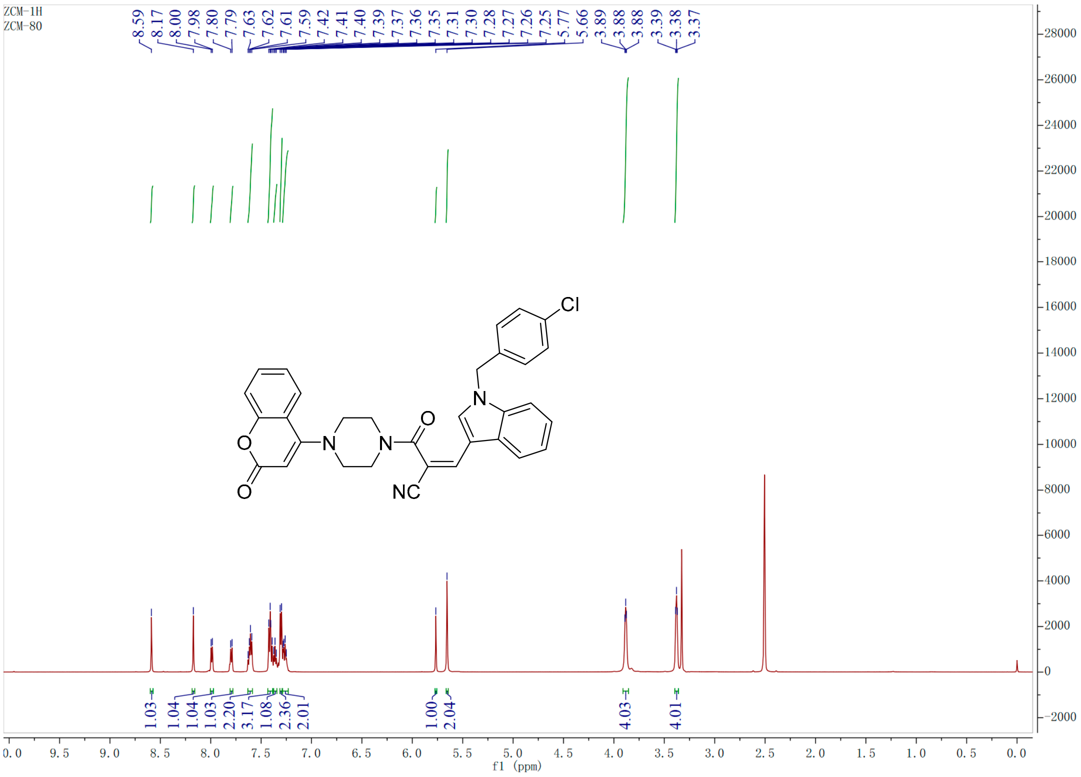

^{13}C NMR spectrum (151 MHz, 25 ºC, DMSO-d_6)

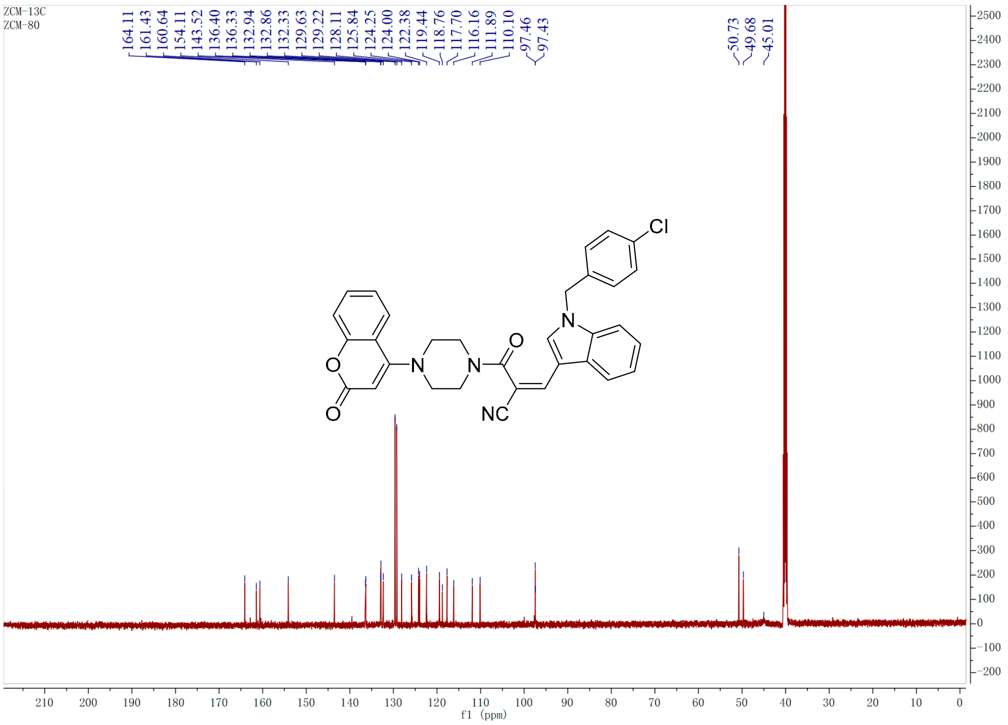

H-H COSY spectrum

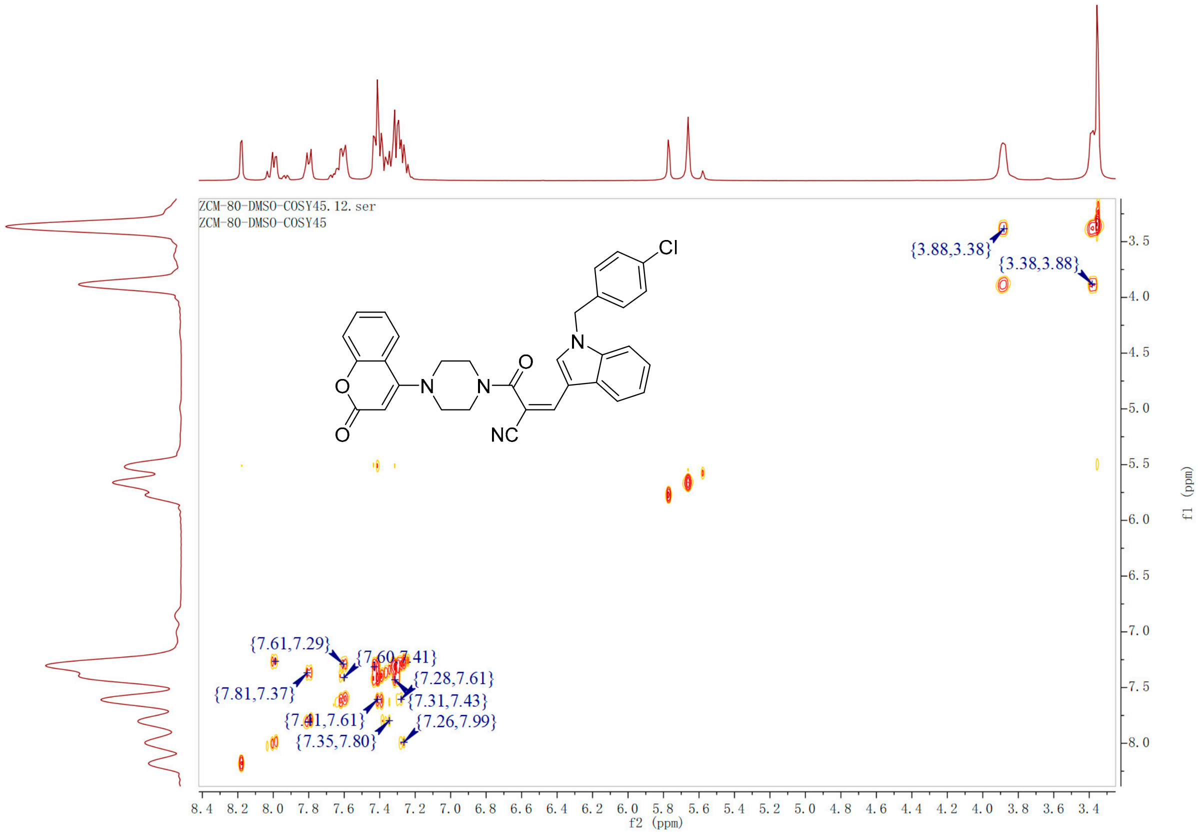

HMQC spectrum

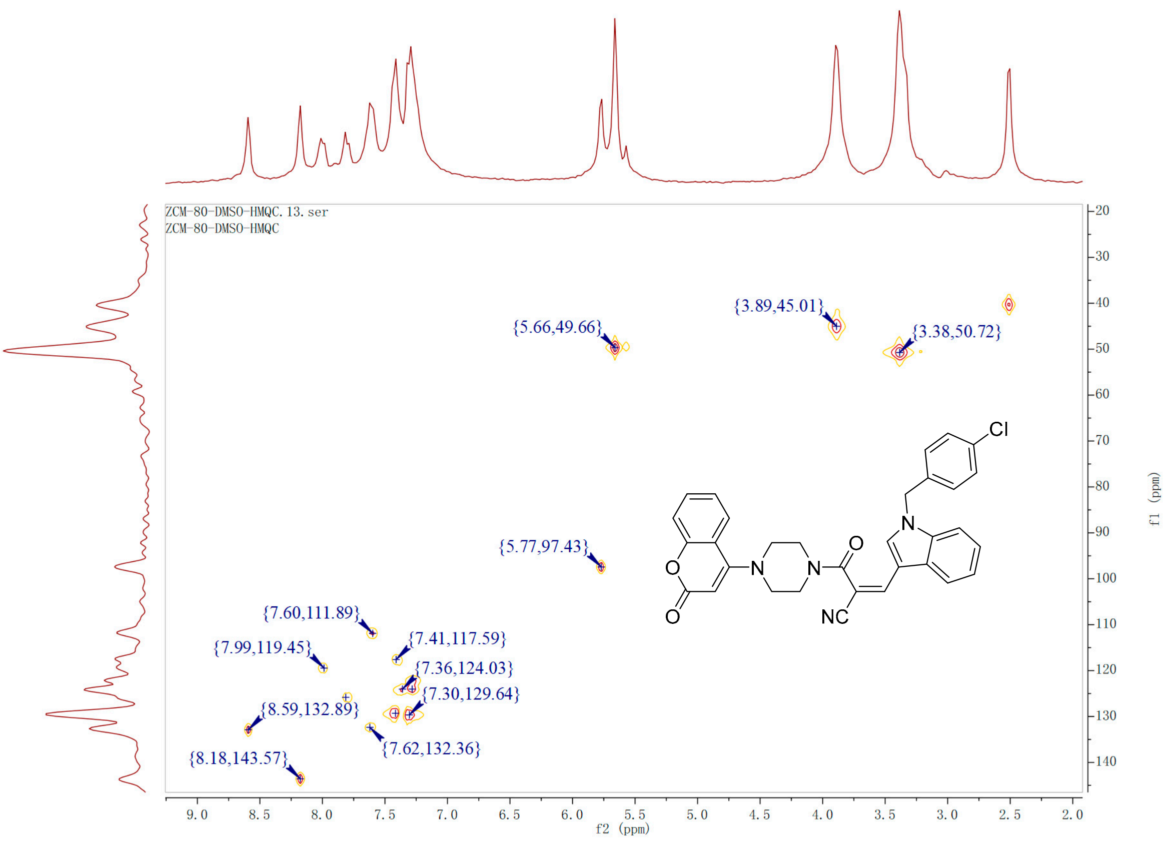

HMBC spectrum

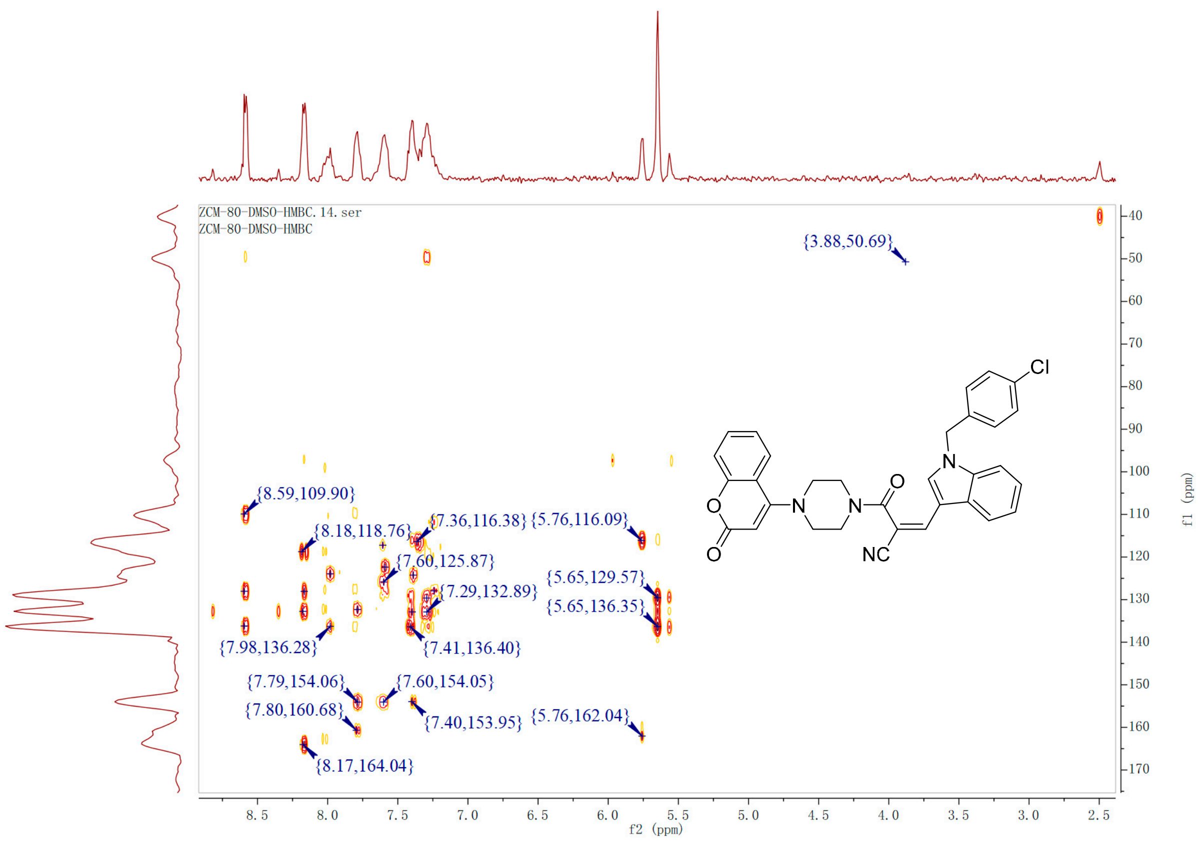

HRMS spectrum

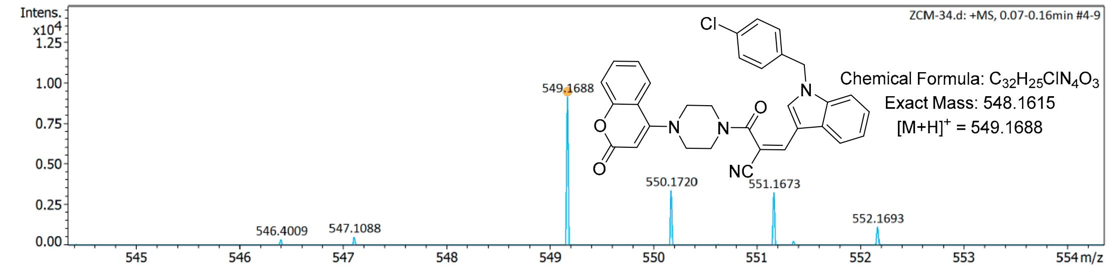

Meas. m/z	#	Ion Formula	Sum Formula	Adduct	m/z	err [ppm]	mSigma	# mSigma	Score	rdb	e¡¥ Conf	N-Rule
549.1688	1	C32H26ClN4O3	C32H25ClN4O3	M+H	549.1688	-0.1	18.9	1	100.00	25.0	even	ok

2.24. Spectra of compound **11g**

^{1}H NMR spectrum (400 MHz, 25 °C, DMSO-d_6)

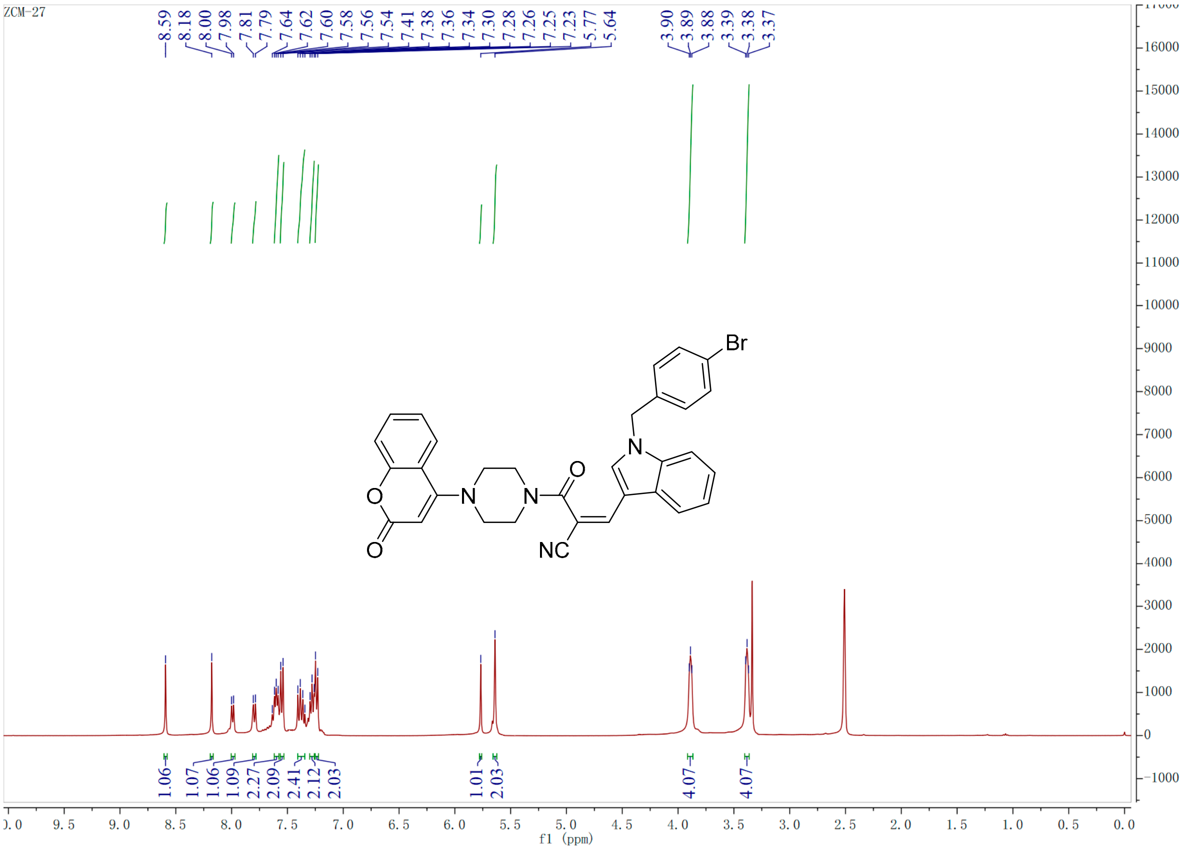

^{13}C NMR spectrum (101 MHz, 25 °C, DMSO-d_6)

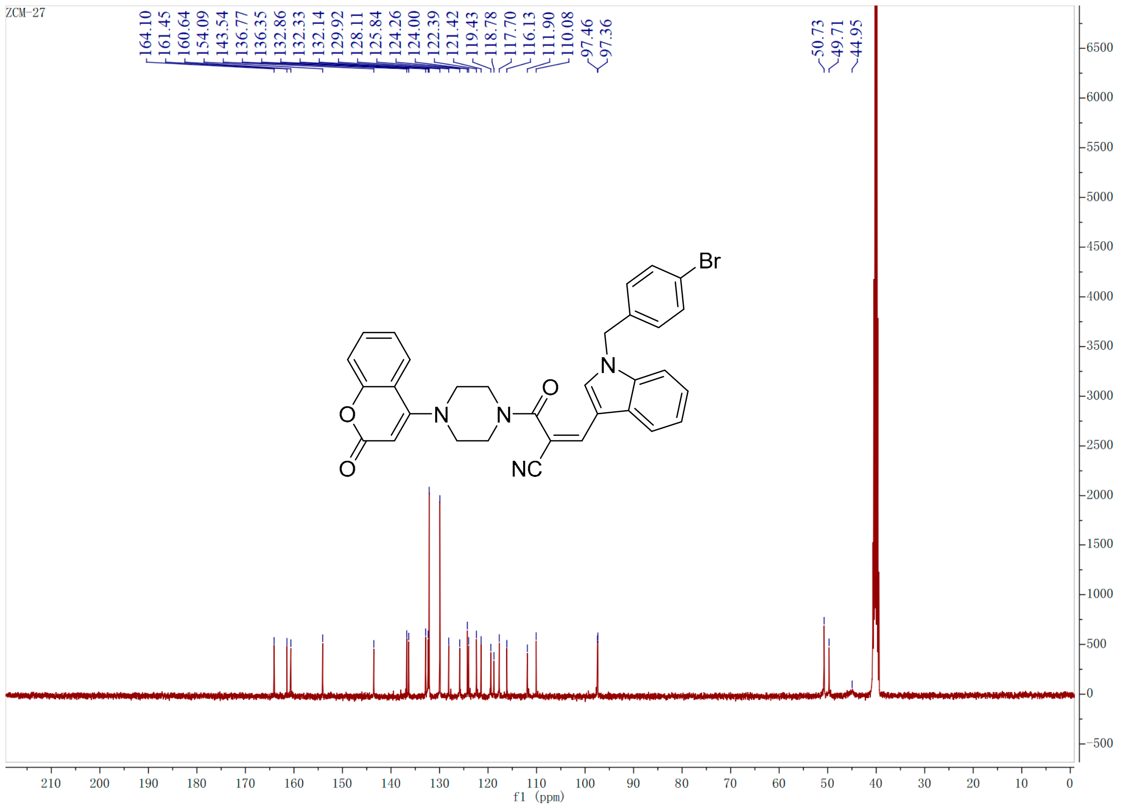

HRMS spectrum

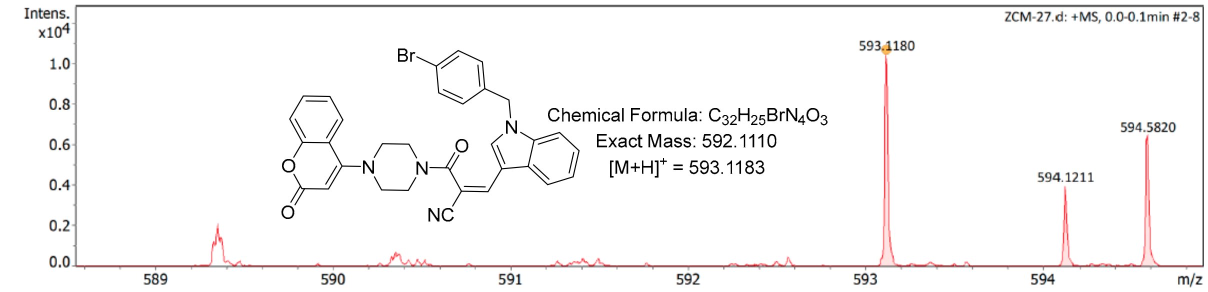

Meas. m/z	#	Sum Formula	Adduct	Ion Formula	m/z	err [ppm]	mSigma	# mSigma	Score	rdb	e¡¥ Conf	N-Rule
593.1180	1	C32H25BrN4O3	M+H	C32H26BrN4O3	593.1183	0.4	43.7	1	100.00	24.0	even	ok

2.25. Spectra of compound **11h**

^{1}H NMR spectrum (400 MHz, 25 °C, DMSO-d_6)

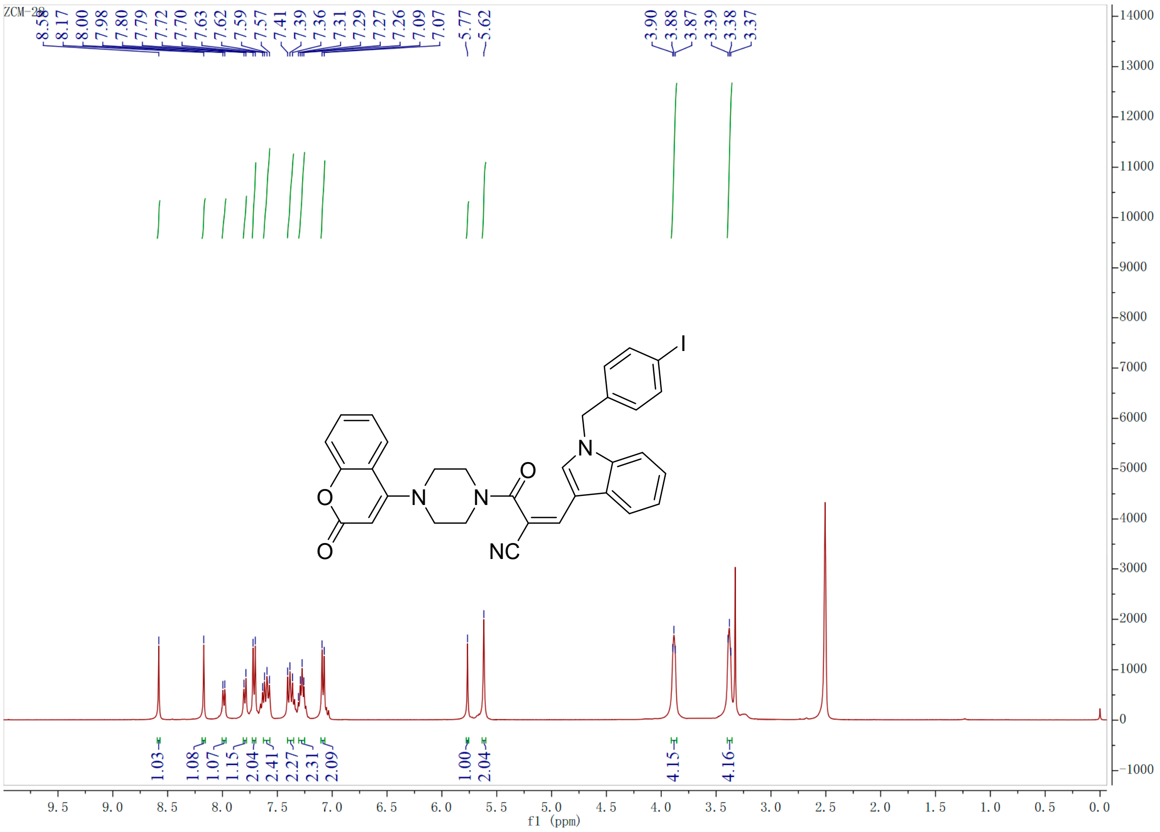

^{13}C NMR spectrum (101 MHz, 25 ºC, DMSO-d_6)

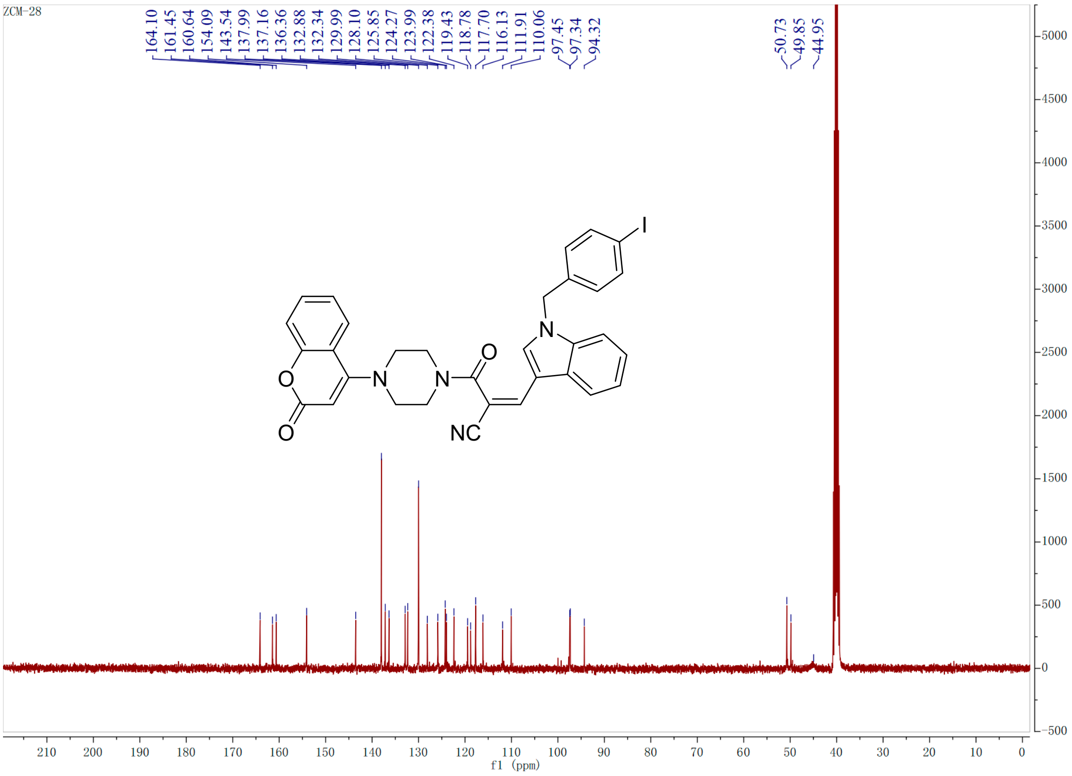

HRMS spectrum

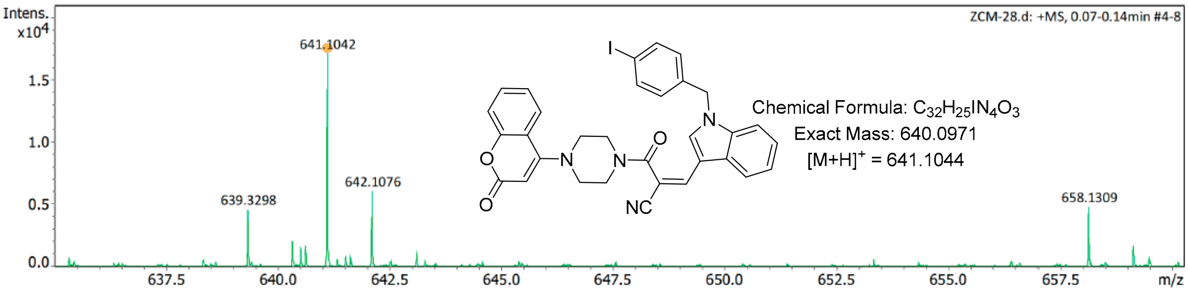

Meas. m/z	#	Sum Formula	Adduct	Ion Formula	m/z	err [ppm]	mSigma	# mSigma	Score	rdb	e¡¥ Conf	N-Rule
641.1042	1	C32H25IN4O3	M+H	C32H26IN4O3	641.1044	0.4	36.1	1	100.00	25.0	even	ok

2.26. Spectra of compound **11i**

^{1}H NMR spectrum (600 MHz, 25 ºC, DMSO-d_6)

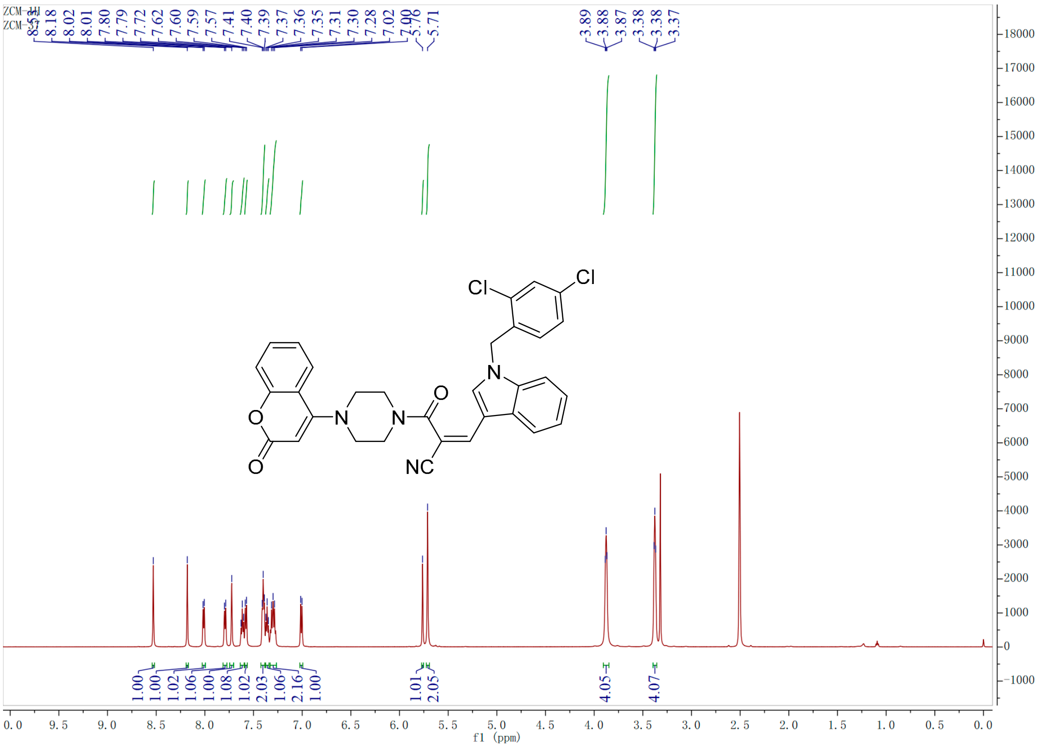

^{13}C NMR spectrum (151 MHz, 25 ºC, DMSO-d_6)

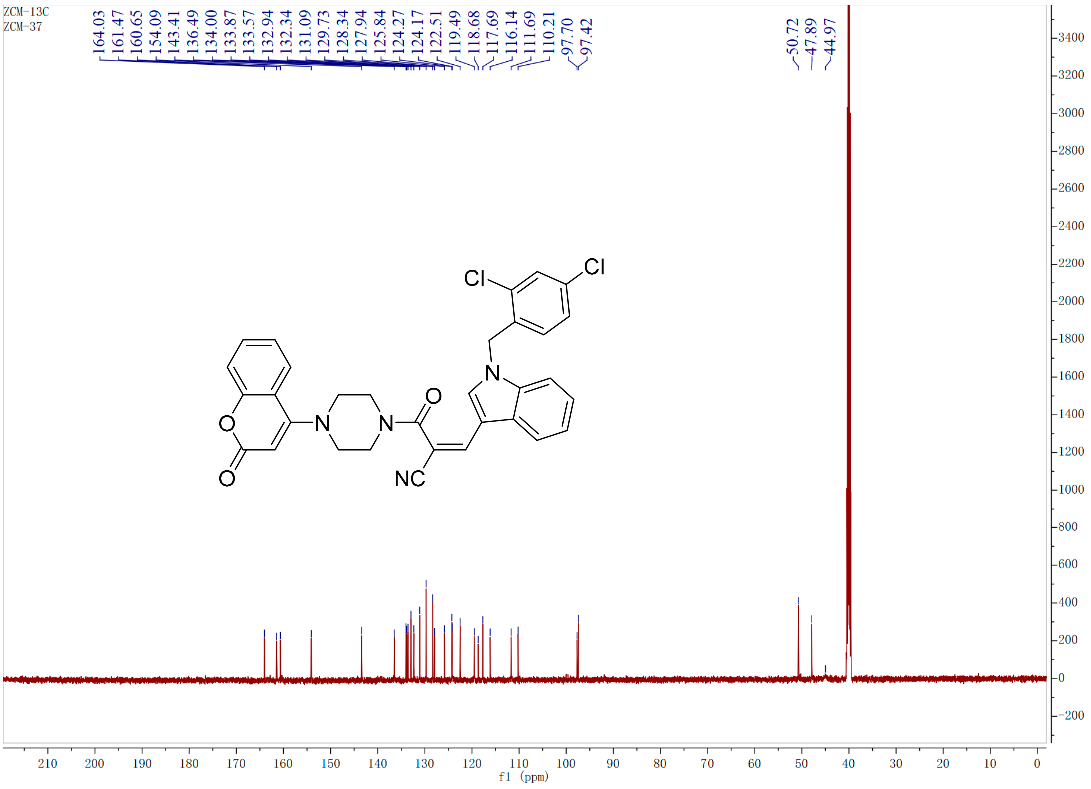

HRMS spectrum

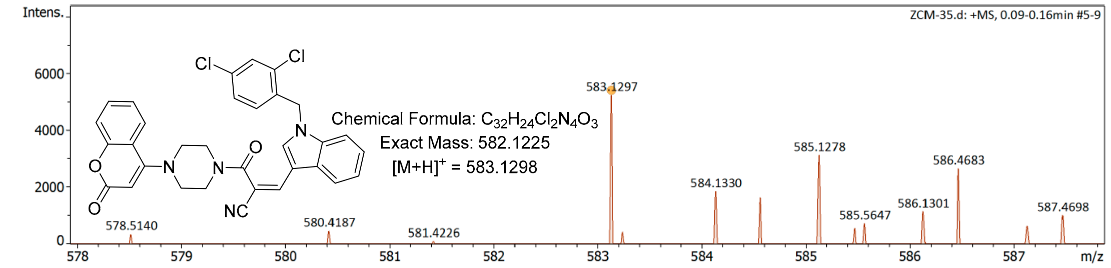

Meas. m/z	#	Ion Formula	Sum Formula	Adduct	m/z	err [ppm]	mSigma	# mSigma	Score	rdb	e¡¥ Conf	N-Rule
583.1297	1	C32H25Cl2N4O3	C32H24Cl2N4O3	M+H	583.1298	0.1	50.8	1	100.00	28.0	even	ok

2.27. Spectra of compound **13a**

^{1}H NMR spectrum (600 MHz, 25 °C, DMSO-d_6)

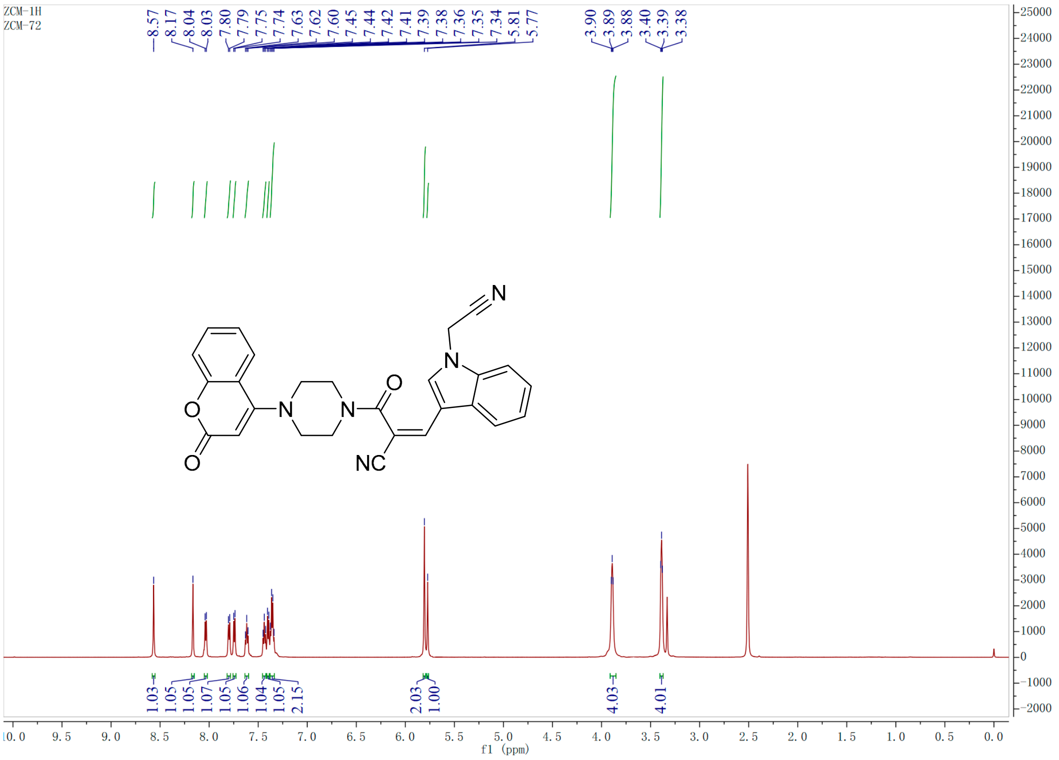

^{13}C NMR spectrum (151 MHz, 25 ºC, DMSO-d_6)

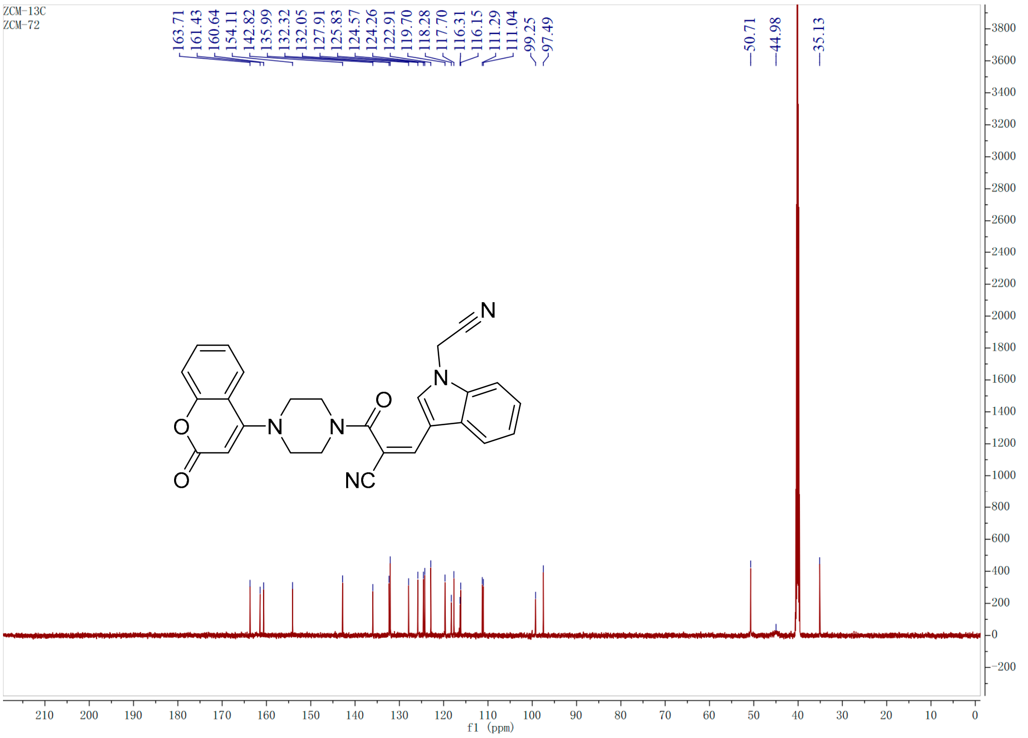

HRMS spectrum

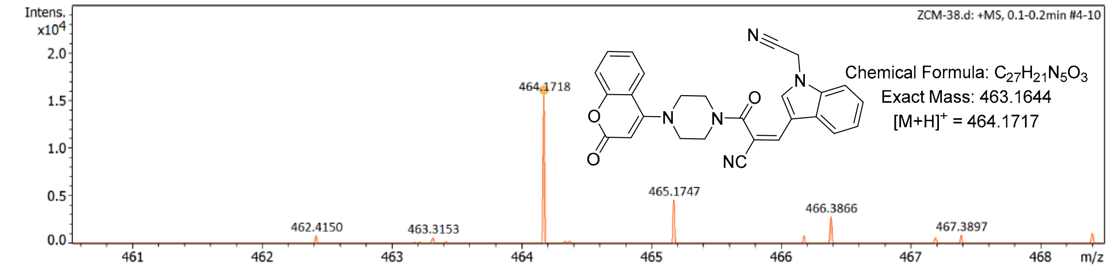

Meas. m/z	#	Ion Formula	Sum Formula	Adduct	m/z	err [ppm]	mSigma	# mSigma	Score	rdb	e¦¥ Conf	N-Rule
464.1718	1	C27H22N5O3	C27H21N5O3	M+H	464.1717	-0.2	9.4	1	100.00	20.0	even	ok

2.28. Spectra of compound **13b**

^{1}H NMR spectrum (600 MHz, 25 ºC, DMSO-d_6)

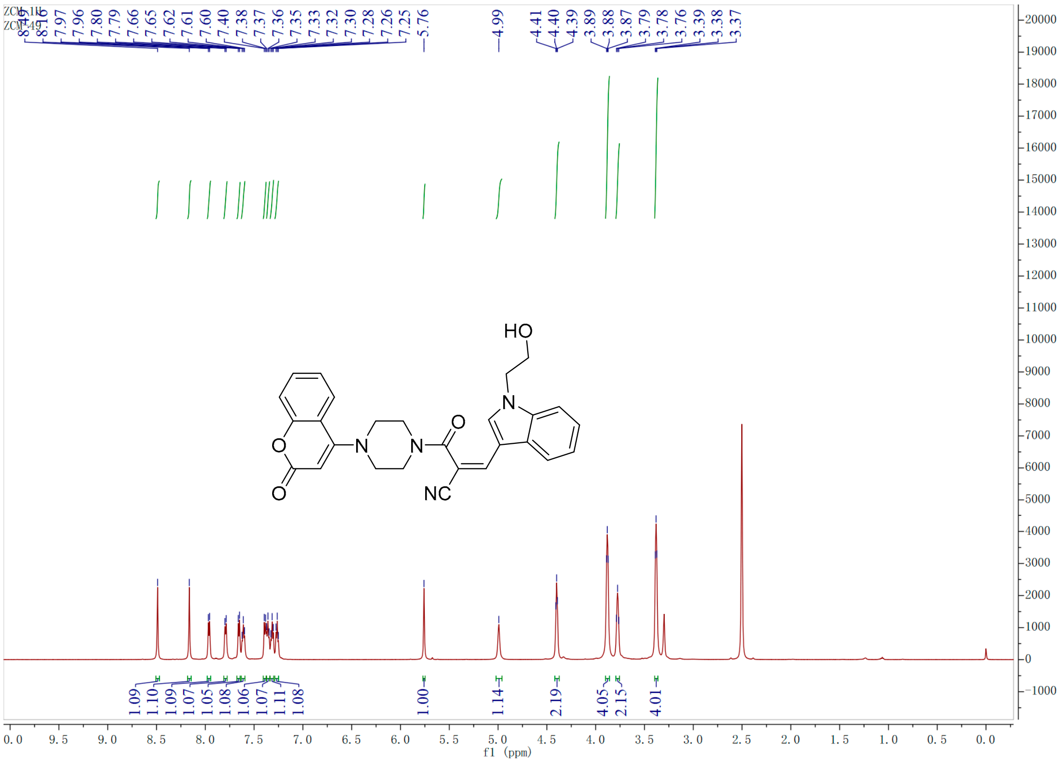

^{13}C NMR spectrum (151 MHz, 25 ºC, DMSO-d_6)

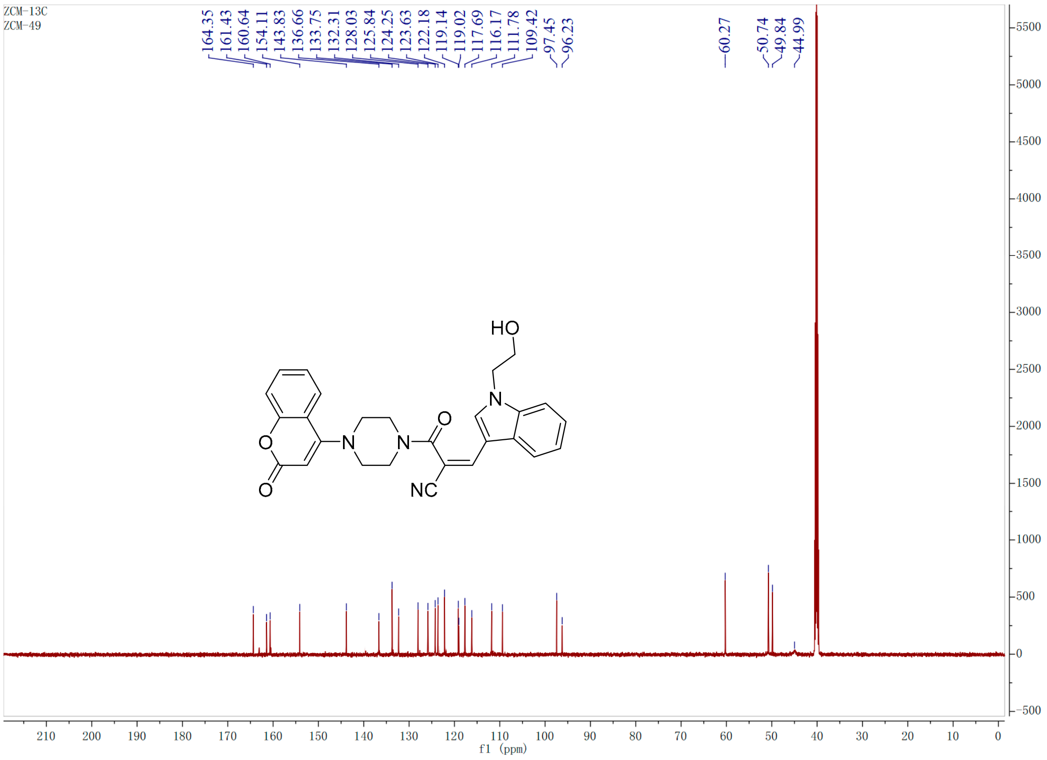

HMQC spectrum

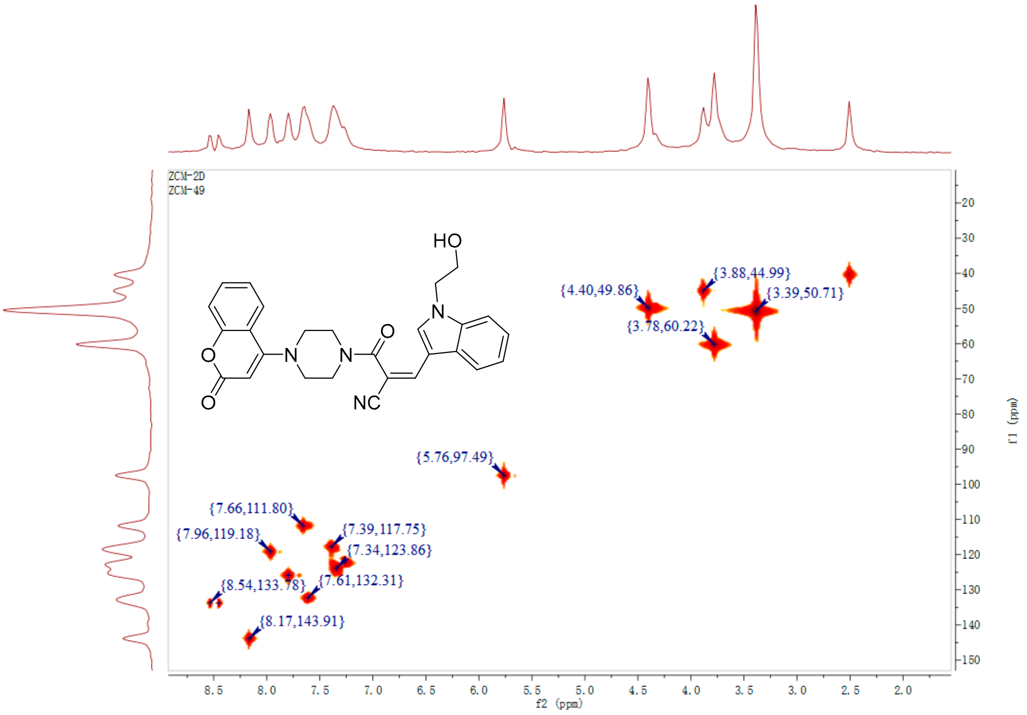

HRMS spectrum

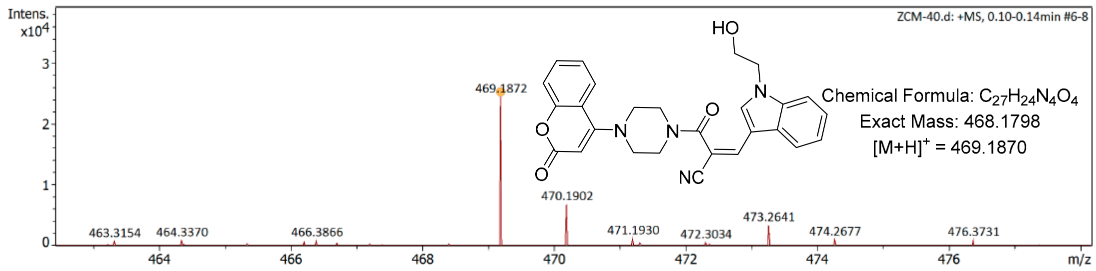

Meas. m/z	#	Ion Formula	Sum Formula	Adduct	m/z	err [ppm]	mSigma	# mSigma	Score	rdb	e¦¥ Conf	N-Rule
469.1872	1	C27H25N4O4	C27H24N4O4	M+H	469.1870	-0.4	16.7	1	100.00	18.0	even	ok

2.29. Spectra of compound **13c**

^{1}H NMR spectrum (600 MHz, 25 ºC, DMSO-d_6)

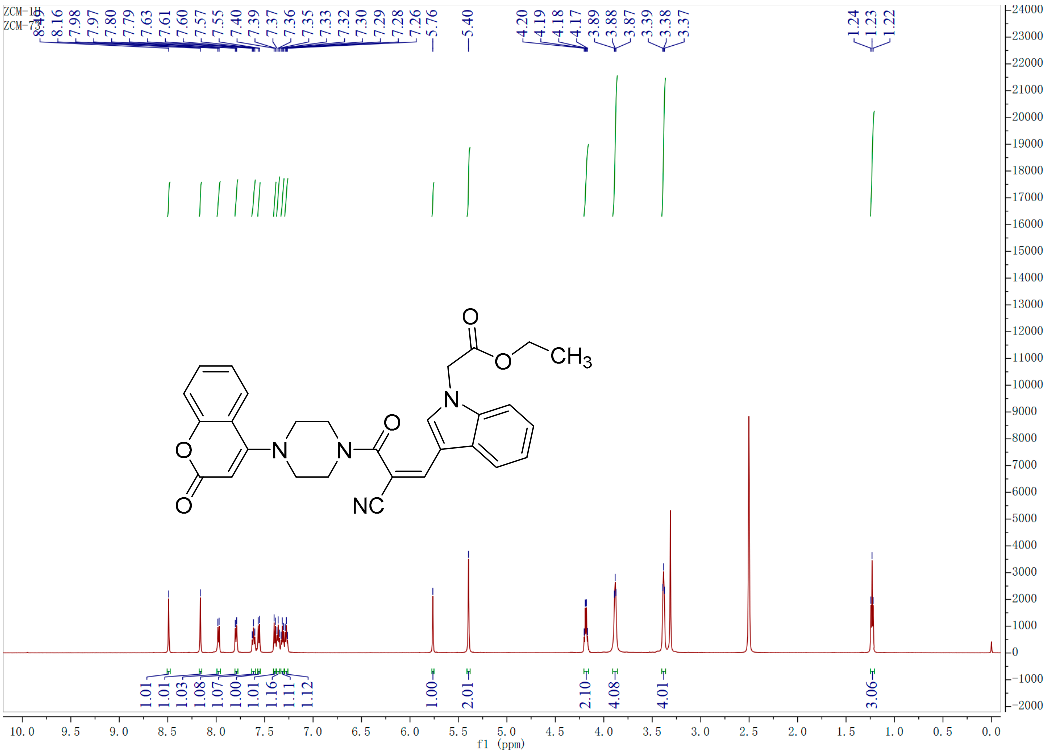

^{13}C NMR spectrum (151 MHz, 25 ºC, DMSO-d_6)

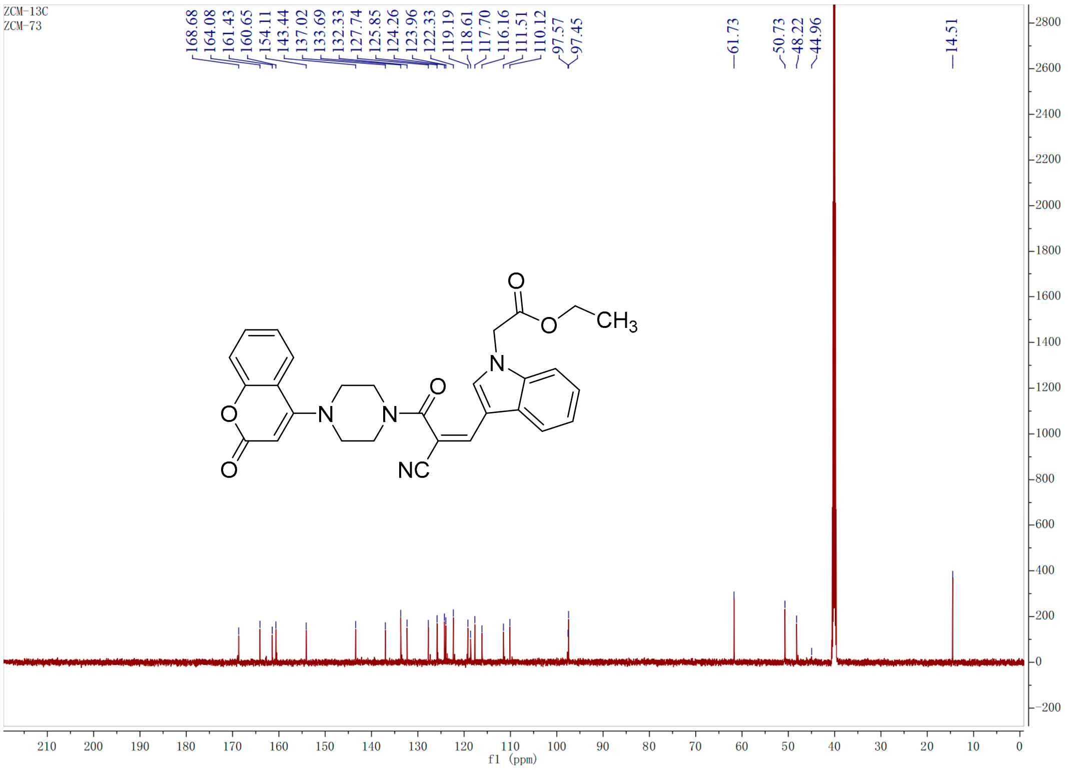

HRMS spectrum

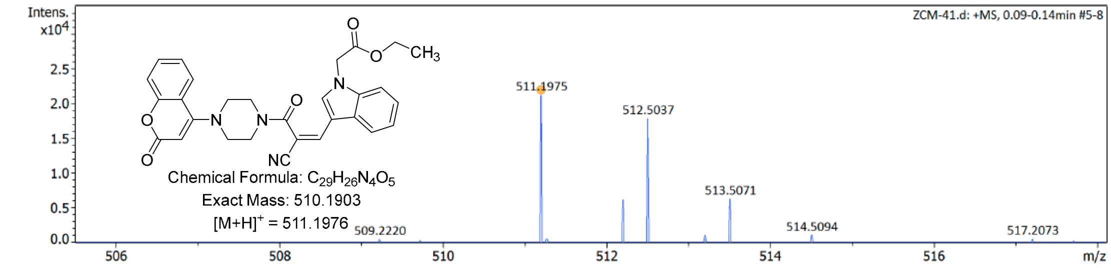

Meas. m/z	#	Ion Formula	Sum Formula	Adduct	m/z	err [ppm]	mSigma	# mSigma	Score	rdb	e¡¥ Conf	N-Rule
511.1975	1	C29H27N4O5	C29H26N4O5	M+H	511.1976	0.2	21.7	1	100.00	19.0	even	ok
